# Optimized Suspension Trapping Method for Phosphoproteomics Sample Preparation

**DOI:** 10.1021/acs.analchem.3c00324

**Published:** 2023-06-15

**Authors:** Fujia Wang, Tim Veth, Marije Kuipers, Maarten Altelaar, Kelly E. Stecker

**Affiliations:** † Biomolecular Mass Spectrometry and Proteomics, Center for Biomolecular Research and Utrecht Institute for Pharmaceutical Sciences, Utrecht University, Padualaan 8, 3584 CH Utrecht, the Netherlands; ‡ Department of Biomolecular Health Sciences, Faculty of Veterinary Medicine, Utrecht University, Yalelaan 2, 3584 CM Utrecht, the Netherlands

## Abstract

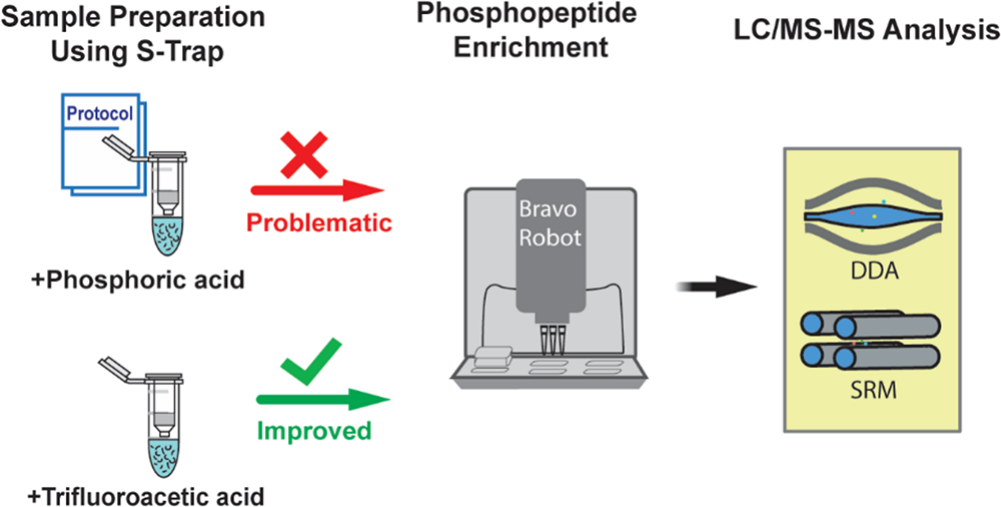

A successful mass spectrometry-based phosphoproteomics analysis relies on effective sample preparation strategies. Suspension trapping (S-Trap) is a novel, rapid, and universal method of sample preparation that is increasingly applied in bottom-up proteomics studies. However, the performance of the S-Trap protocol for phosphoproteomics studies is unclear. In the existing S-Trap protocol, the addition of phosphoric acid (PA) and methanol buffer creates a fine protein suspension to capture proteins on a filter and is a critical step for subsequent protein digestion. Herein, we demonstrate that this addition of PA is detrimental to downstream phosphopeptide enrichment, rendering the standard S-Trap protocol suboptimal for phosphoproteomics. In this study, the performance of the S-Trap digestion for proteomics and phosphoproteomics is systematically evaluated in large-scale and small-scale samples. The results of this comparative analysis show that an optimized S-Trap approach, where trifluoroacetic acid is substituted for PA, is a simple and effective method to prepare samples for phosphoproteomics. Our optimized S-Trap protocol is applied to extracellular vesicles to demonstrate superior sample preparation workflow for low-abundance, membrane-rich samples.

## Introduction

Mass spectrometry (MS)-based analysis of complex proteomes has become a fast, robust, and sensitive method to quantify protein dynamics in biological samples.^1,2^ Technical advancements in liquid chromatography (LC)–MS instrumentation and sample preparation methods have enabled the rapid progression of proteomics applications.^3−7^ Essential to high-quality LC–MS proteome data is the ability to sufficiently lyse lipid-rich biological samples to release proteins for proteolytic digestion. Using anionic detergents such as sodium dodecyl sulfate (SDS) allows for effective solubilization of proteins from membrane-rich samples due to its combined ionic and hydrophobic binding properties. However, the presence of SDS in proteomics samples is detrimental to downstream LC–MS analysis.^8−10^ Traditional proteomics sample cleanup methods, which rely on hydrophobic affinity to capture proteins, are unable to remove detergents such as SDS. As a result, effective detergents are rendered incompatible with standard LC–MS sample preparation workflows.

To resolve the issue of detergent use in LC–MS samples, a method of sample preparation called suspension trapping (S-Trap) was developed.^11^ The S-Trap method extracts proteins based on their denatured size, rather than hydrophobic affinity, thus enabling a detergent-compatible sample preparation method that is fast and effective. By efficiently removing SDS from samples, the S-Trap method prevents downstream interference with enzymatic protein digestion and LC–MS/MS analysis, and it also decreases the time and steps required for MS sample preparation.^11,12^ The S-Trap protocol has been demonstrated to outperform other methods for the preparation of membrane-rich samples such as milk fat globule membranes,^13^ T cell lipid rafts,^14^ mouse brain microglial samples,^15^ and mammalian cell bioreactor supernatants.^16^ Studies comparing different methods for bottom-up proteomics showed that S-Trap is a universal, efficient, and reproducible sample preparation method.^17−21^

For these reasons, the S-Trap method has been gaining popularity and is increasingly applied in bottom-up proteomics studies. However, currently, there is limited use of the method reported in phosphoproteomics studies. In the existing S-Trap protocol, a large amount of phosphoric acid (PA) is added to the sample to create a fine protein suspension. The negative consequence of PA to subsequent phosphopeptide enrichment protocols is unclear and has not been systematically assessed.

Phosphorylation is a ubiquitous protein post-translational modification (PTM) that can alter the protein structure and function and regulates many biological processes in living cells.^22^ MS-based phosphoproteomics is a powerful tool to identify and quantify the phosphorylated proteome of complex biological samples.^7^ Due to the low stoichiometry of phosphorylation within the proteome, a critical element to phosphoproteomics is enriching phosphorylated peptides while preserving phosphopeptide fidelity during sample preparation.^23^ Common enrichment approaches exploit the affinity of the negatively charged phosphate groups of the phosphopeptides toward the positively charged metal ions, such as Fe(III) (immobilized metal affinity chromatography (IMAC)), or metal oxides, such as TiO_2_ (metal oxide affinity chromatography (MOAC)).^24,25^ We hypothesize that the inclusion of PA in the S-Trap protocol may generate problems for the charge-based phosphoenrichment step due to the strong resemblance between phosphate and PA.

In this study, we evaluate the performance of S-Trap, as a sample preparation protocol for phosphoproteomics experiments. We compare the standard S-Trap protocol with modified methods that replace PA with alternate acids and determine the effect on the analysis of both the proteome and phosphoproteome. Our results demonstrate that the standard S-Trap protocol using PA is problematic for phosphopeptide enrichment and negatively affects the detection and quantification of phosphorylated peptides compared to conventional in-solution digestion. We show that replacing PA with trifluoroacetic acid (TFA) can recover S-Trap performance for phosphoproteomics experiments, without influencing proteome measurements for both large-scale (200 μg input) and small-scale (10 μg input) sample preparations. Finally, we apply our optimized protocol to membrane-rich extracellular vesicle (EV)-enriched samples and demonstrate fast and efficient quantification of EV phosphopeptides.

## Materials and Methods

### Sample Preparation

#### SDC-Based In-Solution Sample Preparation

HeLa cell pellets were lysed by the lysis buffer, which consisted of 100 mM Tris, 10 mM TCEP, 40 mM chloroacetamide, a cOmplete mini EDTA-free tablet and PhosSTOP tablet, and 1% (w/v) sodium deoxycholate (SDC) in the purified water. Subsequently, cells were boiled for 5 min at 95 °C and sonicated for 15 min at level 5 (30 s on, 30 s off, Bioruptor, model ACD-200, Diagenode, Liège, Belgium). Clarified cell lysates were measured for protein content using the Pierce BCA Protein Assay Kit (Thermo Scientific, Rockford, IL, United States). The samples were split into three aliquots containing an equal amount of protein. Samples were diluted 1:10 using 50 mM ammonium bicarbonate before trypsin digestion (Sigma-Aldrich, St. Louis, MO, United States) using an enzyme:protein ratio of 1:50 and overnight digestion at 37 °C. The tryptic peptides were acidified with formic acid (FA) at a final concentration of 2%. The peptides were centrifuged at 20,000 × *g*, and then the supernatant was stored for desalting. Peptides were acidified to 5% FA and were desalted using Sep-Pak C18 1 cc Vac cartridges (Waters, Reykjavik, Iceland). Peptides were vacuum-dried and stored at −20 °C before LC–MS analysis.

#### S-Trap Preparation

Sample preparation by the S-Trap method was performed following the S-Trap mini spin column digestion protocol from the manufacturer with slight modification. Cell pellets were washed twice with PBS buffer and subsequently dissolved in 5% SDS and 50 mM TEAB solution. After sonication, samples were reduced with 5 mM TCEP and incubated at 55 °C for 15 min in a thermomixer. The samples were cooled to room temperature and then alkylated with 20 mM chloroacetamide for 10 min. Each sample was split into three aliquots containing equal amounts of protein for parallel S-Trap digestion. The lysate aliquots were acidified with PA (final concentration ∼1.1% PA), and the pH of the samples was confirmed to be ≤1. Subsequently, 350 μL of S-Trap wash buffer (90% methanol/100 mM TEAB, adjust pH with PA to 7.55) was added to the solution. The protein suspension was transferred to the S-Trap mini columns (ProtiFi, NY, United States) and the S-Trap column was centrifuged at 4000 × *g* for 30 s to trap proteins. Washing was repeated four times by adding 400 μL of wash buffer and centrifuged at 4000 × *g* for 30 s. The final column spin was conducted at 4000 × *g* for 1 min to fully remove wash buffer. Next, trypsin (∼1:10 enzyme/protein) in digestion buffer (125 μL of 50 mM TEAB) was added to the surface of the filter and incubated for 1 h at 47 °C. The tryptic peptides were eluted in sequence by 80 μL of elution buffer 1 (50 mM TEAB in water), 80 μL of elution buffer 2 (0.2% FA in water), and 80 μL of elution buffer 3 (50% acetonitrile in water) via centrifugation for 1 min at 4000 × *g*. These three elutions were pooled together and vacuum-centrifuged to dryness and were stored at −80 °C.

For the S-Trap method where PA was replaced with other acids, the same protocol was applied, except for the steps of acidifying the protein solution with acid and the pH adjustment of wash buffer. FA, glycolic acid (GA), and TFA were selected separately to replace PA to acidify lysates. The amounts of acid added in each case were adjusted accordingly to achieve a sample pH of ≤1. Acidification details and final concentrations are shown in the Supporting Information (Table S1). The S-Trap wash buffer (90% methanol/100 mM TEAB) was pH adjusted with each corresponding acid to 7.55.

#### Sample Preparation in Small-Scale Samples and EV Samples

The small-scale digests and EV samples were processed following the same protocols as described above with the following minor modifications: SDC-based in-solution digestion was performed using 60 μL of lysis buffer per cell pellet. The lysate including around 12 μg of proteins was diluted 10-fold using 50 mM ammonium bicarbonate and digested overnight with Trypsin Gold (Promega, Madison, WI, United States) at 37 °C. Peptides were acidified to 5% FA and were desalted using the Oasis PRiME HLB 96-well μElution Plate (Waters, Reykjavik, Iceland). Peptides were vacuum-dried and stored at −20 °C before LC–MS analysis.

Samples were prepared using the manufacturer-supplied S-Trap micro protocol with slight modifications. Three pellets were reduced with TCEP and alkylated by chloroacetamide. Samples were digested using Trypsin Gold (enzyme/protein ∼1:10). For the PA-based S-Trap workflow, the lysate was acidified with PA (final concentration ∼2.5% PA), resulting in a pH of ≤1. For the TFA-based S-Trap workflow, TFA replaced PA as the acidifier for lysates, resulting in a pH of ≤1 (final concentration ∼0.9% TFA), and TFA was used to adjust the pH of the washing buffer.

### Phosphopeptide Enrichment

Phosphorylated peptides were enriched using Fe(III)-NTA 5 μL cartridges on the automated AssayMAP Bravo Platform (Agilent Technologies, Santa Clara, CA, United States), as described previously.^26^ Samples were dissolved in 200 μL of loading buffer (80% acetonitrile/0.1% TFA). Fe(III)-NTA cartridges were primed with 200 μL of 0.1% TFA in acetonitrile and equilibrated with 250 μL of loading buffer. After loading the samples onto the column at a loading speed of 5 μL/min, the column was washed with 250 μL of loading buffer and eluted with 35 μL of 10% ammonia solution into 35 μL of 10% FA. Samples were vacuum-dried and stored at −80 °C. For targeted SRM (selected reaction monitoring) assays, 200 μL of loading buffer was spiked with 100 fmol of stable-isotope-labeled phosphopeptides from the human kinase SpikeMix activation loops (JPT Peptide Technologies, Berlin, Germany).

### LC–MS Analysis

Desalted and dried proteome digests were resuspended in 2% FA, and phosphopeptide-enriched samples were resuspended in 20 mM citric acid/2% FA. Untargeted phosphoproteomics samples were measured using an Orbitrap Exploris 480 mass spectrometer (Thermo Fisher Scientific, San Jose, CA, United States) coupled to an UltiMate 3000 UHPLC system (Thermo Fisher Scientific, San Jose, CA, United States) fitted with a μ-precolumn (C18 PepMap100, 5 μm, 100 Å, 5 mm × 300 μm, Thermo Fisher Scientific, San Jose, CA, United States) and a homemade analytical column (Agilent Poroshell 120 EC-C18, 2.7 μm, 50 cm × 75 μm). Samples were loaded in solvent A (0.1% FA in water) with a flow rate of 30 μL/min and eluted using a 115 min gradient at a flow rate of 300 nL/min. The gradient for peptides was as follows: 9% solvent B (0.1% FA in 80% acetonitrile, 20% water) for 1 min, 9–13% for 1 min, 13–44% for 95 min, 44–99% for 3 min, 99% for 4 min, 99–9% for 1 min, and finally the system equilibrated with 9% B for 10 min. The gradient for phosphopeptides was as follows: 9% solvent B for 1 min, 9–36% for 97 min, 36–99% for 3 min, 99% for 3 min, 99-9% for 1 min, and finally the system equilibrated with 9% B for 10 min. MS data were acquired in data-dependent acquisition (DDA) mode. The electrospray voltage was set at 2000 V, and the ion transfer tube temperature was set to 275 °C. The full scan MS spectra were acquired at a resolution of 60,000 within the *m*/*z* range of 375–1600 using a “Standard” pre-set automated gain control (AGC) target. The RF lens was set to 40%, and the dynamic exclusion time was set to 16 s. In the MS2 setting, high-energy collision dissociation was performed with 28% normalized collision energy at an Orbitrap resolution of 30,000. Multiply charged precursor ions starting from *m*/*z* 120 were selected for further fragmentation. The AGC target was set to standard and a 1.4 *m*/*z* isolation window was used for fragmentation.

### SRM Assay

The targeted phosphoproteomics assay comprising 288 representative stable-isotope-labeled proteotypic phosphorylated peptides for human kinase activation loops was used, as described previously.^27^ Human kinase activation loops were dissolved and mixed with iRT (indexed retention time) peptides for retention time alignment.^28^ In the SRM assay, LC–MS/MS analysis was performed on an UltiMate 3000 RSLCnano system coupled to a TSQ Altis Triple Quadrupole (Thermo Fisher Scientific, San Jose, CA, United States). The enriched peptides were suspended in 20 mM citric acid/2% FA, loaded on a pre-column (C18 PepMap100, 5 μm), and separated on a PepMap RSLC C18 column (2 μm, 75 μm × 25 cm) using a 100 min gradient (2.2 to 29% buffer B 100% ACN + 0.1% FA) at a flow rate of 300 nL/min. The TSQ Altis spray voltage was set at 1.9 kV and fragmented at 1.5 mTorr in the second quadrupole. Retention time windows were set to 5 min, and Q1 and Q3 resolutions were set to 0.7 and 1.2, respectively. The positive polarity and calibrated RF lens was chosen, and a cycle time of 5 s was used. The list of SRM transitions can be found in the Supporting Information.

### Data Processing

All MS files (excluding the targeted phosphoproteomics assay files) were searched using MaxQuant software version 2.0.3.0 (www.maxquant.org).^29^ The MS/MS spectra were searched by the Andromeda search engine against an in silico tryptic digest of *Homo sapiens* proteins from the UniProtKB/Swiss-Prot + TrEMBL sequence database (version July 2021). The parameters of MaxQuant were as follows: cysteine carbamidomethylation as fixed modification, oxidized methionine, protein N-terminal acetylation, and serine/threonine/tyrosine phosphorylation (for the phosphopeptide enrichment data analysis only) as variable modifications; digestion by trypsin, maximum of two missed cleavages. The protein and peptide-spectrum match (PSM) false discovery rate was set to 1%. Label-free quantification (LFQ) was applied for quantification. Processing was conducted without match between runs. Analyses of EV proteins associated with annotated functions were performed according to the DAVID Functional Annotation Tools (david.ncifcrf.gov).^30^ The physiochemical properties of peptides were calculated by in-house scripts. ExoCarta (www.exocarta.org)^31^ was used to compare the identified proteins in EVs.

The targeted phosphoproteomics assays were analyzed using Skyline 21.2.0.565. The quality of the measured peptides was assessed mainly on the signal similarity between the heavy and the light peptides. The chromatographic quality has been assessed by visual inspection of the peak groups. The most important aspects for assessing quality were perfect co-elution, peak shape, and relative contributions of each transition between the heavy and the light peptides. An rdotp of >0.95 was maintained as an indicator of the similarity between the heavy and the light peptides.

## Results and Discussion

### Experimental Design

**Figure 1:**
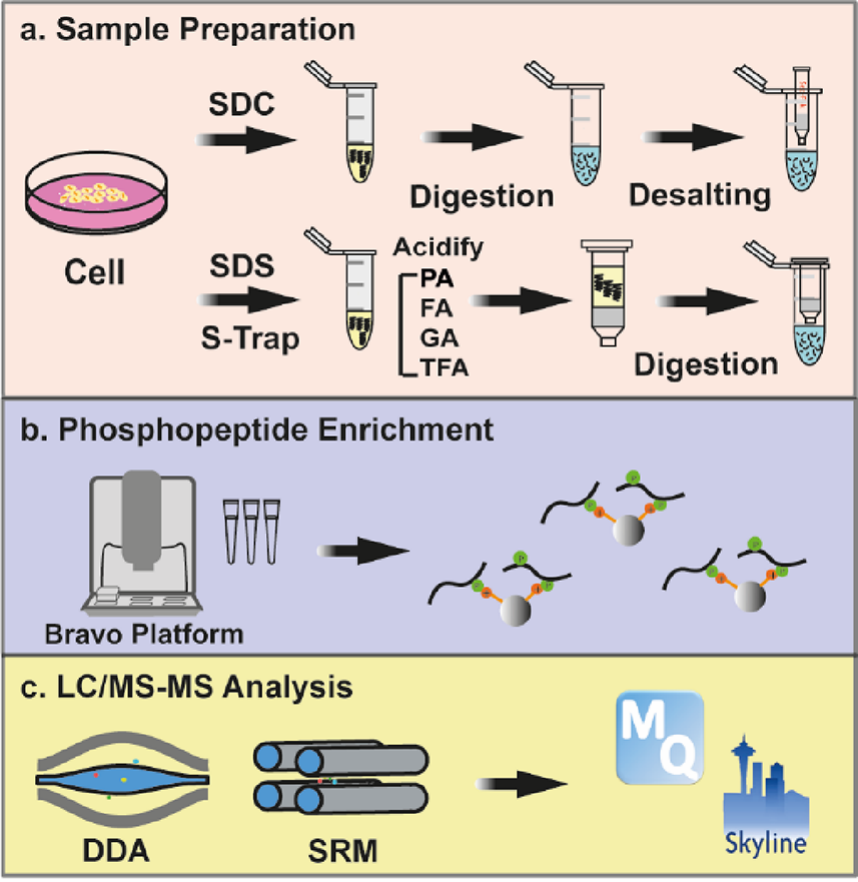
Overview of the experimental design. (a) HeLa cell pellets were aliquoted to five groups with three replicates each. One group was prepared using an in-solution SDC lysis protocol with overnight tryptic digestion, followed by desalting. The remaining four groups were prepared using the S-Trap protocol and acidified using different acids, followed by tryptic digestion on the column for 1 h. (b) Phosphoproteome samples were enriched for phosphopeptides using Fe(III)-NTA cartridges on an Agilent Bravo AssayMAP. Samples were spiked with heavy-isotope-labeled synthetic phosphopeptides prior to enrichment. (c) Proteome samples were analyzed using a DDA method, and phosphoproteome samples were analyzed by DDA and SRM methods.

The aim of this research is to explore the compatibility of S-Trap methods with phosphoproteomics applications to establish a simple, efficient, and universal method for phosphoproteomics sample preparation. To this end, we evaluated five experimental groups with varied sample preparation procedures in our experimental design (Figure 1).

We compared the use of PA, the recommended acidifier in the S-Trap protocol,^18,20^ to three other acids that are commonly used in LC–MS sample preparation: FA, GA, and TFA. As a standard reference, an SDC-based in-solution digestion protocol was performed in parallel.^32^ The S-Trap approach has the benefit of combining digestion and desalting in one step, so only the SDC-based in-solution digests were desalted in an additional step following digestion (Figure 1). In all experimental groups, 200 μg of HeLa lysates was prepared in triplicate and the proteome and phosphoproteome were analyzed using DDA on an Orbitrap Exploris 480. To evaluate the process of phosphopeptide enrichment by an additional metric, heavy-labeled synthetic phosphopeptides were spiked into all samples prior to automated phosphopeptide enrichment, and targeted MS (SRM) analysis was performed to quantify endogenous and spiked-in phosphopeptides across the experimental groups.

### Replacing PA Does Not Affect Proteome Measurements

**Figure 2:**
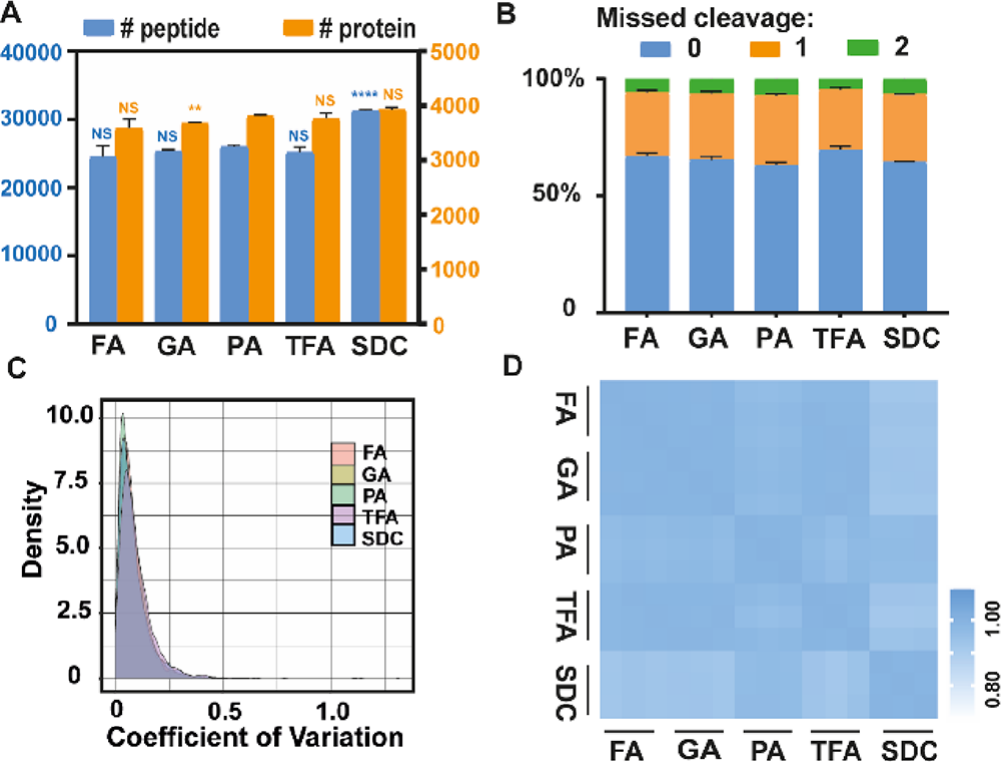
Proteome comparison of different sample preparation methods. (A) Number of proteins and peptides identified by the SDC-based in-solution method and the S-Trap protocol with different acids, including FA, GA, PA, and TFA. NS, no significant differences compared to the PA group; **, significant differences compared to the PA group, *P* < 0.01; ****, significant differences compared to the PA group, *P* < 0.0001. (B) Distribution of missed cleavages per experiment. The percentages of peptides with none, one, or two missed cleavage sites are plotted. (C) Density plot of the coefficient of variation (CV) of the LFQ intensity for the replicates in each experimental group. (D) Pearson correlation coefficient for the log2-transformed LFQ peptide intensities between different experiments.

We first investigated if exchanging PA in the S-Trap procedure affected protein capture, digestion, and quantification in proteome measurements. We found no large difference in the number of peptides and proteins identified across the S-Trap methods (Figure 2A). Furthermore, the reproducibility in identifications between technical replicates was the same for each method (Figure S1A) and highly overlapping across all methods (Figure S1B,C). These findings indicate that replacing the acidifier in the S-Trap protocol has no negative influence on protein capture and proteome detection. To examine the quantitative performance of each group, LFQ was performed and the CV (Figure 2C) and Pearson correlation (Figure 2D) were calculated for each group. Our results showed high technical reproducibility in proteome LFQ measurements across all the methods tested. Next, to assess the influence of acid substitution on protease digestion, we compared the percentage of missed cleavages in each experimental group. The distribution of missed cleavages was similar in all cases with more than 60% of the peptides having no missed cleavages (Figure 2B), suggesting that the replacement of the acidifier from PA with other acids had no influence on digestion performance by trypsin. Finally, to identify if the methods presented a bias in the physicochemical properties of the detected peptides, we compared peptide hydrophobicity (Figure S1D), length (Figure S1E), and the isoelectric point (Figure S1F) for each method. We did not observe any significant differences across these metrics, and the density plots of various physicochemical properties showed highly similar distribution patterns. Taken together, our results demonstrate that replacing PA with TFA, FA, or GA in the S-Trap protocol does not impact protein detection and quantification.

### The S-Trap Protocol Using PA Is Problematic for Phosphopeptide Enrichment and Detection

**Figure 3:**
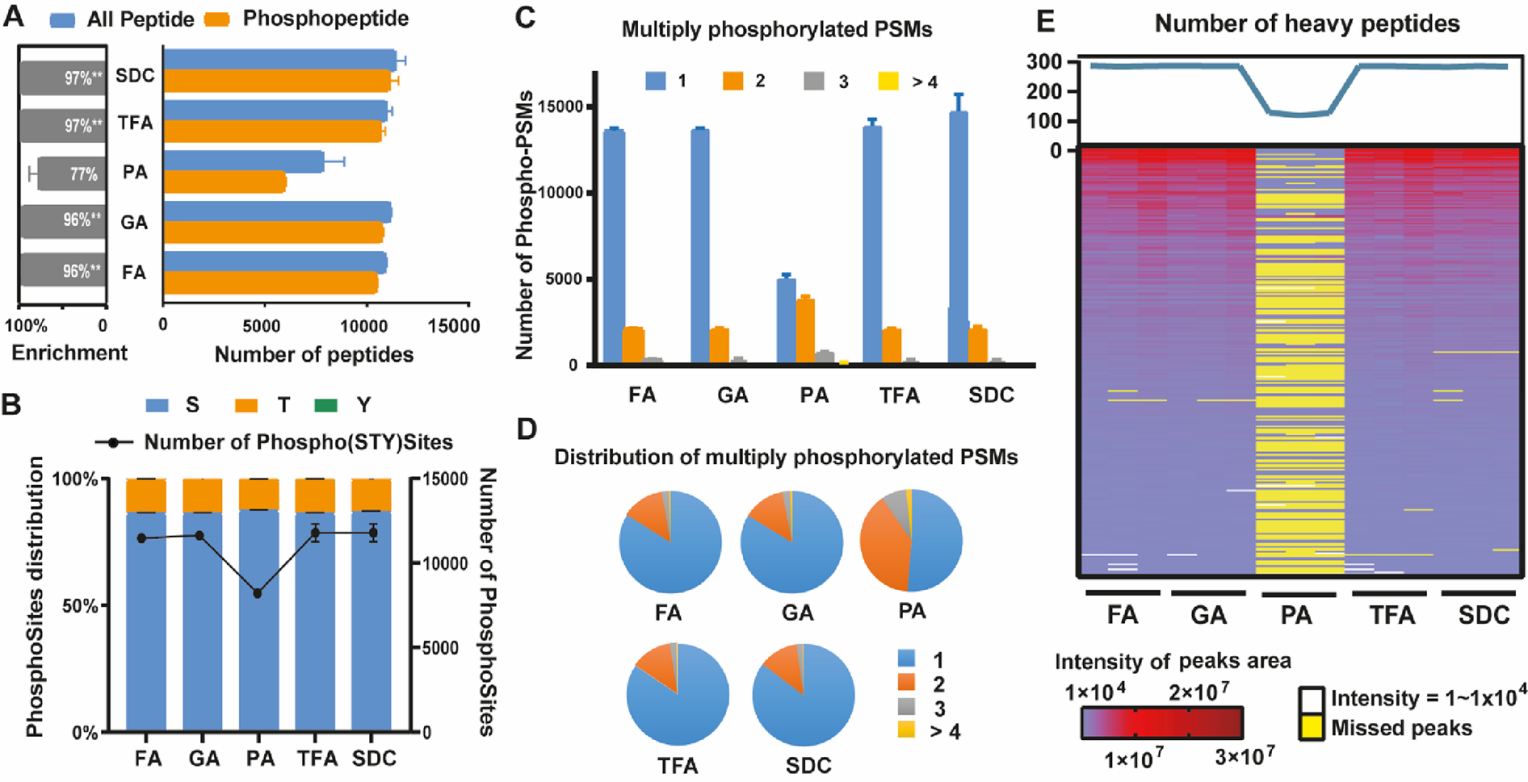
Comparison of phosphoproteome performance using S-Trap with different acids or the SDC protocol. (A) Number of peptides and phosphopeptides detected (right) and the percentage of phosphopeptide enrichment (left) under the different conditions. (B) Total number of identified phosphosites (line plot) and the distribution of serine, threonine, and tyrosine phosphorylation (bar plot) per experiment. (C) Number of singly, doubly, and multiply phosphorylated PSMs. (D) Distribution of phosphosite PSMs measured in Figure 3C. (E) Heatmap of the identified heavy-labeled peptides measured using targeted MS.

We next assessed if PA in the S-Trap protocol was harmful to phosphoproteomics. We compared the LC–MS detection of phosphorylated peptides and PSMs following our IMAC enrichment workflow.^26^ We found that the S-Trap method using PA identified 40% fewer phosphopeptides than the S-Trap method with other acids and caused significantly worse phosphopeptide enrichment (Figure 3A and Figure S2A). Of the phosphosites detected, all experiments yielded the same distribution of phosphorylated amino acids (serine, threonine, or tyrosine) (Figure 3B). However, multiply phosphorylated PSMs (i.e., multiple phosphorylation sites within the same peptide) were distinctly enriched in PA samples (Figure 3C,D). We found that the number of singly phosphorylated peptides in the PA group is much lower than in the other groups, while the number of doubly and multiply phosphorylated peptides was higher (Figure 3C). This data suggests that PA may competitively bind to the cationic surface of the Fe-NTA materials during phosphopeptide enrichment, favoring the stronger binding of multiply phosphorylated peptides and reducing the retention of singly phosphorylated species. In addition to identifications, we assessed the quantitative performance of the different methods. We observed a low Pearson correlation between the PA groups with the other groups, indicating that the use of PA in the S-Trap procedure impacted phosphoproteomics quantification (Figure S2C).

To explore the impact of PA on phosphopeptide enrichment by a second method, we performed a targeted proteomics assay using commercial stable-isotope-labeled phosphopeptide standards.^27^ For each sample, 288 phosphopeptide standards (100 fmol/peptide) were spiked in prior to phosphopeptide enrichment. Less than 40% of the phospho-standards were detected in the PA group, while nearly all were detected and reproducibly quantified in the other groups (Figure 3E). Moreover, the detection of the corresponding endogenous peptides performed dramatically worse in the PA method (Figure S2B). These results clearly demonstrated that employing PA in the S-Trap protocol causes significant problems for phosphopeptide enrichment and quantification and that replacing PA with another acid recovers phosphoproteomics performance using the S-Trap protocol.

### PA Is Problematic for Phosphoproteome Analysis of Small-Scale Sample Preparations

**Figure 4:**
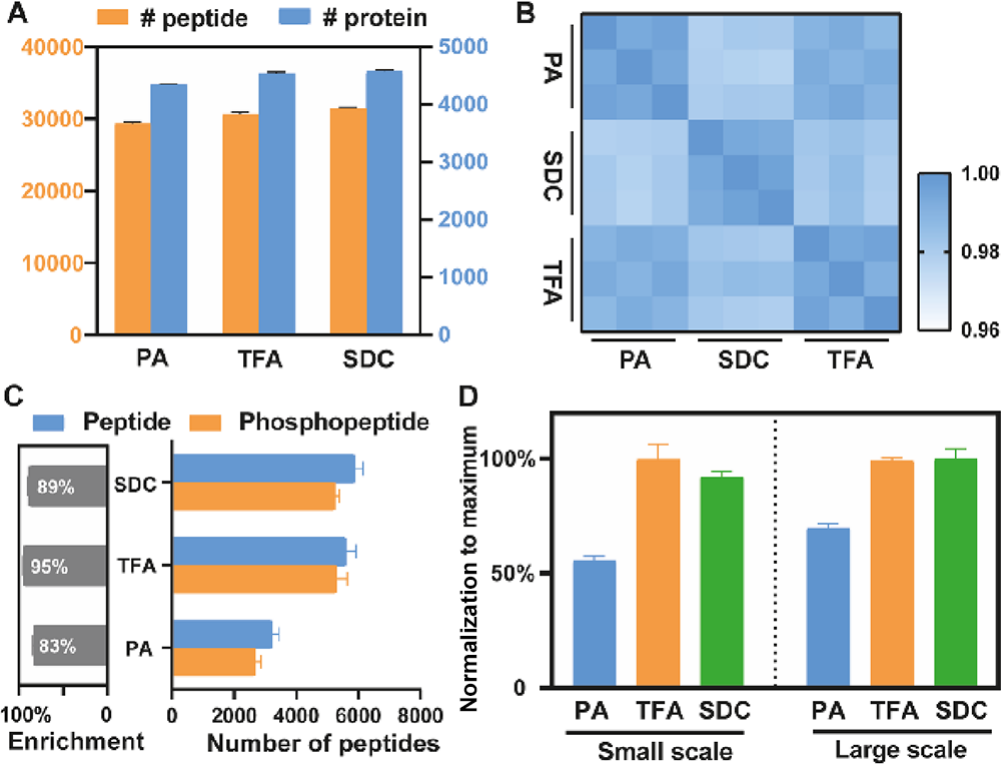
Comparison of the use of PA or TFA in the S-Trap method versus the SDC protocol using small-scale sample amounts (10 μg). (A) Number of peptides and proteins identified in small-scale samples. (B) Pearson correlation coefficient for log2-transformed LFQ peptide intensity values between different experiments. (C) Number of peptides and phosphopeptides detected (right) and the percentage of phosphoenrichment (left). (D) Normalized percentages of identified phosphosites in small-scale (left) and large-scale (right) sample preparations.

Given the poor phosphoproteomics results we observed using the standard S-Trap protocol with 200 μg of protein and the S-Trap mini spin columns, we next wanted to test if the same pattern held true for small-scale protocols using S-Trap micro columns. In this experiment, only 12 μg of protein was digested for each sample and three protocols were compared: the standard PA-based S-Trap method, TFA replacement for PA, and SDC-based in-solution digestion. From each sample, 2 μg was reserved for proteome analysis and the remaining 10 μg was used for phosphopeptide enrichment prior to LC–MS/MS analysis (Figure S3A). Once again, we found no significant difference in proteome measurements for all three methods. The number of identified peptides and proteins was similar (Figure 4A) and the Pearson correlation within and between groups was high (*R* > 0.96), indicating high experimental reproducibility and good proteomics performance in small-scale sample preparations (Figure 4B). Additionally, the distribution of missed cleavages was assessed and no difference was observed between the S-Trap methods with PA or TFA; however, a 10% improvement in digestion efficiency was observed for the SDC samples (Figure S3B). The improvement in SDC in-solution digestion efficiency is likely due to the superior protease quality used for small-scale digestions.

In contrast, the addition of PA in the S-Trap protocol continued to be problematic for small-scale phosphopeptide enrichments. The number of phosphorylated peptides in the PA group was less than in other groups, and the efficiency of phosphopeptide enrichment was lower (Figure 4C). The same trend was also observed in the distribution of phospho(*STY*)sites, phosphorylated PSMs, and the efficiency of phosphopeptide enrichment (Figure S3C,D). The PA enrichments again showed an increase in doubly phosphorylated peptides and reduced numbers of singly phosphorylated peptides (Figure S3E). We directly compared the number of identified STY phosphosites between all experimental conditions and found that the loss in phosphosite detection was even more pronounced in small-scale experiments, with 44% fewer sites measured in the PA sample group compared to TFA (Figure 4D). Taken together, our data demonstrated that the addition of PA in the S-Trap protocol negatively impacts both small-scale and large-scale phosphopeptide enrichments. To overcome this problem, TFA can be used as a replacement PA in the S-Trap protocol.

### Optimized TFA-Based S-Trap Protocol to Study EVs

**Figure 5:**
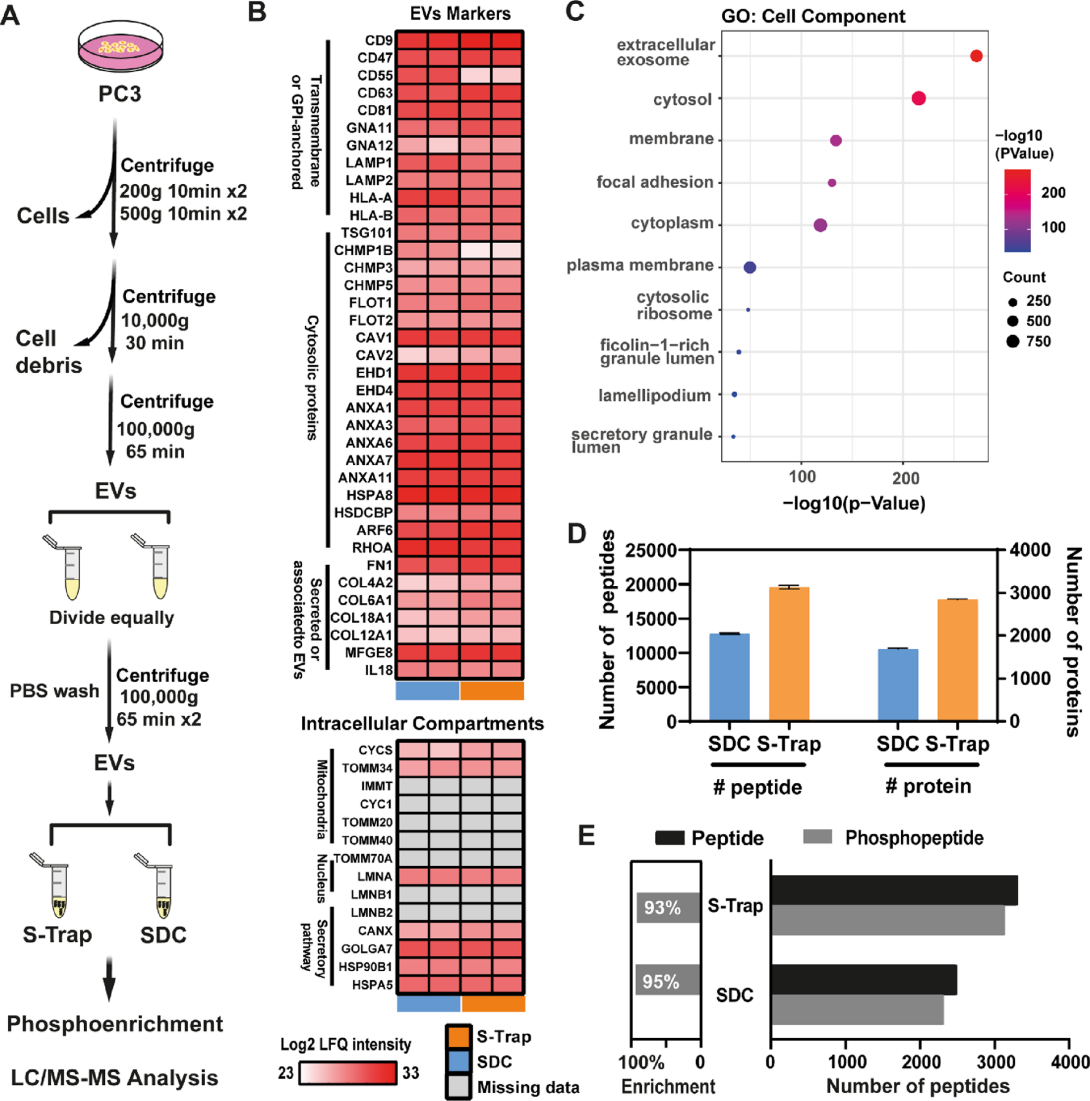
EV characterization. (A) Schematic workflow of EV isolation. (B) Quantitative proteomics analysis of EV lysates (two technical replicates per method). Classical EV markers were enriched and some intracellular compartments were not detected in EV lysates. (C) Top 10 enriched GO terms for proteins observed in samples. (D) Mean number of peptides and proteins identified with the S-Trap or SDC method. Individual data points represent technical replicates for each method. (E) Number of peptides and phosphopeptides identified after phosphopeptide enrichment (right) and the percentage of enrichment (left).

EVs are membranous vesicular particles that are released from cells and carry various DNAs, RNAs, lipids, and proteins. EVs have unique roles in cell–cell communication and can be biomarkers for diseases as well as potential mediators of drug delivery, giving them great clinical utility.^33−36^ Efficient protein extraction from collected EVs can be challenging due to their membrane-rich composition. SDS is an effective chaotropic agent for the extraction of proteins from membrane-rich samples. For this reason, EVs represent an ideal biological sample to apply our optimized S-Trap protocol in which we use SDS to extract proteins and TFA to acidify our samples for proteome and phosphoproteome analysis. We prepared an EV-enriched sample from PC3 prostate cancer cells and split equally into two parts to compare our TFA-based S-Trap digestion method against SDC-based in-solution digestion (Figure 5A). We hypothesized that the S-Trap method would yield greater sample quality due to the use of SDS during protein extraction, a reagent that is poorly compatible with in-solution digestion methods.

Before our method comparison, we first set out to verify the EV enrichment of the sample by characterizing the proteome.^34^ We performed three different assessments: quantification of known EV markers, gene ontology (GO) enrichment, and comparison against known EV databases. In our quantitative comparison, we found that classical EV markers, such as tetraspanins, integrins, TSPANs, and MHC class I proteins, were highly abundant in the proteome, whereas proteins known to be abundant internal compartment markers, such as lamin A/C, mitochondria, IMMT, and cytochrome C (CYC1), were missing in our dataset (Figure 5B).^34^ Second, we examined the subcellular origin of proteins enriched in the EV sample using GO cellular component (GOCC) annotations. In the top 10 GOCC terms, proteins with exosome localization such as extracellular exosome, cytosol, membrane, and focal adhesion were markedly enriched in the samples (Figure 5C). Finally, we compared our generated protein list to an exosome database (ExoCarta) and found that the majority of proteins we identified overlapped with the ExoCarta dataset^31^ (Figure S4). Collectively, our data demonstrate the validity of our EV preparation.

Next, to evaluate the efficiency of the two sample preparation methods for MS analysis of EVs, we compared our proteome and phosphoproteome results. We found that the number of peptides identified in the EV sample was 53% higher using our TFA-based S-Trap method compared to SDC in-solution digest and protein identifications were 68% higher (Figure 5D). The same was true for the phosphoproteome; we identified 826 more phosphopeptides using TFA-S-Trap than with the SDC method (Figure 5E) and both had a high phosphoenrichment efficiency of >92%. This data demonstrates that our optimized TFA-based S-Trap protocol is a fast and effective method for EV sample preparation that yields superior proteome and phosphoproteome coverage.

## Conclusions

The pursuit of robust, efficient, and universal phosphoproteomics sample preparation methods enables the study of phosphorylation dynamics in diverse biological samples. It is important to evaluate the compatibility of widely adopted proteome sample preparation approaches with phosphoproteomics applications. Several protocols exist that enable efficient lysis and enrichment of phosphopeptides, such as EasyPhos.^37^ However, detergent compatibility remains a challenge for most sample preparation methods. In contrast, S-Trap is a fast and universal sample preparation method for MS-based proteome analysis that allows for the use of any detergent, but the utility of the S-Trap method phosphoproteomics is unclear. We speculated that the use of PA in the standard S-Trap protocol may be problematic for phosphopeptide enrichment because of the similar properties between PA and phosphate.

In this study, we demonstrated that the use of PA in the S-Trap protocol is detrimental to phosphopeptide enrichment and results in drastically reduced phosphopeptide identifications. We show that replacing PA with TFA can recover the performance of S-Trap in phosphoproteomics experiments without influencing global proteomics measurements, for both large-scale (200 μg input) and small-scale (10 μg input) sample preparations. We further employed our optimized S-Trap method to membrane-rich EVs and achieved superior protein, peptide, and phosphopeptide coverage compared to an in-solution digestion method using SDC. The dramatic improvement in EV proteome and phosphoproteome coverage we observed is likely due to the advantageous use of SDS for reducing sample loss during EV collection and improving protein extraction during EV lysis. The use of SDS is incompatible with in-solution digest methods, thus demonstrating a unique benefit of the S-Trap approach. The original PA-based S-Trap method in parallel during our EV analysis was not tested due to limited sample materials. However, we anticipate that the benefit of replacing PA for TFA in the S-Trap protocol would provide the same benefit for EVs as was observed in the analysis of 10 μg of cell lysate.

Given the enhanced extraction efficiency of the S-Trap method, this method can be advantageous to various types of biological materials, including formalin-fixed paraffin-embedded (FFPE) samples and transmembrane proteins.^38,39^ Moreover, the optimized S-Trap methodology may be used as a vital part of a quick workflow to investigate molecular signaling in cancer cells for clinical proteomics.^40^ Looking forward, the S-Trap method could facilitate the preparation for other types of PTMs, including glycosylation and methylation. Certainly, evaluation and optimization before application could be necessary.

## Data Availability

The mass spectrometry proteomics data have been deposited to the ProteomeXchange Consortium via the PRIDE^41,42^ partner repository with the dataset identifier PXD037751.

## Supporting Information Available

Additional experimental details, including reagents and cell culture and isolation of EVs; (Figures S1–S4) comparison of the overlap and physicochemical properties of different sample preparation methods, identified and quantitative phosphoproteomics comparison of different sample preparation methods, comparison of the performance in small-scale samples, and Venn diagrams of the overlap in identified proteins with the ExoCarta exosome database; (Table S1) details of acidifiers added in the S-Trap method; and (Table S2) SRM transitions list used for data acquisition (PDF)
