# Supplementary material for: Optimized Suspension Trapping Method for Phosphoproteomics Sample Preparation
Source: Anal Chem. 2023 Jun 15;95(25):9471–9. doi: 10.1021/acs.analchem.3c00324 (PMC10308333; doi:10.1021/acs.analchem.3c00324)
Supplement: Supplementary file 1 — ac3c00324_si_001.pdf [file ac3c00324_si_001.pdf]

Supporting Information

Optimized Suspension Trapping Method for Phosphoproteomics Sample Preparation

Fujia Wang[1], Tim Veth[1], Marije Kuipers[2], Maarten Altelaar[1] and Kelly E. Stecker[1*]

1. Biomolecular Mass Spectrometry and Proteomics, Center for Biomolecular Research and Utrecht Institute for Pharmaceutical Sciences, Utrecht University, Padualaan 8, 3584 CH, Utrecht, the Netherlands

2. Department of Biomolecular Health Sciences, Faculty of Veterinary Medicine, Utrecht University, Yalelaan 2, 3584 CM, Utrecht, the Netherlands

*Corresponding Author: Kelly E. Stecker (E-mail address: k.e.stecker@uu.nl)

Table of Contents

Supplementary Materials and Methods S3
Reagents S3
Cell Culture and Isolation of EVs S3
Acidification in the S-Trap Method S3
Supplementary Figures S5
Figure S1 .... S5
Figure S2 .... S6
Figure S3 .... S7
Figure S4 .... S8
Supplementary Tables S9
Table S1 .... S9
Table S2 .... S10

Supplementary Materials and Methods

Reagents

Sodium dodecyl sulfate (SDS), sodium deoxycholate (SDC), triethylammonium bicarbonate (TEAB), chloroacetamide, tris(2-carboxyethyl)phosphine (TCEP), tris(hydroxymethyl) aminomethane (Tris), ammonium bicarbonate (AMBIC), and trypsin were purchased from Sigma-Aldrich (Darmstadt, Germany). Ammonia solution, formic acid (FA) and glycolic acid (GA) were purchased from Merck (Darmstadt, Germany). Other resources included cOmplete mini tablet (Roche, Mannheim, Germany), PhosSTOP tablets (Roche, Mannheim, Germany), Oasis PRiME HLB 96-well µElution Plate (Waters, Etten-Leur, The Netherlands), Ultrapure MilliQ water was prepared with the system (Merck Millipore, Darmstadt, Germany), trifluoroacetic acid (Honeywell, Charlotte, NC, United States), phosphoric acid (Acros, Germering, Germany), human kinase activation loops-heavy (JPT, Berlin, Germany) and Trypsin Gold of mass spectrometry grade (Promega, Madison, United States).

Cell Culture and Isolation of EVs

Hela cells and PC3 cells were purchased from ATCC. Hela cells were cultured in standard Roswell Park Memorial Institute (RPMI) medium 1640 medium (Lonza, Basel, Switzerland), containing 10% FBS (Thermo Scientific, Rockford, IL, United States), 2 mM L-glutamine and 1% penicillin/streptomycin (Lonza, Basel, Switzerland), at 37°C in a humidified atmosphere containing 5% CO_2. Cells were detached from the culture surface using trypsin (Lonza, Basel, Switzerland) and washed three times with PBS before lysis.

PC3 cells were cultured in Gibco RPMI 1640 glutamax (Thermo Fisher Scientific, Germering, Germany) supplemented with 10% FBS (GE Healthcare, Chicago, IL, USA) and penicillin/streptomycin (Gibco) in T175 flasks (Corning, NY, United States) till 85% confluent. The medium was subsequently removed and replaced by 30 mL / flask RPMI without FBS for overnight for EV collection. The collected culture medium was centrifuged twice for 10 min at 200 × g and twice for 10 min at 500 × g, all for 10 minutes and at 4°C, where the supernatant was decanted into new tubes after each centrifugation step. The final 500 × g supernatant was stored at -80 °C till further use. 210 mL of thawed 500 × g culture supernatant was centrifuged in polypropylene tubes (Beckman Coulter, Brea, CA, United States) at 10,000 × g for 30 minutes at 4°C (SW32 Ti rotor, 8,900 rpm). The 100,000 × g supernatant was transferred to new SW32 tubes and centrifuged at 100,000 × g for 65 min at 4°C (28,000 rpm). The supernatant was removed until the conical part of the tube and the pellets were resuspended in the remaining supernatant and transferred and split equally into two SW40 tubes. Tubes were topped up with the collected 100,000 × g supernatant and centrifuged in an SW40 Ti rotor at 100,000 × g (28,000 rpm) for 65 min at 4 °C. After removal of the supernatant till the conical part of the tube, the pellets were resuspended and topped up with 11 mL PBS for a washing step in the SW40 rotor with same speed and time. Finally, the supernatant was removed until the conical part of the tube, the tube was decanted, and the tube walls were dried with a tissue. The final EV-enriched pellets were resuspended in 20 µL PBS or 20 µL SDS buffer (5%) and stored at -80°C till sample preparation.

Acidification in the S-Trap Method

In the acidification step, the final concentration of acids depended on the amounts of acids added which led to the pH of the samples being confirmed to be ≤ 1. In the test, we decided on the amounts of acids according to the amounts of acid added can make the pH of the samples to be ≤ 1. Respectively, as shown in the **Table S1**, according to the standard S-Trap protocol, we added 2.5 µL of 27.5% PA to 25 µL of sample for the S-Trap micro column (final concentration ~2.5%) and 5 µL of 12% PA to 50 µL of sample for the S-Trap mini column (final concentration ~1.1%). For all of the other acids, we tested the amount of acids required

to achieve sample acidification (pH ≤ 1). Accordingly, we added 2.5 µL of 10% TFA to 25 µL of sample for the S-Trap micro column (final concentration ~0.9%) and 5 µL of 10% TFA to 50 µL of sample for the S-Trap mini column (final concentration ~0.9%). We added 5 µL of 100% FA to 25 µL of sample for the S-Trap micro column (final concentration ~16.7%) and added 10 µL of 100% FA to 50 µL of sample for the S-Trap mini column (final concentration ~16.7%). We added 15 µL of 70% GA to 25 µL of sample for the S-Trap micro column (final concentration ~26.3%) and add 30 µL of 70% GA to 50 µL of sample for the S-Trap mini column (final concentration ~26.3%). In the practical, the pH of acidified sample will be ≤ 1. If the sample pH is not ≤ 1, additional amounts of acid should be added to reach pH ≤ 1.

Supplementary Figures

A

Identified overlap in replicates

Group	Peptide		Protein	
PA	20652	66%	3421	82%
TFA	19734	64%	3349	81%
FA	18822	63%	3153	79%
GA	20133	66%	3290	81%
SDC	25050	67%	3508	81%

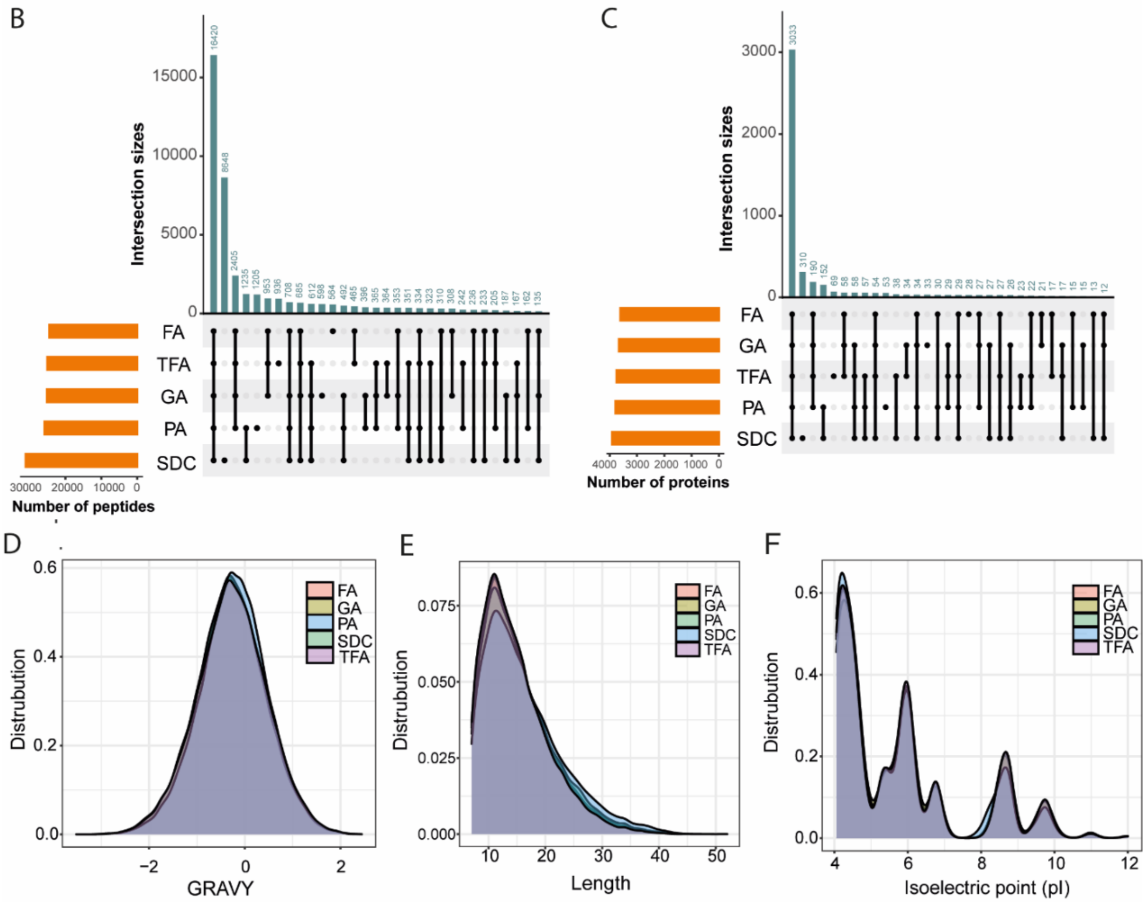

Figure S1. Comparing the overlap and physicochemical properties of different sample preparation methods. (A) The number of peptides and proteins identified from all three replicates and their respective percentages of identification versus the whole dataset per group. The UpSetR plot depicting the intersection between the peptides identified (B) and proteins identified (C) in at least 2/3 replicates per sample preparation method. Horizontal bars display the total number of peptides or proteins detected by each method; vertical bars display the number of jointly identified peptides or proteins. The density plot of identified peptides by the hydrophobicity (GRAVY value, D), the length of peptides (E), and the isoelectric point (pI, F).

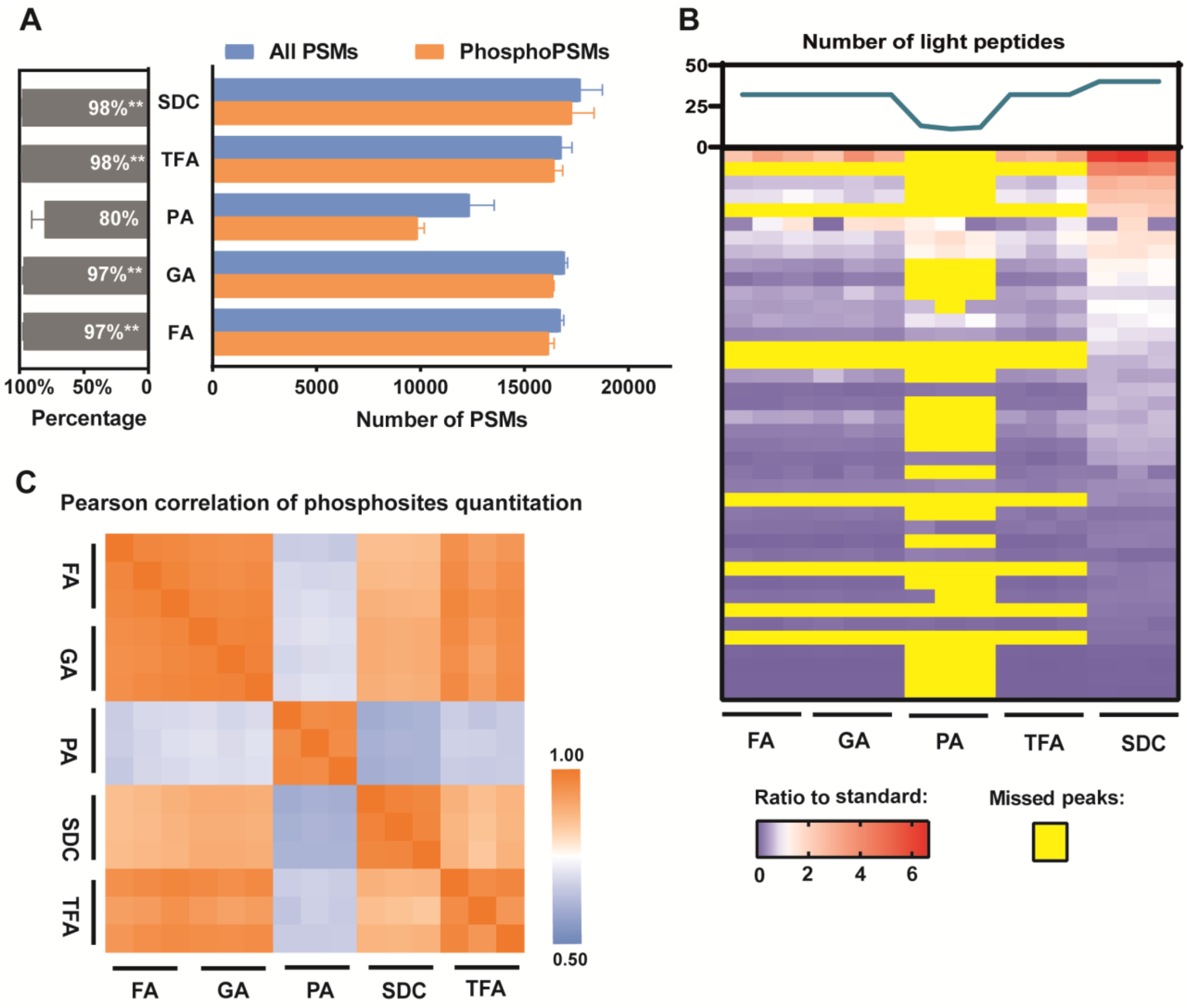

Figure S2. Identified and quantitative phosphoproteomics comparison of different sample preparation methods. (A) The number of PSMs and PSMs of phosphorylated peptides (right), and the percentage of phosphorylated PSMs enrichment (left) in different groups. (B) The number of endogenous peptides detected and quantified corresponds to the heavy-labeled spike-ins. Peptides are presented using a light/heavy phosphopeptide ratio. (C) Pearson correlation coefficient for log2-transformed LFQ phosphopeptide intensity values between different sample preparation methods.

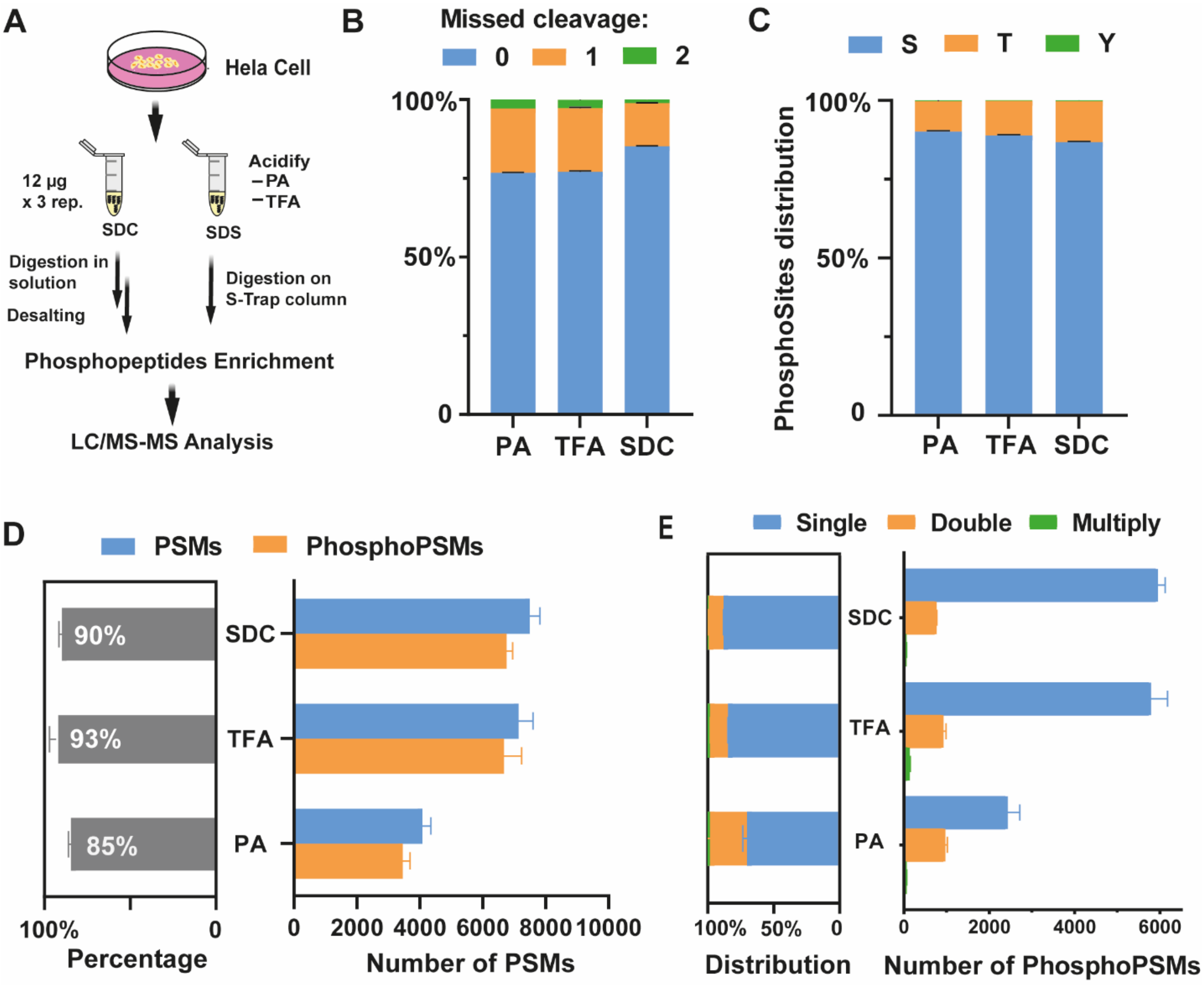

Figure S3. The comparison of performance in small scale samples. (A) Schematic workflow of the experiment design in small-scale samples (10μg). (B) Distribution of missed tryptic cleavage sites by each group. The percentage of peptides with no missed cleavage, one missed cleavage site, and two missed cleavage sites were plotted. (C) The distribution of serine, threonine, and tyrosine phosphorylation. (D) The number of peptide-spectrum matches (PSMs) and phosphorylated PSMs (right), and the percentage of phosphorylated PSMs enrichment (left) in different groups. (E) Distribution of phosphorylated residues in the phosphopeptides (left) and the number of single, double, and multiply phosphorylated peptides (right) in different methods.

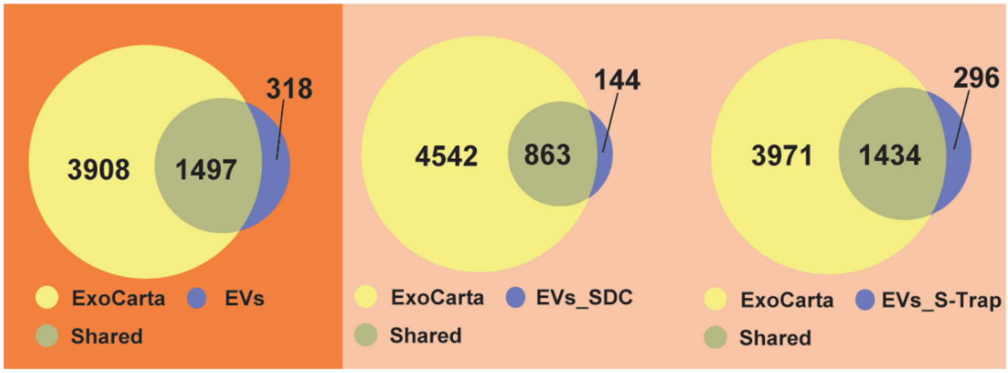

Figure S4. Venn diagrams of the overlap in identified proteins (from all samples, from the SDC method, and from the S-Trap method) with the ExoCarta exosome database.

Supplementary Tables

Table S1. The details of acidifiers added in S-Trap method

	S-Trap Micro column			S-Trap Mini column		
**Acidifier**	**Concentration**	**Volume**	**Final Conc.**	**Concentration**	**Volume**	**Final Conc.**
Phosphoric acid	27.5%	2.5 µL	2.5%	12%	5 µL	1.1%
Glycolic acid	70%	15 µL	26.3%	70%	30 µL	26.3%
Formic acid	100%	5 µL	16.7%	100%	10 µL	16.7%
Trifluoroacetic acid	10%	2.5 µL	0.9%	10%	5 µL	0.9%

Table S2. SRM transitions list used for data acquisition.

Compound	Start Time (min)	End Time (min)	Precursor (m/z)	Product (m/z)	Collision Energy (V)
AENGLLM[+15.994915]T[+79.966331]PC[+57.021464]YTANFVAPEVLK(+3)	86.67	91.67	845.395	755.466166	25.4
AENGLLM[+15.994915]T[+79.966331]PC[+57.021464]YTANFVAPEVLK(+3)	86.67	91.67	845.395	656.397753	26.4
AENGLLM[+15.994915]T[+79.966331]PC[+57.021464]YTANFVAPEVLK(+3)	86.67	91.67	845.395	585.360639	33.4
AENGLLM[+15.994915]T[+79.966331]PC[+57.021464]YTANFVAPEVLK (heavy)(+3)	86.67	91.67	848.066	763.480365	25.4
AENGLLM[+15.994915]T[+79.966331]PC[+57.021464]YTANFVAPEVLK (heavy)(+3)	86.67	91.67	848.066	664.411952	26.4
AENGLLM[+15.994915]T[+79.966331]PC[+57.021464]YTANFVAPEVLK (heavy)(+3)	86.67	91.67	848.066	593.374838	33.4
AENGLLMT[+79.966331]PC[+57.021464]YTANFVAPEVLK(+3)	92.55	97.55	840.063	755.466166	21.2
AENGLLMT[+79.966331]PC[+57.021464]YTANFVAPEVLK(+3)	92.55	97.55	840.063	656.397753	22.2
AENGLLMT[+79.966331]PC[+57.021464]YTANFVAPEVLK(+3)	92.55	97.55	840.063	585.360639	34.2
AENGLLMT[+79.966331]PC[+57.021464]YTANFVAPEVLK (heavy)(+3)	92.55	97.55	842.734	763.480365	21.2
AENGLLMT[+79.966331]PC[+57.021464]YTANFVAPEVLK (heavy)(+3)	92.55	97.55	842.734	664.411952	22.2
AENGLLMT[+79.966331]PC[+57.021464]YTANFVAPEVLK (heavy)(+3)	92.55	97.55	842.734	593.374838	34.2
AFS[+79.966331]LAK(+2)	39.25	44.25	358.672	547.323859	12.7
AFS[+79.966331]LAK(+2)	39.25	44.25	358.672	400.255445	13.7
AFS[+79.966331]LAK(+2)	39.25	44.25	358.672	218.149918	17.7
AFS[+79.966331]LAK (heavy)(+2)	39.25	44.25	362.679	555.338058	12.7
AFS[+79.966331]LAK (heavy)(+2)	39.25	44.25	362.679	408.269644	13.7
AFS[+79.966331]LAK (heavy)(+2)	39.25	44.25	362.679	226.164117	17.7
ALGADDSY[+79.966331]YTAR(+2)	39.48	44.48	691.784	1070.382647	22.7
ALGADDSY[+79.966331]YTAR(+2)	39.48	44.48	691.784	955.355704	24.7
ALGADDSY[+79.966331]YTAR(+2)	39.48	44.48	691.784	242.149918	28.7
ALGADDSY[+79.966331]YTAR (heavy)(+2)	39.48	44.48	696.788	1080.390916	22.7
ALGADDSY[+79.966331]YTAR (heavy)(+2)	39.48	44.48	696.788	965.363973	24.7
ALGADDSY[+79.966331]YTAR (heavy)(+2)	39.48	44.48	696.788	242.149918	28.7
ANS[+79.966331]FVGTAQYVSPELLTEK(+3)	77	82	712.008	1015.567003	21.3
ANS[+79.966331]FVGTAQYVSPELLTEK(+3)	77	82	712.008	916.498589	21.3
ANS[+79.966331]FVGTAQYVSPELLTEK(+3)	77	82	712.008	829.46656	21.3
ANS[+79.966331]FVGTAQYVSPELLTEK (heavy)(+3)	77	82	714.679	1023.581202	21.3
ANS[+79.966331]FVGTAQYVSPELLTEK (heavy)(+3)	77	82	714.679	924.512788	21.3
ANS[+79.966331]FVGTAQYVSPELLTEK (heavy)(+3)	77	82	714.679	837.480759	21.3
APEMVNLY[+79.966331]SGK(+2)	45.72	50.72	644.785	991.431846	22.2
APEMVNLY[+79.966331]SGK(+2)	45.72	50.72	644.785	860.391361	23.2
APEMVNLY[+79.966331]SGK(+2)	45.72	50.72	644.785	761.322947	24.2
APEMVNLY[+79.966331]SGK (heavy)(+2)	45.72	50.72	648.792	999.446045	22.2
APEMVNLY[+79.966331]SGK (heavy)(+2)	45.72	50.72	648.792	868.40556	23.2
APEMVNLY[+79.966331]SGK (heavy)(+2)	45.72	50.72	648.792	769.337146	24.2
APEM[+15.994915]VNLYS[+79.966331]GK(+2)	40.91	45.91	652.783	762.414465	26.5
APEM[+15.994915]VNLYS[+79.966331]GK(+2)	40.91	45.91	652.783	663.346051	28.5

APEM[+15.994915]VNLYS[+79.966331]GK(+2)	40.91	45.91	652.783	436.219059	28.5
APEM[+15.994915]VNLYS[+79.966331]GK (heavy)(+2)	40.91	45.91	656.79	770.428664	26.5
APEM[+15.994915]VNLYS[+79.966331]GK (heavy)(+2)	40.91	45.91	656.79	671.36025	28.5
APEM[+15.994915]VNLYS[+79.966331]GK (heavy)(+2)	40.91	45.91	656.79	444.233258	28.5
APEM[+15.994915]VNLY[+79.966331]SGK(+2)	34	39	652.783	761.322947	25.5
APEM[+15.994915]VNLY[+79.966331]SGK(+2)	34	39	652.783	534.195955	27.5
APEM[+15.994915]VNLY[+79.966331]SGK(+2)	34	39	652.783	617.264697	23.5
APEM[+15.994915]VNLY[+79.966331]SGK (heavy)(+2)	34	39	656.79	769.337146	25.5
APEM[+15.994915]VNLY[+79.966331]SGK (heavy)(+2)	34	39	656.79	542.210154	27.5
APEM[+15.994915]VNLY[+79.966331]SGK (heavy)(+2)	34	39	656.79	621.271796	23.5
APEMVNLYS[+79.966331]GK(+2)	53.04	58.04	644.785	436.219059	28.2
APEMVNLYS[+79.966331]GK(+2)	53.04	58.04	644.785	560.740857	24.2
APEMVNLYS[+79.966331]GK(+2)	53.04	58.04	644.785	298.139747	26.2
APEMVNLYS[+79.966331]GK (heavy)(+2)	53.04	58.04	648.792	444.233258	28.2
APEMVNLYS[+79.966331]GK (heavy)(+2)	53.04	58.04	648.792	564.747957	24.2
APEMVNLYS[+79.966331]GK (heavy)(+2)	53.04	58.04	648.792	298.139747	26.2
APEMVNLY[+79.966331]S[+79.966331]GK(+2)	54.13	59.13	684.768	743.312382	27.4
APEMVNLY[+79.966331]S[+79.966331]GK(+2)	54.13	59.13	684.768	516.18539	32.4
APEMVNLY[+79.966331]S[+79.966331]GK(+2)	54.13	59.13	684.768	298.139747	30.4
APEMVNLY[+79.966331]S[+79.966331]GK (heavy)(+2)	54.13	59.13	688.776	751.326581	27.4
APEMVNLY[+79.966331]S[+79.966331]GK (heavy)(+2)	54.13	59.13	688.776	524.199589	32.4
APEMVNLY[+79.966331]S[+79.966331]GK (heavy)(+2)	54.13	59.13	688.776	298.139747	30.4
APEM[+15.994915]VNLY[+79.966331]S[+79.966331]GK(+2)	40.41	45.41	692.766	743.312382	27.7
APEM[+15.994915]VNLY[+79.966331]S[+79.966331]GK(+2)	40.41	45.41	692.766	629.269455	27.7
APEM[+15.994915]VNLY[+79.966331]S[+79.966331]GK(+2)	40.41	45.41	692.766	516.18539	30.7
APEM[+15.994915]VNLY[+79.966331]S[+79.966331]GK (heavy)(+2)	40.41	45.41	696.773	751.326581	27.7
APEM[+15.994915]VNLY[+79.966331]S[+79.966331]GK (heavy)(+2)	40.41	45.41	696.773	637.283654	27.7
APEM[+15.994915]VNLY[+79.966331]S[+79.966331]GK (heavy)(+2)	40.41	45.41	696.773	524.199589	30.7
ATDS[+79.966331]FSGR(+2)	21.27	26.27	460.679	650.289264	18.7
ATDS[+79.966331]FSGR(+2)	21.27	26.27	460.679	535.262321	21.7
ATDS[+79.966331]FSGR(+2)	21.27	26.27	460.679	319.172444	21.7
ATDS[+79.966331]FSGR (heavy)(+2)	21.27	26.27	465.683	660.297533	18.7
ATDS[+79.966331]FSGR (heavy)(+2)	21.27	26.27	465.683	545.27059	21.7
ATDS[+79.966331]FSGR (heavy)(+2)	21.27	26.27	465.683	329.180713	21.7
AVPEGHEY[+79.966331]YR(+2)	23.2	28.2	650.771	904.334909	28.4
AVPEGHEY[+79.966331]YR(+2)	23.2	28.2	650.771	338.18228	31.4
AVPEGHEY[+79.966331]YR(+2)	23.2	28.2	650.771	452.671092	27.4
AVPEGHEY[+79.966331]YR (heavy)(+2)	23.2	28.2	655.775	914.343178	28.4
AVPEGHEY[+79.966331]YR (heavy)(+2)	23.2	28.2	655.775	348.190549	31.4
AVPEGHEY[+79.966331]YR (heavy)(+2)	23.2	28.2	655.775	457.675227	27.4
AY[+79.966331]TPVVVTLWYR(+2)	85.84	90.84	774.386	837.46175	25.1

AY[+79.966331]TPVVVTLWYR(+2)	85.84	90.84	774.386	315.074049	33.1
AY[+79.966331]TPVVVTLWYR(+2)	85.84	90.84	774.386	416.121728	25.1
AY[+79.966331]TPVVVTLWYR (heavy)(+2)	85.84	90.84	779.39	847.470019	25.1
AY[+79.966331]TPVVVTLWYR (heavy)(+2)	85.84	90.84	779.39	315.074049	33.1
AY[+79.966331]TPVVVTLWYR (heavy)(+2)	85.84	90.84	779.39	416.121728	25.1
AYT[+79.966331]PVVVTLWYR(+2)	93.84	98.84	774.386	1132.651342	25.1
AYT[+79.966331]PVVVTLWYR(+2)	93.84	98.84	774.386	936.530164	29.1
AYT[+79.966331]PVVVTLWYR(+2)	93.84	98.84	774.386	235.107718	30.1
AYT[+79.966331]PVVVTLWYR (heavy)(+2)	93.84	98.84	779.39	1142.659611	25.1
AYT[+79.966331]PVVVTLWYR (heavy)(+2)	93.84	98.84	779.39	946.538433	29.1
AYT[+79.966331]PVVVTLWYR (heavy)(+2)	93.84	98.84	779.39	235.107718	30.1
AY[+79.966331]T[+79.966331]PVVVTLWYR(+2)	105.59	110.59	814.369	936.530164	23.3
AY[+79.966331]T[+79.966331]PVVVTLWYR(+2)	105.59	110.59	814.369	837.46175	26.3
AY[+79.966331]T[+79.966331]PVVVTLWYR(+2)	105.59	110.59	814.369	398.111163	21.3
AY[+79.966331]T[+79.966331]PVVVTLWYR (heavy)(+2)	105.59	110.59	819.373	946.538433	23.3
AY[+79.966331]T[+79.966331]PVVVTLWYR (heavy)(+2)	105.59	110.59	819.373	847.470019	26.3
AY[+79.966331]T[+79.966331]PVVVTLWYR (heavy)(+2)	105.59	110.59	819.373	398.111163	21.3
AYS[+79.966331]FC[+57.021464]GTIEYM[+15.994915]APDIVR(+2)	79	84	1044.943	980.486979	34.3
AYS[+79.966331]FC[+57.021464]GTIEYM[+15.994915]APDIVR(+2)	79	84	1044.943	599.351137	34.3
AYS[+79.966331]FC[+57.021464]GTIEYM[+15.994915]APDIVR(+2)	79	84	1044.943	387.27143	38.3
AYS[+79.966331]FC[+57.021464]GTIEYM[+15.994915]APDIVR (heavy)(+2)	79	84	1049.948	990.495248	34.3
AYS[+79.966331]FC[+57.021464]GTIEYM[+15.994915]APDIVR (heavy)(+2)	79	84	1049.948	609.359406	34.3
AYS[+79.966331]FC[+57.021464]GTIEYM[+15.994915]APDIVR (heavy)(+2)	79	84	1049.948	397.279699	38.3
AYS[+79.966331]FC[+57.021464]GTIEYMAPDIVR(+2)	90.86	95.86	1036.946	1093.534657	35
AYS[+79.966331]FC[+57.021464]GTIEYMAPDIVR(+2)	90.86	95.86	1036.946	670.38825	31
AYS[+79.966331]FC[+57.021464]GTIEYMAPDIVR(+2)	90.86	95.86	1036.946	599.351137	34
AYS[+79.966331]FC[+57.021464]GTIEYMAPDIVR (heavy)(+2)	90.86	95.86	1041.95	1103.542926	35
AYS[+79.966331]FC[+57.021464]GTIEYMAPDIVR (heavy)(+2)	90.86	95.86	1041.95	680.396519	31
AYS[+79.966331]FC[+57.021464]GTIEYMAPDIVR (heavy)(+2)	90.86	95.86	1041.95	609.359406	34
DDEY[+79.966331]NPC[+57.021464]QGSK(+2)	20.02	25.02	696.742	676.308286	23.8
DDEY[+79.966331]NPC[+57.021464]QGSK(+2)	20.02	25.02	696.742	291.166296	24.8
DDEY[+79.966331]NPC[+57.021464]QGSK(+2)	20.02	25.02	696.742	360.103755	24.8
DDEY[+79.966331]NPC[+57.021464]QGSK (heavy)(+2)	20.02	25.02	700.749	684.322485	23.8
DDEY[+79.966331]NPC[+57.021464]QGSK (heavy)(+2)	20.02	25.02	700.749	299.180495	24.8
DDEY[+79.966331]NPC[+57.021464]QGSK (heavy)(+2)	20.02	25.02	700.749	360.103755	24.8
DDIY[+79.966331]SPSSSSK(+2)	23.58	28.58	633.25	679.32571	20.9
DDIY[+79.966331]SPSSSSK(+2)	23.58	28.58	633.25	592.293681	21.9
DDIY[+79.966331]SPSSSSK(+2)	23.58	28.58	633.25	587.174886	18.9
DDIY[+79.966331]SPSSSSK (heavy)(+2)	23.58	28.58	637.257	687.339909	20.9
DDIY[+79.966331]SPSSSSK (heavy)(+2)	23.58	28.58	637.257	600.30788	21.9
DDIY[+79.966331]SPSSSSK (heavy)(+2)	23.58	28.58	637.257	587.174886	18.9

DIMHDSNY[+79.966331]VSK(+2)	34.41	39.41	694.781	576.242906	25.7
DIMHDSNY[+79.966331]VSK(+2)	34.41	39.41	694.781	234.144832	29.7
DIMHDSNY[+79.966331]VSK(+2)	34.41	39.41	694.781	515.205496	27.7
DIMHDSNY[+79.966331]VSK (heavy)(+2)	34.41	39.41	698.788	584.257105	25.7
DIMHDSNY[+79.966331]VSK (heavy)(+2)	34.41	39.41	698.788	242.159031	29.7
DIMHDSNY[+79.966331]VSK (heavy)(+2)	34.41	39.41	698.788	519.212596	27.7
DIM[+15.994915]HDSNY[+79.966331]VSK(+2)	23.09	28.09	702.778	777.317862	26
DIM[+15.994915]HDSNY[+79.966331]VSK(+2)	23.09	28.09	702.778	690.285833	21
DIM[+15.994915]HDSNY[+79.966331]VSK(+2)	23.09	28.09	702.778	234.144832	29
DIM[+15.994915]HDSNY[+79.966331]VSK (heavy)(+2)	23.09	28.09	706.785	785.332061	26
DIM[+15.994915]HDSNY[+79.966331]VSK (heavy)(+2)	23.09	28.09	706.785	698.300032	21
DIM[+15.994915]HDSNY[+79.966331]VSK (heavy)(+2)	23.09	28.09	706.785	242.159031	29
DIHHIDYY[+79.966331]K(+3)	27.25	32.25	428.52	668.232735	18.6
DIHHIDYY[+79.966331]K(+3)	27.25	32.25	428.52	390.142463	22.6
DIHHIDYY[+79.966331]K(+3)	27.25	32.25	428.52	528.220949	16.6
DIHHIDYY[+79.966331]K (heavy)(+3)	27.25	32.25	431.191	676.246934	18.6
DIHHIDYY[+79.966331]K (heavy)(+3)	27.25	32.25	431.191	398.156662	22.6
DIHHIDYY[+79.966331]K (heavy)(+3)	27.25	32.25	431.191	532.228049	16.6
DIHHIDY[+79.966331]YK(+3)	27.25	32.25	428.52	781.316799	18.6
DIHHIDY[+79.966331]YK(+3)	27.25	32.25	428.52	310.176132	22.6
DIHHIDY[+79.966331]YK(+3)	27.25	32.25	428.52	459.691493	17.6
DIHHIDY[+79.966331]YK (heavy)(+3)	27.25	32.25	431.191	789.330998	18.6
DIHHIDY[+79.966331]YK (heavy)(+3)	27.25	32.25	431.191	318.190331	22.6
DIHHIDY[+79.966331]YK (heavy)(+3)	27.25	32.25	431.191	463.698593	17.6
DIMNDSNY[+79.966331]IVK(+2)	56.66	61.66	696.299	803.369897	22.8
DIMNDSNY[+79.966331]IVK(+2)	56.66	61.66	696.299	602.294941	27.8
DIMNDSNY[+79.966331]IVK(+2)	56.66	61.66	696.299	229.118283	24.8
DIMNDSNY[+79.966331]IVK (heavy)(+2)	56.66	61.66	700.306	811.384096	22.8
DIMNDSNY[+79.966331]IVK (heavy)(+2)	56.66	61.66	700.306	610.30914	27.8
DIMNDSNY[+79.966331]IVK (heavy)(+2)	56.66	61.66	700.306	229.118283	24.8
DIM[+15.994915]NDSNY[+79.966331]IVK(+2)	44.68	49.68	704.296	803.369897	24
DIM[+15.994915]NDSNY[+79.966331]IVK(+2)	44.68	49.68	704.296	246.181218	24
DIM[+15.994915]NDSNY[+79.966331]IVK(+2)	44.68	49.68	704.296	229.118283	27
DIM[+15.994915]NDSNY[+79.966331]IVK (heavy)(+2)	44.68	49.68	708.303	811.384096	24
DIM[+15.994915]NDSNY[+79.966331]IVK (heavy)(+2)	44.68	49.68	708.303	254.195417	24
DIM[+15.994915]NDSNY[+79.966331]IVK (heavy)(+2)	44.68	49.68	708.303	229.118283	27
DIMSDSNY[+79.966331]VVR(+2)	55.59	60.59	689.789	1019.419367	21.6
DIMSDSNY[+79.966331]VVR(+2)	55.59	60.59	689.789	730.328367	23.6
DIMSDSNY[+79.966331]VVR(+2)	55.59	60.59	689.789	229.118283	24.6
DIMSDSNY[+79.966331]VVR (heavy)(+2)	55.59	60.59	694.793	1029.427636	21.6
DIMSDSNY[+79.966331]VVR (heavy)(+2)	55.59	60.59	694.793	740.336636	23.6

DIMSDSNY[+79.966331]VVR (heavy)(+2)	55.59	60.59	694.793	229.118283	24.6
DIM[+15.994915]SDSNY[+79.966331]VVR(+2)	41.87	46.87	697.786	817.360395	24.8
DIM[+15.994915]SDSNY[+79.966331]VVR(+2)	41.87	46.87	697.786	730.328367	23.8
DIM[+15.994915]SDSNY[+79.966331]VVR(+2)	41.87	46.87	697.786	229.118283	30.8
DIM[+15.994915]SDSNY[+79.966331]VVR (heavy)(+2)	41.87	46.87	702.79	827.368664	24.8
DIM[+15.994915]SDSNY[+79.966331]VVR (heavy)(+2)	41.87	46.87	702.79	740.336636	23.8
DIM[+15.994915]SDSNY[+79.966331]VVR (heavy)(+2)	41.87	46.87	702.79	229.118283	30.8
DINNIDY[+79.966331]YK(+2)	49.02	54.02	619.26	668.232735	19.5
DINNIDY[+79.966331]YK(+2)	49.02	54.02	619.26	310.176132	20.5
DINNIDY[+79.966331]YK(+2)	49.02	54.02	619.26	229.118283	19.5
DINNIDY[+79.966331]YK (heavy)(+2)	49.02	54.02	623.267	676.246934	19.5
DINNIDY[+79.966331]YK (heavy)(+2)	49.02	54.02	623.267	318.190331	20.5
DINNIDY[+79.966331]YK (heavy)(+2)	49.02	54.02	623.267	229.118283	19.5
DINNIDYY[+79.966331]K(+2)	45.03	50.03	619.26	668.232735	19.5
DINNIDYY[+79.966331]K(+2)	45.03	50.03	619.26	390.142463	29.5
DINNIDYY[+79.966331]K(+2)	45.03	50.03	619.26	229.118283	22.5
DINNIDYY[+79.966331]K (heavy)(+2)	45.03	50.03	623.267	676.246934	19.5
DINNIDYY[+79.966331]K (heavy)(+2)	45.03	50.03	623.267	398.156662	29.5
DINNIDYY[+79.966331]K (heavy)(+2)	45.03	50.03	623.267	229.118283	22.5
DINNIDY[+79.966331]Y[+79.966331]K(+2)	48.4	53.4	659.243	748.199066	20.7
DINNIDY[+79.966331]Y[+79.966331]K(+2)	48.4	53.4	659.243	633.172123	27.7
DINNIDY[+79.966331]Y[+79.966331]K(+2)	48.4	53.4	659.243	229.118283	24.7
DINNIDY[+79.966331]Y[+79.966331]K (heavy)(+2)	48.4	53.4	663.25	756.213265	20.7
DINNIDY[+79.966331]Y[+79.966331]K (heavy)(+2)	48.4	53.4	663.25	641.186322	27.7
DINNIDY[+79.966331]Y[+79.966331]K (heavy)(+2)	48.4	53.4	663.25	229.118283	24.7
DIYETDY[+79.966331]YR(+2)	48.92	53.92	659.255	1089.392483	20.7
DIYETDY[+79.966331]YR(+2)	48.92	53.92	659.255	338.18228	25.7
DIYETDY[+79.966331]YR(+2)	48.92	53.92	659.255	229.118283	23.7
DIYETDY[+79.966331]YR (heavy)(+2)	48.92	53.92	664.259	1099.400752	20.7
DIYETDY[+79.966331]YR (heavy)(+2)	48.92	53.92	664.259	348.190549	25.7
DIYETDY[+79.966331]YR (heavy)(+2)	48.92	53.92	664.259	229.118283	23.7
DIYETDYY[+79.966331]R(+2)	48.25	53.25	659.255	1089.392483	19.7
DIYETDYY[+79.966331]R(+2)	48.25	53.25	659.255	797.286561	21.7
DIYETDYY[+79.966331]R(+2)	48.25	53.25	659.255	418.148611	23.7
DIYETDYY[+79.966331]R (heavy)(+2)	48.25	53.25	664.259	1099.400752	19.7
DIYETDYY[+79.966331]R (heavy)(+2)	48.25	53.25	664.259	807.29483	21.7
DIYETDYY[+79.966331]R (heavy)(+2)	48.25	53.25	664.259	428.15688	23.7
DIY[+79.966331]ETDYYR(+2)	47.45	52.45	659.255	1089.392483	21.7
DIY[+79.966331]ETDYYR(+2)	47.45	52.45	659.255	501.245609	26.7
DIY[+79.966331]ETDYYR(+2)	47.45	52.45	659.255	229.118283	22.7
DIY[+79.966331]ETDYYR (heavy)(+2)	47.45	52.45	664.259	1099.400752	21.7

DIY[+79.966331]ETDYYR (heavy)(+2)	47.45	52.45	664.259	511.253878	26.7
DIY[+79.966331]ETDYYR (heavy)(+2)	47.45	52.45	664.259	229.118283	22.7
DIYSTDY[+79.966331]YR(+2)	48.51	53.51	638.25	884.31859	20.1
DIYSTDY[+79.966331]YR(+2)	48.51	53.51	638.25	338.18228	19.1
DIYSTDY[+79.966331]YR(+2)	48.51	53.51	638.25	229.118283	20.1
DIYSTDY[+79.966331]YR (heavy)(+2)	48.51	53.51	643.254	894.326859	20.1
DIYSTDY[+79.966331]YR (heavy)(+2)	48.51	53.51	643.254	348.190549	19.1
DIYSTDY[+79.966331]YR (heavy)(+2)	48.51	53.51	643.254	229.118283	20.1
DIYSTDYY[+79.966331]R(+2)	47.57	52.57	638.25	884.31859	19.1
DIYSTDYY[+79.966331]R(+2)	47.57	52.57	638.25	418.148611	22.1
DIYSTDYY[+79.966331]R(+2)	47.57	52.57	638.25	229.118283	20.1
DIYSTDYY[+79.966331]R (heavy)(+2)	47.57	52.57	643.254	894.326859	19.1
DIYSTDYY[+79.966331]R (heavy)(+2)	47.57	52.57	643.254	428.15688	22.1
DIYSTDYY[+79.966331]R (heavy)(+2)	47.57	52.57	643.254	229.118283	20.1
DIY[+79.966331]STDYYR(+2)	45.57	50.57	638.25	1047.381918	20.1
DIY[+79.966331]STDYYR(+2)	45.57	50.57	638.25	804.352259	23.1
DIY[+79.966331]STDYYR(+2)	45.57	50.57	638.25	229.118283	22.1
DIY[+79.966331]STDYYR (heavy)(+2)	45.57	50.57	643.254	1057.390187	20.1
DIY[+79.966331]STDYYR (heavy)(+2)	45.57	50.57	643.254	814.360528	23.1
DIY[+79.966331]STDYYR (heavy)(+2)	45.57	50.57	643.254	229.118283	22.1
NPDY[+79.966331]VR(+2)	16.3	21.3	422.173	632.243968	17.6
NPDY[+79.966331]VR(+2)	16.3	21.3	422.173	517.217025	18.6
NPDY[+79.966331]VR(+2)	16.3	21.3	422.173	365.152004	17.6
NPDY[+79.966331]VR (heavy)(+2)	16.3	21.3	427.177	642.252237	17.6
NPDY[+79.966331]VR (heavy)(+2)	16.3	21.3	427.177	527.225294	18.6
NPDY[+79.966331]VR (heavy)(+2)	16.3	21.3	427.177	370.156139	17.6
DPDY[+79.966331]VR(+2)	19.52	24.52	422.665	632.243968	17.6
DPDY[+79.966331]VR(+2)	19.52	24.52	422.665	517.217025	18.6
DPDY[+79.966331]VR(+2)	19.52	24.52	422.665	365.152004	17.6
DPDY[+79.966331]VR (heavy)(+2)	19.52	24.52	427.669	642.252237	17.6
DPDY[+79.966331]VR (heavy)(+2)	19.52	24.52	427.669	527.225294	18.6
DPDY[+79.966331]VR (heavy)(+2)	19.52	24.52	427.669	370.156139	17.6
DSNY[+79.966331]ISK(+2)	16.85	21.85	453.683	590.258556	17.5
DSNY[+79.966331]ISK(+2)	16.85	21.85	453.683	234.144832	20.5
DSNY[+79.966331]ISK(+2)	16.85	21.85	453.683	203.066248	16.5
DSNY[+79.966331]ISK (heavy)(+2)	16.85	21.85	457.69	598.272755	17.5
DSNY[+79.966331]ISK (heavy)(+2)	16.85	21.85	457.69	242.159031	20.5
DSNY[+79.966331]ISK (heavy)(+2)	16.85	21.85	457.69	203.066248	16.5
DVHNLDY[+79.966331]YK(+2)	36.76	41.76	623.76	553.205792	23.6
DVHNLDY[+79.966331]YK(+2)	36.76	41.76	623.76	310.176132	24.6
DVHNLDY[+79.966331]YK(+2)	36.76	41.76	623.76	352.161545	24.6

DVHNLDY[+79.966331]YK (heavy)(+2)	36.76	41.76	627.767	561.219991	23.6
DVHNLDY[+79.966331]YK (heavy)(+2)	36.76	41.76	627.767	318.190331	24.6
DVHNLDY[+79.966331]YK (heavy)(+2)	36.76	41.76	627.767	352.161545	24.6
DVHNLDY[+79.966331]Y[+79.966331]K(+2)	34.27	39.27	663.743	748.199066	26.8
DVHNLDY[+79.966331]Y[+79.966331]K(+2)	34.27	39.27	663.743	633.172123	27.8
DVHNLDY[+79.966331]Y[+79.966331]K(+2)	34.27	39.27	663.743	352.161545	26.8
DVHNLDY[+79.966331]Y[+79.966331]K (heavy)(+2)	34.27	39.27	667.75	756.213265	26.8
DVHNLDY[+79.966331]Y[+79.966331]K (heavy)(+2)	34.27	39.27	667.75	641.186322	27.8
DVHNLDY[+79.966331]Y[+79.966331]K (heavy)(+2)	34.27	39.27	667.75	352.161545	26.8
DVY[+79.966331]EEDSYVK(+2)	39.19	44.19	663.76	1112.418364	21.8
DVY[+79.966331]EEDSYVK(+2)	39.19	44.19	663.76	215.102633	21.8
DVY[+79.966331]EEDSYVK(+2)	39.19	44.19	663.76	458.132293	23.8
DVY[+79.966331]EEDSYVK (heavy)(+2)	39.19	44.19	667.767	1120.432563	21.8
DVY[+79.966331]EEDSYVK (heavy)(+2)	39.19	44.19	667.767	215.102633	21.8
DVY[+79.966331]EEDSYVK (heavy)(+2)	39.19	44.19	667.767	458.132293	23.8
DVYEEDSY[+79.966331]VK(+2)	38.26	43.26	663.76	1112.418364	19.8
DVYEEDSY[+79.966331]VK(+2)	38.26	43.26	663.76	489.210877	22.8
DVYEEDSY[+79.966331]VK(+2)	38.26	43.26	663.76	215.102633	23.8
DVYEEDSY[+79.966331]VK (heavy)(+2)	38.26	43.26	667.767	1120.432563	19.8
DVYEEDSY[+79.966331]VK (heavy)(+2)	38.26	43.26	667.767	497.225076	22.8
DVYEEDSY[+79.966331]VK (heavy)(+2)	38.26	43.26	667.767	215.102633	23.8
DVY[+79.966331]EEDSY[+79.966331]VK(+2)	38.28	43.28	703.743	489.210877	26
DVY[+79.966331]EEDSY[+79.966331]VK(+2)	38.28	43.28	703.743	215.102633	23
DVY[+79.966331]EEDSY[+79.966331]VK(+2)	38.28	43.28	703.743	458.132293	22
DVY[+79.966331]EEDSY[+79.966331]VK (heavy)(+2)	38.28	43.28	707.75	497.225076	26
DVY[+79.966331]EEDSY[+79.966331]VK (heavy)(+2)	38.28	43.28	707.75	215.102633	23
DVY[+79.966331]EEDSY[+79.966331]VK (heavy)(+2)	38.28	43.28	707.75	458.132293	22
DVYETDY[+79.966331]YR(+2)	43.59	48.59	652.247	1089.392483	19.5
DVYETDY[+79.966331]YR(+2)	43.59	48.59	652.247	797.286561	22.5
DVYETDY[+79.966331]YR(+2)	43.59	48.59	652.247	338.18228	20.5
DVYETDY[+79.966331]YR(+2)	43.59	48.59	652.247	215.102633	26.5
DVYETDY[+79.966331]YR (heavy)(+2)	43.59	48.59	657.251	1099.400752	19.5
DVYETDY[+79.966331]YR (heavy)(+2)	43.59	48.59	657.251	807.29483	22.5
DVYETDY[+79.966331]YR (heavy)(+2)	43.59	48.59	657.251	348.190549	20.5
DVYETDY[+79.966331]YR (heavy)(+2)	43.59	48.59	657.251	215.102633	26.5
DVYETDYY[+79.966331]R(+2)	43.59	48.59	652.247	1089.392483	19.5
DVYETDYY[+79.966331]R(+2)	43.59	48.59	652.247	797.286561	21.5
DVYETDYY[+79.966331]R(+2)	43.59	48.59	652.247	581.21194	25.5
DVYETDYY[+79.966331]R(+2)	43.59	48.59	652.247	209.577944	31.5
DVYETDYY[+79.966331]R (heavy)(+2)	43.59	48.59	657.251	1099.400752	19.5
DVYETDYY[+79.966331]R (heavy)(+2)	43.59	48.59	657.251	807.29483	21.5

DVYETDYY[+79.966331]R (heavy)(+2)	43.59	48.59	657.251	591.220209	25.5
DVYETDYY[+79.966331]R (heavy)(+2)	43.59	48.59	657.251	214.582078	31.5
DVYETDY[+79.966331]Y[+79.966331]R(+2)	44.01	49.01	692.23	877.252892	22.7
DVYETDY[+79.966331]Y[+79.966331]R(+2)	44.01	49.01	692.23	661.178271	26.7
DVYETDY[+79.966331]Y[+79.966331]R(+2)	44.01	49.01	692.23	585.183045	20.7
DVYETDY[+79.966331]Y[+79.966331]R (heavy)(+2)	44.01	49.01	697.234	887.261161	22.7
DVYETDY[+79.966331]Y[+79.966331]R (heavy)(+2)	44.01	49.01	697.234	671.18654	26.7
DVYETDY[+79.966331]Y[+79.966331]R (heavy)(+2)	44.01	49.01	697.234	590.18718	20.7
DVYSTDY[+79.966331]YR(+2)	42.91	47.91	631.242	884.31859	20.8
DVYSTDY[+79.966331]YR(+2)	42.91	47.91	631.242	797.286561	20.8
DVYSTDY[+79.966331]YR(+2)	42.91	47.91	631.242	215.102633	21.8
DVYSTDY[+79.966331]YR (heavy)(+2)	42.91	47.91	636.246	894.326859	20.8
DVYSTDY[+79.966331]YR (heavy)(+2)	42.91	47.91	636.246	807.29483	20.8
DVYSTDY[+79.966331]YR (heavy)(+2)	42.91	47.91	636.246	215.102633	21.8
DVYSTDYY[+79.966331]R(+2)	42.24	47.24	631.242	1047.381918	18.8
DVYSTDYY[+79.966331]R(+2)	42.24	47.24	631.242	884.31859	20.8
DVYSTDYY[+79.966331]R(+2)	42.24	47.24	631.242	797.286561	21.8
DVYSTDYY[+79.966331]R (heavy)(+2)	42.24	47.24	636.246	1057.390187	18.8
DVYSTDYY[+79.966331]R (heavy)(+2)	42.24	47.24	636.246	894.326859	20.8
DVYSTDYY[+79.966331]R (heavy)(+2)	42.24	47.24	636.246	807.29483	21.8
DVY[+79.966331]STDYYR(+2)	40.64	45.64	631.242	1047.381918	19.8
DVY[+79.966331]STDYYR(+2)	40.64	45.64	631.242	804.352259	21.8
DVY[+79.966331]STDYYR(+2)	40.64	45.64	631.242	215.102633	21.8
DVY[+79.966331]STDYYR (heavy)(+2)	40.64	45.64	636.246	1057.390187	19.8
DVY[+79.966331]STDYYR (heavy)(+2)	40.64	45.64	636.246	814.360528	21.8
DVY[+79.966331]STDYYR (heavy)(+2)	40.64	45.64	636.246	215.102633	21.8
DY[+79.966331]YVVR(+2)	38.06	43.06	447.691	536.319108	18.3
DY[+79.966331]YVVR(+2)	38.06	43.06	447.691	373.25578	19.3
DY[+79.966331]YVVR(+2)	38.06	43.06	447.691	359.063879	17.3
DY[+79.966331]YVVR (heavy)(+2)	38.06	43.06	452.695	546.327377	18.3
DY[+79.966331]YVVR (heavy)(+2)	38.06	43.06	452.695	383.264049	19.3
DY[+79.966331]YVVR (heavy)(+2)	38.06	43.06	452.695	359.063879	17.3
DYY[+79.966331]VVR(+2)	38.05	43.05	447.691	616.285439	17.3
DYY[+79.966331]VVR(+2)	38.05	43.05	447.691	373.25578	19.3
DYY[+79.966331]VVR(+2)	38.05	43.05	447.691	274.187366	19.3
DYY[+79.966331]VVR (heavy)(+2)	38.05	43.05	452.695	626.293708	17.3
DYY[+79.966331]VVR (heavy)(+2)	38.05	43.05	452.695	383.264049	19.3
DYY[+79.966331]VVR (heavy)(+2)	38.05	43.05	452.695	284.195635	19.3
DY[+79.966331]Y[+79.966331]VVR(+2)	39.13	44.13	487.674	616.285439	19.5
DY[+79.966331]Y[+79.966331]VVR(+2)	39.13	44.13	487.674	373.25578	21.5
DY[+79.966331]Y[+79.966331]VVR(+2)	39.13	44.13	487.674	274.187366	21.5

DY[+79.966331]Y[+79.966331]VVR (heavy)(+2)	39.13	44.13	492.678	626.293708	19.5
DY[+79.966331]Y[+79.966331]VVR (heavy)(+2)	39.13	44.13	492.678	383.264049	21.5
DY[+79.966331]Y[+79.966331]VVR (heavy)(+2)	39.13	44.13	492.678	284.195635	21.5
EDVY[+79.966331]LSHDHNIPYK(+3)	38.62	43.62	603.932	407.228896	26.2
EDVY[+79.966331]LSHDHNIPYK(+3)	38.62	43.62	603.932	733.826841	22.2
EDVY[+79.966331]LSHDHNIPYK(+3)	38.62	43.62	603.932	555.769979	22.2
EDVY[+79.966331]LSHDHNIPYK (heavy)(+3)	38.62	43.62	606.604	415.243095	26.2
EDVY[+79.966331]LSHDHNIPYK (heavy)(+3)	38.62	43.62	606.604	737.833941	22.2
EDVY[+79.966331]LSHDHNIPYK (heavy)(+3)	38.62	43.62	606.604	559.777079	22.2
EDVYLSHDHNIPY[+79.966331]K(+3)	37.29	42.29	603.932	487.195227	25.2
EDVYLSHDHNIPY[+79.966331]K(+3)	37.29	42.29	603.932	390.142463	28.2
EDVYLSHDHNIPY[+79.966331]K(+3)	37.29	42.29	603.932	245.076812	25.2
EDVYLSHDHNIPY[+79.966331]K (heavy)(+3)	37.29	42.29	606.604	495.209426	25.2
EDVYLSHDHNIPY[+79.966331]K (heavy)(+3)	37.29	42.29	606.604	398.156662	28.2
EDVYLSHDHNIPY[+79.966331]K (heavy)(+3)	37.29	42.29	606.604	245.076812	25.2
EDVY[+79.966331]LSHDHNIPY[+79.966331]K(+3)	39.17	44.17	630.588	487.195227	26.2
EDVY[+79.966331]LSHDHNIPY[+79.966331]K(+3)	39.17	44.17	630.588	390.142463	30.2
EDVY[+79.966331]LSHDHNIPY[+79.966331]K(+3)	39.17	44.17	630.588	595.753145	24.2
EDVY[+79.966331]LSHDHNIPY[+79.966331]K (heavy)(+3)	39.17	44.17	633.259	495.209426	26.2
EDVY[+79.966331]LSHDHNIPY[+79.966331]K (heavy)(+3)	39.17	44.17	633.259	398.156662	30.2
EDVY[+79.966331]LSHDHNIPY[+79.966331]K (heavy)(+3)	39.17	44.17	633.259	599.760244	24.2
EEADGVY[+79.966331]AASGGLR(+2)	41.88	46.88	737.814	874.381859	24
EEADGVY[+79.966331]AASGGLR(+2)	41.88	46.88	737.814	631.352199	27
EEADGVY[+79.966331]AASGGLR(+2)	41.88	46.88	737.814	489.277972	24
EEADGVY[+79.966331]AASGGLR (heavy)(+2)	41.88	46.88	742.818	884.390128	24
EEADGVY[+79.966331]AASGGLR (heavy)(+2)	41.88	46.88	742.818	641.360468	27
EEADGVY[+79.966331]AASGGLR (heavy)(+2)	41.88	46.88	742.818	499.286241	24
EIYSADY[+79.966331]YR(+2)	43.84	48.84	630.252	854.308025	20.8
EIYSADY[+79.966331]YR(+2)	43.84	48.84	630.252	767.275997	20.8
EIYSADY[+79.966331]YR(+2)	43.84	48.84	630.252	581.21194	25.8
EIYSADY[+79.966331]YR (heavy)(+2)	43.84	48.84	635.256	864.316294	20.8
EIYSADY[+79.966331]YR (heavy)(+2)	43.84	48.84	635.256	777.284266	20.8
EIYSADY[+79.966331]YR (heavy)(+2)	43.84	48.84	635.256	591.220209	25.8
ENIFGES[+79.966331]R(+2)	46.2	51.2	516.213	577.272886	21.4
ENIFGES[+79.966331]R(+2)	46.2	51.2	516.213	430.204472	22.4
ENIFGES[+79.966331]R(+2)	46.2	51.2	516.213	244.092797	20.4
ENIFGES[+79.966331]R (heavy)(+2)	46.2	51.2	521.217	587.281155	21.4
ENIFGES[+79.966331]R (heavy)(+2)	46.2	51.2	521.217	440.212741	22.4
ENIFGES[+79.966331]R (heavy)(+2)	46.2	51.2	521.217	244.092797	20.4
EPLAVVGS[+79.966331]PYWM[+15.994915]APEVLR(+2)	88.6	93.6	1055.507	684.403901	35.6
EPLAVVGS[+79.966331]PYWM[+15.994915]APEVLR(+2)	88.6	93.6	1055.507	613.366787	35.6

EPLAVVGS[+79.966331]PYWM[+15.994915]APEVLR(+2)	88.6	93.6	1055.507	288.203016	36.6
EPLAVVGS[+79.966331]PYWM[+15.994915]APEVLR (heavy)(+2)	88.6	93.6	1060.511	694.41217	35.6
EPLAVVGS[+79.966331]PYWM[+15.994915]APEVLR (heavy)(+2)	88.6	93.6	1060.511	623.375056	35.6
EPLAVVGS[+79.966331]PYWM[+15.994915]APEVLR (heavy)(+2)	88.6	93.6	1060.511	298.211285	36.6
EPLAVVGS[+79.966331]PYWMAPEVLR(+2)	96.74	101.74	1047.51	815.444385	34.3
EPLAVVGS[+79.966331]PYWMAPEVLR(+2)	96.74	101.74	1047.51	684.403901	34.3
EPLAVVGS[+79.966331]PYWMAPEVLR(+2)	96.74	101.74	1047.51	613.366787	35.3
EPLAVVGS[+79.966331]PYWMAPEVLR (heavy)(+2)	96.74	101.74	1052.514	825.452654	34.3
EPLAVVGS[+79.966331]PYWMAPEVLR (heavy)(+2)	96.74	101.74	1052.514	694.41217	34.3
EPLAVVGS[+79.966331]PYWMAPEVLR (heavy)(+2)	96.74	101.74	1052.514	623.375056	35.3
EVYAADYY[+79.966331]K(+2)	37.43	42.43	601.244	739.269849	18.9
EVYAADYY[+79.966331]K(+2)	37.43	42.43	601.244	390.142463	28.9
EVYAADYY[+79.966331]K(+2)	37.43	42.43	601.244	370.138562	20.9
EVYAADYY[+79.966331]K (heavy)(+2)	37.43	42.43	605.251	747.284048	18.9
EVYAADYY[+79.966331]K (heavy)(+2)	37.43	42.43	605.251	398.156662	28.9
EVYAADYY[+79.966331]K (heavy)(+2)	37.43	42.43	605.251	374.145662	20.9
EYGS[+79.966331]PLK(+2)	28.98	33.98	437.191	483.292559	19
EYGS[+79.966331]PLK(+2)	28.98	33.98	437.191	357.249632	13
EYGS[+79.966331]PLK(+2)	28.98	33.98	437.191	242.149917	19
EYGS[+79.966331]PLK (heavy)(+2)	28.98	33.98	441.198	491.306758	19
EYGS[+79.966331]PLK (heavy)(+2)	28.98	33.98	441.198	365.263831	13
EYGS[+79.966331]PLK (heavy)(+2)	28.98	33.98	441.198	246.157017	19
AY[+79.966331]T[+79.966331]PVVVTQWYR(+2)	85.99	90.99	821.856	951.504677	27.6
AY[+79.966331]T[+79.966331]PVVVTQWYR(+2)	85.99	90.99	821.856	852.436263	24.6
AY[+79.966331]T[+79.966331]PVVVTQWYR(+2)	85.99	90.99	821.856	398.111163	33.6
AY[+79.966331]T[+79.966331]PVVVTQWYR (heavy)(+2)	85.99	90.99	826.861	961.512946	27.6
AY[+79.966331]T[+79.966331]PVVVTQWYR (heavy)(+2)	85.99	90.99	826.861	862.444532	24.6
AY[+79.966331]T[+79.966331]PVVVTQWYR (heavy)(+2)	85.99	90.99	826.861	398.111163	33.6
AY[+79.966331]TPVVVTQWYR(+2)	71.05	76.05	781.873	951.504677	26.4
AY[+79.966331]TPVVVTQWYR(+2)	71.05	76.05	781.873	315.074049	30.4
AY[+79.966331]TPVVVTQWYR(+2)	71.05	76.05	781.873	416.121728	24.4
AY[+79.966331]TPVVVTQWYR (heavy)(+2)	71.05	76.05	786.877	961.512946	26.4
AY[+79.966331]TPVVVTQWYR (heavy)(+2)	71.05	76.05	786.877	315.074049	30.4
AY[+79.966331]TPVVVTQWYR (heavy)(+2)	71.05	76.05	786.877	416.121728	24.4
EY[+79.966331]YSVHNK(+2)	16.32	21.32	560.228	747.378414	20.7
EY[+79.966331]YSVHNK(+2)	16.32	21.32	560.228	398.214643	23.7
EY[+79.966331]YSVHNK(+2)	16.32	21.32	560.228	261.155731	24.7
EY[+79.966331]YSVHNK(+2)	16.32	21.32	560.228	373.079529	21.7
EY[+79.966331]YSVHNK (heavy)(+2)	16.32	21.32	564.236	755.392613	20.7
EY[+79.966331]YSVHNK (heavy)(+2)	16.32	21.32	564.236	406.228842	23.7
EY[+79.966331]YSVHNK (heavy)(+2)	16.32	21.32	564.236	269.16993	24.7

EY[+79.966331]YSVHNK (heavy)(+2)	16.32	21.32	564.236	373.079529	21.7
EYY[+79.966331]SVHNK(+2)	14.18	19.18	560.228	827.344745	20.7
EYY[+79.966331]SVHNK(+2)	14.18	19.18	560.228	398.214643	22.7
EYY[+79.966331]SVHNK(+2)	14.18	19.18	560.228	261.155731	23.7
EYY[+79.966331]SVHNK(+2)	14.18	19.18	560.228	293.113198	19.7
EYY[+79.966331]SVHNK (heavy)(+2)	14.18	19.18	564.236	835.358944	20.7
EYY[+79.966331]SVHNK (heavy)(+2)	14.18	19.18	564.236	406.228842	22.7
EYY[+79.966331]SVHNK (heavy)(+2)	14.18	19.18	564.236	269.16993	23.7
EYY[+79.966331]SVHNK (heavy)(+2)	14.18	19.18	564.236	293.113198	19.7
EY[+79.966331]Y[+79.966331]SVHNK(+2)	16.05	21.05	600.212	497.283057	22.9
EY[+79.966331]Y[+79.966331]SVHNK(+2)	16.05	21.05	600.212	398.214643	24.9
EY[+79.966331]Y[+79.966331]SVHNK(+2)	16.05	21.05	600.212	261.155731	25.9
EY[+79.966331]Y[+79.966331]SVHNK (heavy)(+2)	16.05	21.05	604.219	505.297256	22.9
EY[+79.966331]Y[+79.966331]SVHNK (heavy)(+2)	16.05	21.05	604.219	406.228842	24.9
EY[+79.966331]Y[+79.966331]SVHNK (heavy)(+2)	16.05	21.05	604.219	269.16993	25.9
EY[+79.966331]YSVQQHR(+2)	21.46	26.46	645.269	754.395461	25.3
EY[+79.966331]YSVQQHR(+2)	21.46	26.46	645.269	568.295019	26.3
EY[+79.966331]YSVQQHR(+2)	21.46	26.46	645.269	373.079529	26.3
EY[+79.966331]YSVQQHR (heavy)(+2)	21.46	26.46	650.273	764.40373	25.3
EY[+79.966331]YSVQQHR (heavy)(+2)	21.46	26.46	650.273	578.303288	26.3
EY[+79.966331]YSVQQHR (heavy)(+2)	21.46	26.46	650.273	373.079529	26.3
EYY[+79.966331]SVQQHR(+2)	19.32	24.32	645.269	568.295019	25.3
EYY[+79.966331]SVQQHR(+2)	19.32	24.32	645.269	440.236441	27.3
EYY[+79.966331]SVQQHR(+2)	19.32	24.32	645.269	312.177864	26.3
EYY[+79.966331]SVQQHR(+2)	19.32	24.32	645.269	293.113198	27.3
EYY[+79.966331]SVQQHR (heavy)(+2)	19.32	24.32	650.273	578.303288	25.3
EYY[+79.966331]SVQQHR (heavy)(+2)	19.32	24.32	650.273	450.24471	27.3
EYY[+79.966331]SVQQHR (heavy)(+2)	19.32	24.32	650.273	322.186133	26.3
EYY[+79.966331]SVQQHR (heavy)(+2)	19.32	24.32	650.273	293.113198	27.3
EY[+79.966331]Y[+79.966331]SVQQHR(+2)	19.48	24.48	685.252	754.395461	28.5
EY[+79.966331]Y[+79.966331]SVQQHR(+2)	19.48	24.48	685.252	568.295019	28.5
EY[+79.966331]Y[+79.966331]SVQQHR(+2)	19.48	24.48	685.252	440.236441	28.5
EY[+79.966331]Y[+79.966331]SVQQHR (heavy)(+2)	19.48	24.48	690.256	764.40373	28.5
EY[+79.966331]Y[+79.966331]SVQQHR (heavy)(+2)	19.48	24.48	690.256	578.303288	28.5
EY[+79.966331]Y[+79.966331]SVQQHR (heavy)(+2)	19.48	24.48	690.256	450.24471	28.5
EY[+79.966331]YTVK(+2)	26.46	31.46	441.685	510.292225	17.2
EY[+79.966331]YTVK(+2)	26.46	31.46	441.685	347.228896	19.2
EY[+79.966331]YTVK(+2)	26.46	31.46	441.685	373.079529	16.2
EY[+79.966331]YTVK (heavy)(+2)	26.46	31.46	445.692	518.306424	17.2
EY[+79.966331]YTVK (heavy)(+2)	26.46	31.46	445.692	355.243095	19.2
EY[+79.966331]YTVK (heavy)(+2)	26.46	31.46	445.692	373.079529	16.2

EYY[+79.966331]TVK(+2)	23.52	28.52	441.685	590.258556	17.2
EYY[+79.966331]TVK(+2)	23.52	28.52	441.685	347.228896	17.2
EYY[+79.966331]TVK(+2)	23.52	28.52	441.685	246.181218	17.2
EYY[+79.966331]TVK (heavy)(+2)	23.52	28.52	445.692	598.272755	17.2
EYY[+79.966331]TVK (heavy)(+2)	23.52	28.52	445.692	355.243095	17.2
EYY[+79.966331]TVK (heavy)(+2)	23.52	28.52	445.692	254.195417	17.2
EY[+79.966331]Y[+79.966331]TVK(+2)	24.21	29.21	481.669	590.258556	20.4
EY[+79.966331]Y[+79.966331]TVK(+2)	24.21	29.21	481.669	347.228896	23.4
EY[+79.966331]Y[+79.966331]TVK(+2)	24.21	29.21	481.669	246.181218	20.4
EY[+79.966331]Y[+79.966331]TVK (heavy)(+2)	24.21	29.21	485.676	598.272755	20.4
EY[+79.966331]Y[+79.966331]TVK (heavy)(+2)	24.21	29.21	485.676	355.243095	23.4
EY[+79.966331]Y[+79.966331]TVK (heavy)(+2)	24.21	29.21	485.676	254.195417	20.4
FAQTVM[+15.994915]TS[+79.966331]R(+2)	26.88	31.88	568.743	790.316482	17
FAQTVM[+15.994915]TS[+79.966331]R(+2)	26.88	31.88	568.743	342.117311	20
FAQTVM[+15.994915]TS[+79.966331]R(+2)	26.88	31.88	568.743	219.112804	22
FAQTVM[+15.994915]TS[+79.966331]R (heavy)(+2)	26.88	31.88	573.748	800.324751	17
FAQTVM[+15.994915]TS[+79.966331]R (heavy)(+2)	26.88	31.88	573.748	352.12558	20
FAQTVM[+15.994915]TS[+79.966331]R (heavy)(+2)	26.88	31.88	573.748	219.112804	22
FAQTVMTS[+79.966331]R(+2)	36.48	41.48	560.746	774.321567	17.7
FAQTVMTS[+79.966331]R(+2)	36.48	41.48	560.746	676.344671	23.7
FAQTVMTS[+79.966331]R(+2)	36.48	41.48	560.746	575.296992	23.7
FAQTVMTS[+79.966331]R (heavy)(+2)	36.48	41.48	565.75	784.329836	17.7
FAQTVMTS[+79.966331]R (heavy)(+2)	36.48	41.48	565.75	686.35294	23.7
FAQTVMTS[+79.966331]R (heavy)(+2)	36.48	41.48	565.75	585.305261	23.7
FAQTVM[+15.994915]T[+79.966331]SR(+2)	24.36	29.36	568.743	790.316482	18
FAQTVM[+15.994915]T[+79.966331]SR(+2)	24.36	29.36	568.743	262.15098	24
FAQTVM[+15.994915]T[+79.966331]SR(+2)	24.36	29.36	568.743	219.112804	25
FAQTVM[+15.994915]T[+79.966331]SR (heavy)(+2)	24.36	29.36	573.748	800.324751	18
FAQTVM[+15.994915]T[+79.966331]SR (heavy)(+2)	24.36	29.36	573.748	272.159249	24
FAQTVM[+15.994915]T[+79.966331]SR (heavy)(+2)	24.36	29.36	573.748	219.112804	25
FAQTVMT[+79.966331]SR(+2)	35.55	40.55	560.746	774.321567	19.7
FAQTVMT[+79.966331]SR(+2)	35.55	40.55	560.746	676.344671	23.7
FAQTVMT[+79.966331]SR(+2)	35.55	40.55	560.746	476.228578	22.7
FAQTVMT[+79.966331]SR (heavy)(+2)	35.55	40.55	565.75	784.329836	19.7
FAQTVMT[+79.966331]SR (heavy)(+2)	35.55	40.55	565.75	686.35294	23.7
FAQTVMT[+79.966331]SR (heavy)(+2)	35.55	40.55	565.75	486.236847	22.7
FFSSETTAAHS[+79.966331]LVGTPYYMSPER(+3)	68.41	73.41	886.724	1200.535385	32
FFSSETTAAHS[+79.966331]LVGTPYYMSPER(+3)	68.41	73.41	886.724	782.35015	34
FFSSETTAAHS[+79.966331]LVGTPYYMSPER(+3)	68.41	73.41	886.724	619.286822	34
FFSSETTAAHS[+79.966331]LVGTPYYMSPER (heavy)(+3)	68.41	73.41	890.06	1210.543654	32
FFSSETTAAHS[+79.966331]LVGTPYYMSPER (heavy)(+3)	68.41	73.41	890.06	792.358419	34

FFSSETTAAHS[+79.966331]LVGTPYYMSPER (heavy)(+3)	68.41	73.41	890.06	629.295091	34
FFSSETTAAHS[+79.966331]LVGTPYYM[+15.994915]SPER(+3)	65.1	70.1	892.055	1216.5303	32.2
FFSSETTAAHS[+79.966331]LVGTPYYM[+15.994915]SPER(+3)	65.1	70.1	892.055	1058.461158	32.2
FFSSETTAAHS[+79.966331]LVGTPYYM[+15.994915]SPER(+3)	65.1	70.1	892.055	994.462873	35.2
FFSSETTAAHS[+79.966331]LVGTPYYM[+15.994915]SPER (heavy)(+3)	65.1	70.1	895.391	1226.538569	32.2
FFSSETTAAHS[+79.966331]LVGTPYYM[+15.994915]SPER (heavy)(+3)	65.1	70.1	895.391	1068.469427	32.2
FFSSETTAAHS[+79.966331]LVGTPYYM[+15.994915]SPER (heavy)(+3)	65.1	70.1	895.391	1004.471142	35.2
FFSSETT[+79.966331]AAHSLVGTPYYMSPER(+3)	68.41	73.41	886.724	1042.466243	32
FFSSETT[+79.966331]AAHSLVGTPYYMSPER(+3)	68.41	73.41	886.724	488.246337	33
FFSSETT[+79.966331]AAHSLVGTPYYMSPER(+3)	68.41	73.41	886.724	295.144104	33
FFSSETT[+79.966331]AAHSLVGTPYYMSPER (heavy)(+3)	68.41	73.41	890.06	1052.474512	32
FFSSETT[+79.966331]AAHSLVGTPYYMSPER (heavy)(+3)	68.41	73.41	890.06	498.254606	33
FFSSETT[+79.966331]AAHSLVGTPYYMSPER (heavy)(+3)	68.41	73.41	890.06	295.144104	33
FFSSETT[+79.966331]AAHSLVGTPYYM[+15.994915]SPER(+3)	65.1	70.1	892.055	1058.461158	36.2
FFSSETT[+79.966331]AAHSLVGTPYYM[+15.994915]SPER(+3)	65.1	70.1	892.055	401.214309	36.2
FFSSETT[+79.966331]AAHSLVGTPYYM[+15.994915]SPER(+3)	65.1	70.1	892.055	529.734217	36.2
FFSSETT[+79.966331]AAHSLVGTPYYM[+15.994915]SPER (heavy)(+3)	65.1	70.1	895.391	1068.469427	36.2
FFSSETT[+79.966331]AAHSLVGTPYYM[+15.994915]SPER (heavy)(+3)	65.1	70.1	895.391	411.222578	36.2
FFSSETT[+79.966331]AAHSLVGTPYYM[+15.994915]SPER (heavy)(+3)	65.1	70.1	895.391	534.738351	36.2
FVLDDQY[+79.966331]TSSTGTK(+2)	47.49	52.49	821.355	681.34136	28.5
FVLDDQY[+79.966331]TSSTGTK(+2)	47.49	52.49	821.355	305.181946	28.5
FVLDDQY[+79.966331]TSSTGTK(+2)	47.49	52.49	821.355	247.144104	30.5
FVLDDQY[+79.966331]TSSTGTK (heavy)(+2)	47.49	52.49	825.362	689.355559	28.5
FVLDDQY[+79.966331]TSSTGTK (heavy)(+2)	47.49	52.49	825.362	313.196145	28.5
FVLDDQY[+79.966331]TSSTGTK (heavy)(+2)	47.49	52.49	825.362	247.144104	30.5
FVS[+79.966331]VYGTEEYLHPDM[+15.994915]YER(+3)	68.51	73.51	777.66	899.400607	32.8
FVS[+79.966331]VYGTEEYLHPDM[+15.994915]YER(+3)	68.51	73.51	777.66	762.341695	34.8
FVS[+79.966331]VYGTEEYLHPDM[+15.994915]YER(+3)	68.51	73.51	777.66	316.165567	31.8
FVS[+79.966331]VYGTEEYLHPDM[+15.994915]YER (heavy)(+3)	68.51	73.51	780.996	909.408876	32.8
FVS[+79.966331]VYGTEEYLHPDM[+15.994915]YER (heavy)(+3)	68.51	73.51	780.996	772.349964	34.8
FVS[+79.966331]VYGTEEYLHPDM[+15.994915]YER (heavy)(+3)	68.51	73.51	780.996	316.165567	31.8
FVS[+79.966331]VYGTEEYLHPDMYER(+3)	73.31	78.31	772.329	810.345065	30.6
FVS[+79.966331]VYGTEEYLHPDMYER(+3)	73.31	78.31	772.329	598.265358	34.6
FVS[+79.966331]VYGTEEYLHPDMYER(+3)	73.31	78.31	772.329	316.165567	31.6
FVS[+79.966331]VYGTEEYLHPDMYER (heavy)(+3)	73.31	78.31	775.665	820.353334	30.6
FVS[+79.966331]VYGTEEYLHPDMYER (heavy)(+3)	73.31	78.31	775.665	608.273627	34.6
FVS[+79.966331]VYGTEEYLHPDMYER (heavy)(+3)	73.31	78.31	775.665	316.165567	31.6
GAILT[+79.966331]T[+79.966331]M[+15.994915]LATR(+2)	62.51	67.51	662.292	460.287808	22.8
GAILT[+79.966331]T[+79.966331]M[+15.994915]LATR(+2)	62.51	67.51	662.292	347.203744	20.8
GAILT[+79.966331]T[+79.966331]M[+15.994915]LATR(+2)	62.51	67.51	662.292	242.149918	25.8
GAILT[+79.966331]T[+79.966331]M[+15.994915]LATR (heavy)(+2)	62.51	67.51	667.296	470.296077	22.8

GAILT[+79.966331]T[+79.966331]M[+15.994915]LATR (heavy)(+2)	62.51	67.51	667.296	357.212013	20.8
GAILT[+79.966331]T[+79.966331]M[+15.994915]LATR (heavy)(+2)	62.51	67.51	667.296	242.149918	25.8
GAILT[+79.966331]T[+79.966331]MLATR(+2)	83.16	88.16	654.295	591.328293	23.5
GAILT[+79.966331]T[+79.966331]MLATR(+2)	83.16	88.16	654.295	347.203744	19.5
GAILT[+79.966331]T[+79.966331]MLATR(+2)	83.16	88.16	654.295	242.149918	25.5
GAILT[+79.966331]T[+79.966331]MLATR (heavy)(+2)	83.16	88.16	659.299	601.336562	23.5
GAILT[+79.966331]T[+79.966331]MLATR (heavy)(+2)	83.16	88.16	659.299	357.212013	19.5
GAILT[+79.966331]T[+79.966331]MLATR (heavy)(+2)	83.16	88.16	659.299	242.149918	25.5
GAILT[+79.966331]TM[+15.994915]LATR(+2)	53.43	58.43	622.309	791.408	23.6
GAILT[+79.966331]TM[+15.994915]LATR(+2)	53.43	58.43	622.309	708.370886	21.6
GAILT[+79.966331]TM[+15.994915]LATR(+2)	53.43	58.43	622.309	276.16663	19.6
GAILT[+79.966331]TM[+15.994915]LATR(+2)	53.43	58.43	622.309	242.149918	23.6
GAILT[+79.966331]TM[+15.994915]LATR (heavy)(+2)	53.43	58.43	627.313	801.416269	23.6
GAILT[+79.966331]TM[+15.994915]LATR (heavy)(+2)	53.43	58.43	627.313	718.379155	21.6
GAILT[+79.966331]TM[+15.994915]LATR (heavy)(+2)	53.43	58.43	627.313	286.174899	19.6
GAILT[+79.966331]TM[+15.994915]LATR (heavy)(+2)	53.43	58.43	627.313	242.149918	23.6
GAILT[+79.966331]TMLATR(+2)	75.95	80.95	614.311	460.287808	17.3
GAILT[+79.966331]TMLATR(+2)	75.95	80.95	614.311	347.203744	17.3
GAILT[+79.966331]TMLATR(+2)	75.95	80.95	614.311	242.149918	24.3
GAILT[+79.966331]TMLATR (heavy)(+2)	75.95	80.95	619.316	470.296077	17.3
GAILT[+79.966331]TMLATR (heavy)(+2)	75.95	80.95	619.316	357.212013	17.3
GAILT[+79.966331]TMLATR (heavy)(+2)	75.95	80.95	619.316	242.149918	24.3
GAILTT[+79.966331]M[+15.994915]LATR(+2)	51.03	56.03	622.309	690.360321	24.6
GAILTT[+79.966331]M[+15.994915]LATR(+2)	51.03	56.03	622.309	460.287808	21.6
GAILTT[+79.966331]M[+15.994915]LATR(+2)	51.03	56.03	622.309	347.203744	19.6
GAILTT[+79.966331]M[+15.994915]LATR (heavy)(+2)	51.03	56.03	627.313	700.36859	24.6
GAILTT[+79.966331]M[+15.994915]LATR (heavy)(+2)	51.03	56.03	627.313	470.296077	21.6
GAILTT[+79.966331]M[+15.994915]LATR (heavy)(+2)	51.03	56.03	627.313	357.212013	19.6
GAILTT[+79.966331]MLATR(+2)	66.61	71.61	614.311	347.203744	18.3
GAILTT[+79.966331]MLATR(+2)	66.61	71.61	614.311	276.16663	17.3
GAILTT[+79.966331]MLATR(+2)	66.61	71.61	614.311	242.149918	23.3
GAILTT[+79.966331]MLATR (heavy)(+2)	66.61	71.61	619.316	357.212013	18.3
GAILTT[+79.966331]MLATR (heavy)(+2)	66.61	71.61	619.316	286.174899	17.3
GAILTT[+79.966331]MLATR (heavy)(+2)	66.61	71.61	619.316	242.149918	23.3
GDVM[+15.994915]ST[+79.966331]AC[+57.021464]GTPGYVAPEVLAQK(+3)	61.49	66.49	783.016	954.561858	24
GDVM[+15.994915]ST[+79.966331]AC[+57.021464]GTPGYVAPEVLAQK(+3)	61.49	66.49	783.016	855.493444	24
GDVM[+15.994915]ST[+79.966331]AC[+57.021464]GTPGYVAPEVLAQK(+3)	61.49	66.49	783.016	784.45633	28
GDVM[+15.994915]ST[+79.966331]AC[+57.021464]GTPGYVAPEVLAQK (heavy)(+3)	61.49	66.49	785.687	962.576057	24
GDVM[+15.994915]ST[+79.966331]AC[+57.021464]GTPGYVAPEVLAQK (heavy)(+3)	61.49	66.49	785.687	863.507643	24
GDVM[+15.994915]ST[+79.966331]AC[+57.021464]GTPGYVAPEVLAQK (heavy)(+3)	61.49	66.49	785.687	792.470529	28
GDVMST[+79.966331]AC[+57.021464]GTPGYVAPEVLAQK(+3)	67.99	72.99	777.684	855.493444	27.8

GDVMST[+79.966331]AC[+57.021464]GTPGYVAPEVLAQK(+3)	67.99	72.99	777.684	784.45633	28.8
GDVMST[+79.966331]AC[+57.021464]GTPGYVAPEVLAQK(+3)	67.99	72.99	777.684	392.731803	28.8
GDVMST[+79.966331]AC[+57.021464]GTPGYVAPEVLAQK (heavy)(+3)	67.99	72.99	780.356	863.507643	27.8
GDVMST[+79.966331]AC[+57.021464]GTPGYVAPEVLAQK (heavy)(+3)	67.99	72.99	780.356	792.470529	28.8
GDVMST[+79.966331]AC[+57.021464]GTPGYVAPEVLAQK (heavy)(+3)	67.99	72.99	780.356	396.738903	28.8
GEPNVSY[+79.966331]IC[+57.021464]SR(+2)	41.47	46.47	681.281	964.395795	27.3
GEPNVSY[+79.966331]IC[+57.021464]SR(+2)	41.47	46.47	681.281	865.327381	24.3
GEPNVSY[+79.966331]IC[+57.021464]SR(+2)	41.47	46.47	681.281	535.265693	28.3
GEPNVSY[+79.966331]IC[+57.021464]SR (heavy)(+2)	41.47	46.47	686.285	974.404064	27.3
GEPNVSY[+79.966331]IC[+57.021464]SR (heavy)(+2)	41.47	46.47	686.285	875.33565	24.3
GEPNVSY[+79.966331]IC[+57.021464]SR (heavy)(+2)	41.47	46.47	686.285	545.273962	28.3
GHLS[+79.966331]EGLVTK(+2)	31.01	36.01	560.773	828.482545	22.7
GHLS[+79.966331]EGLVTK(+2)	31.01	36.01	560.773	248.160482	22.7
GHLS[+79.966331]EGLVTK(+2)	31.01	36.01	560.773	195.087652	24.7
GHLS[+79.966331]EGLVTK (heavy)(+2)	31.01	36.01	564.78	836.496744	22.7
GHLS[+79.966331]EGLVTK (heavy)(+2)	31.01	36.01	564.78	256.174681	22.7
GHLS[+79.966331]EGLVTK (heavy)(+2)	31.01	36.01	564.78	195.087652	24.7
GQEVY[+79.966331]VK(+2)	22.2	27.2	451.704	489.210877	21.5
GQEVY[+79.966331]VK(+2)	22.2	27.2	451.704	186.087317	15.5
GQEVY[+79.966331]VK(+2)	22.2	27.2	451.704	315.12991	15.5
GQEVY[+79.966331]VK (heavy)(+2)	22.2	27.2	455.711	497.225076	21.5
GQEVY[+79.966331]VK (heavy)(+2)	22.2	27.2	455.711	186.087317	15.5
GQEVY[+79.966331]VK (heavy)(+2)	22.2	27.2	455.711	315.12991	15.5
GS[+79.966331]AAWM[+15.994915]APEVFEGSNYSEK(+2)	70.33	75.33	1078.437	1060.458181	34.3
GS[+79.966331]AAWM[+15.994915]APEVFEGSNYSEK(+2)	70.33	75.33	1078.437	602.239143	39.3
GS[+79.966331]AAWM[+15.994915]APEVFEGSNYSEK(+2)	70.33	75.33	1078.437	673.276257	38.3
GS[+79.966331]AAWM[+15.994915]APEVFEGSNYSEK (heavy)(+2)	70.33	75.33	1082.444	1068.47238	34.3
GS[+79.966331]AAWM[+15.994915]APEVFEGSNYSEK (heavy)(+2)	70.33	75.33	1082.444	602.239143	39.3
GS[+79.966331]AAWM[+15.994915]APEVFEGSNYSEK (heavy)(+2)	70.33	75.33	1082.444	673.276257	38.3
GS[+79.966331]AAWMAPEVFEGSNYSEK(+2)	77.8	82.8	1070.44	455.203744	37
GS[+79.966331]AAWMAPEVFEGSNYSEK(+2)	77.8	82.8	1070.44	586.244228	34
GS[+79.966331]AAWMAPEVFEGSNYSEK(+2)	77.8	82.8	1070.44	657.281342	35
GS[+79.966331]AAWMAPEVFEGSNYSEK (heavy)(+2)	77.8	82.8	1074.447	455.203744	37
GS[+79.966331]AAWMAPEVFEGSNYSEK (heavy)(+2)	77.8	82.8	1074.447	586.244228	34
GS[+79.966331]AAWMAPEVFEGSNYSEK (heavy)(+2)	77.8	82.8	1074.447	657.281342	35
GS[+79.966331]FDGSSSQPSR(+2)	21.87	26.87	646.251	805.379871	26.3
GS[+79.966331]FDGSSSQPSR(+2)	21.87	26.87	646.251	661.326378	26.3
GS[+79.966331]FDGSSSQPSR(+2)	21.87	26.87	646.251	359.203744	23.3
GS[+79.966331]FDGSSSQPSR (heavy)(+2)	21.87	26.87	651.255	815.38814	26.3
GS[+79.966331]FDGSSSQPSR (heavy)(+2)	21.87	26.87	651.255	671.334647	26.3
GS[+79.966331]FDGSSSQPSR (heavy)(+2)	21.87	26.87	651.255	369.212013	23.3

GS[+79.966331]PLYMAPEMVC[+57.021464]QR(+2)	65.35	70.35	859.858	1121.490032	29.7
GS[+79.966331]PLYMAPEMVC[+57.021464]QR(+2)	65.35	70.35	859.858	919.412433	30.7
GS[+79.966331]PLYMAPEMVC[+57.021464]QR(+2)	65.35	70.35	859.858	337.187031	30.7
GS[+79.966331]PLYMAPEMVC[+57.021464]QR (heavy)(+2)	65.35	70.35	864.862	1131.498301	29.7
GS[+79.966331]PLYMAPEMVC[+57.021464]QR (heavy)(+2)	65.35	70.35	864.862	929.420702	30.7
GS[+79.966331]PLYMAPEMVC[+57.021464]QR (heavy)(+2)	65.35	70.35	864.862	337.187031	30.7
GS[+79.966331]PLYM[+15.994915]APEM[+15.994915]VC[+57.021464]QR(+2)	45.91	50.91	875.853	942.446177	33.2
GS[+79.966331]PLYM[+15.994915]APEM[+15.994915]VC[+57.021464]QR(+2)	45.91	50.91	875.853	935.407348	27.2
GS[+79.966331]PLYM[+15.994915]APEM[+15.994915]VC[+57.021464]QR(+2)	45.91	50.91	875.853	718.322873	29.2
GS[+79.966331]PLYM[+15.994915]APEM[+15.994915]VC[+57.021464]QR (heavy)(+2)	45.91	50.91	880.857	952.454446	33.2
GS[+79.966331]PLYM[+15.994915]APEM[+15.994915]VC[+57.021464]QR (heavy)(+2)	45.91	50.91	880.857	945.415617	27.2
GS[+79.966331]PLYM[+15.994915]APEM[+15.994915]VC[+57.021464]QR (heavy)(+2)	45.91	50.91	880.857	718.322873	29.2
GS[+79.966331]PLYM[+15.994915]APEMVC[+57.021464]QR(+2)	56.3	61.3	867.856	919.412433	28.9
GS[+79.966331]PLYM[+15.994915]APEMVC[+57.021464]QR(+2)	56.3	61.3	867.856	718.322873	28.9
GS[+79.966331]PLYM[+15.994915]APEMVC[+57.021464]QR (heavy)(+2)	56.3	61.3	872.86	929.420702	28.9
GS[+79.966331]PLYM[+15.994915]APEMVC[+57.021464]QR (heavy)(+2)	56.3	61.3	872.86	718.322873	28.9
GS[+79.966331]PLYMAPEM[+15.994915]VC[+57.021464]QR(+2)	54.71	59.71	867.856	942.446177	28.9
GS[+79.966331]PLYMAPEM[+15.994915]VC[+57.021464]QR(+2)	54.71	59.71	867.856	935.407348	28.9
GS[+79.966331]PLYMAPEM[+15.994915]VC[+57.021464]QR(+2)	54.71	59.71	867.856	702.327958	28.9
GS[+79.966331]PLYMAPEM[+15.994915]VC[+57.021464]QR (heavy)(+2)	54.71	59.71	872.86	952.454446	28.9
GS[+79.966331]PLYMAPEM[+15.994915]VC[+57.021464]QR (heavy)(+2)	54.71	59.71	872.86	945.415617	28.9
GS[+79.966331]PLYMAPEM[+15.994915]VC[+57.021464]QR (heavy)(+2)	54.71	59.71	872.86	702.327958	28.9
GT[+79.966331]EIYM[+15.994915]SPEVILC[+57.021464]R(+2)	68.54	73.54	882.39	973.513528	29.4
GT[+79.966331]EIYM[+15.994915]SPEVILC[+57.021464]R(+2)	68.54	73.54	882.39	886.481499	34.4
GT[+79.966331]EIYM[+15.994915]SPEVILC[+57.021464]R(+2)	68.54	73.54	882.39	270.108446	38.4
GT[+79.966331]EIYM[+15.994915]SPEVILC[+57.021464]R (heavy)(+2)	68.54	73.54	887.394	983.521797	29.4
GT[+79.966331]EIYM[+15.994915]SPEVILC[+57.021464]R (heavy)(+2)	68.54	73.54	887.394	896.489768	34.4
GT[+79.966331]EIYM[+15.994915]SPEVILC[+57.021464]R (heavy)(+2)	68.54	73.54	887.394	270.108446	38.4
GT[+79.966331]EIYMSPEVILC[+57.021464]R(+2)	80.54	85.54	874.393	973.513528	25.1
GT[+79.966331]EIYMSPEVILC[+57.021464]R(+2)	80.54	85.54	874.393	886.481499	28.1
GT[+79.966331]EIYMSPEVILC[+57.021464]R(+2)	80.54	85.54	874.393	270.108446	31.1
GT[+79.966331]EIYMSPEVILC[+57.021464]R (heavy)(+2)	80.54	85.54	879.397	983.521797	25.1
GT[+79.966331]EIYMSPEVILC[+57.021464]R (heavy)(+2)	80.54	85.54	879.397	896.489768	28.1
GT[+79.966331]EIYMSPEVILC[+57.021464]R (heavy)(+2)	80.54	85.54	879.397	270.108446	31.1
GT[+79.966331]LAYLPEEYIK(+2)	74.77	79.77	738.854	778.398146	23.1
GT[+79.966331]LAYLPEEYIK(+2)	74.77	79.77	738.854	254.149917	27.1
GT[+79.966331]LAYLPEEYIK(+2)	74.77	79.77	738.854	488.25036	28.1
GT[+79.966331]LAYLPEEYIK (heavy)(+2)	74.77	79.77	742.861	786.412345	23.1
GT[+79.966331]LAYLPEEYIK (heavy)(+2)	74.77	79.77	742.861	254.149917	27.1
GT[+79.966331]LAYLPEEYIK (heavy)(+2)	74.77	79.77	742.861	488.25036	28.1
GVHHIDY[+79.966331]YK(+3)	21.51	26.51	404.513	668.232735	17.7

GVHHIDY[+79.966331]YK(+3)	21.51	26.51	404.513	310.176132	21.7
GVHHIDY[+79.966331]YK(+3)	21.51	26.51	404.513	528.220949	15.7
GVHHIDY[+79.966331]YK(+3)	21.51	26.51	404.513	459.691493	17.7
GVHHIDY[+79.966331]YK (heavy)(+3)	21.51	26.51	407.184	676.246934	17.7
GVHHIDY[+79.966331]YK (heavy)(+3)	21.51	26.51	407.184	318.190331	21.7
GVHHIDY[+79.966331]YK (heavy)(+3)	21.51	26.51	407.184	532.228049	15.7
GVHHIDY[+79.966331]YK (heavy)(+3)	21.51	26.51	407.184	463.698593	17.7
GVHHIDYY[+79.966331]K(+3)	21.51	26.51	404.513	390.142463	21.7
GVHHIDYY[+79.966331]K(+3)	21.51	26.51	404.513	528.220949	15.7
GVHHIDYY[+79.966331]K(+3)	21.51	26.51	404.513	459.691493	16.7
GVHHIDYY[+79.966331]K (heavy)(+3)	21.51	26.51	407.184	398.156662	21.7
GVHHIDYY[+79.966331]K (heavy)(+3)	21.51	26.51	407.184	532.228049	15.7
GVHHIDYY[+79.966331]K (heavy)(+3)	21.51	26.51	407.184	463.698593	16.7
GVHHIDY[+79.966331]Y[+79.966331]K(+3)	19.25	24.25	431.168	748.199066	18.7
GVHHIDY[+79.966331]Y[+79.966331]K(+3)	19.25	24.25	431.168	633.172123	19.7
GVHHIDY[+79.966331]Y[+79.966331]K(+3)	19.25	24.25	431.168	499.674659	18.7
GVHHIDY[+79.966331]Y[+79.966331]K (heavy)(+3)	19.25	24.25	433.839	756.213265	18.7
GVHHIDY[+79.966331]Y[+79.966331]K (heavy)(+3)	19.25	24.25	433.839	641.186322	19.7
GVHHIDY[+79.966331]Y[+79.966331]K (heavy)(+3)	19.25	24.25	433.839	503.681758	18.7
GYLS[+79.966331]EGLVTK(+2)	57.41	62.41	573.775	715.398481	23.1
GYLS[+79.966331]EGLVTK(+2)	57.41	62.41	573.775	248.160482	17.1
GYLS[+79.966331]EGLVTK(+2)	57.41	62.41	573.775	221.092068	21.1
GYLS[+79.966331]EGLVTK (heavy)(+2)	57.41	62.41	577.782	723.41268	23.1
GYLS[+79.966331]EGLVTK (heavy)(+2)	57.41	62.41	577.782	256.174681	17.1
GYLS[+79.966331]EGLVTK (heavy)(+2)	57.41	62.41	577.782	221.092068	21.1
HADAEM[+15.994915]TGY[+79.966331]VVTR(+2)	27.36	32.36	773.323	774.354581	28.1
HADAEM[+15.994915]TGY[+79.966331]VVTR(+2)	27.36	32.36	773.323	276.16663	33.1
HADAEM[+15.994915]TGY[+79.966331]VVTR(+2)	27.36	32.36	773.323	209.103302	33.1
HADAEM[+15.994915]TGY[+79.966331]VVTR (heavy)(+2)	27.36	32.36	778.327	784.36285	28.1
HADAEM[+15.994915]TGY[+79.966331]VVTR (heavy)(+2)	27.36	32.36	778.327	286.174899	33.1
HADAEM[+15.994915]TGY[+79.966331]VVTR (heavy)(+2)	27.36	32.36	778.327	209.103302	33.1
HADAEMTGY[+79.966331]VVTR(+2)	34.55	39.55	765.326	774.354581	29.9
HADAEMTGY[+79.966331]VVTR(+2)	34.55	39.55	765.326	209.103302	31.9
HADAEMTGY[+79.966331]VVTR(+2)	34.55	39.55	765.326	324.130245	31.9
HADAEMTGY[+79.966331]VVTR (heavy)(+2)	34.55	39.55	770.33	784.36285	29.9
HADAEMTGY[+79.966331]VVTR (heavy)(+2)	34.55	39.55	770.33	209.103302	31.9
HADAEMTGY[+79.966331]VVTR (heavy)(+2)	34.55	39.55	770.33	324.130245	31.9
HADAEM[+15.994915]T[+79.966331]GY[+79.966331]VVTR(+2)	26.7	31.7	813.306	774.354581	34.3
HADAEM[+15.994915]T[+79.966331]GY[+79.966331]VVTR(+2)	26.7	31.7	813.306	276.16663	34.3
HADAEM[+15.994915]T[+79.966331]GY[+79.966331]VVTR(+2)	26.7	31.7	813.306	209.103302	34.3
HADAEM[+15.994915]T[+79.966331]GY[+79.966331]VVTR (heavy)(+2)	26.7	31.7	818.311	784.36285	34.3

HADAEM[+15.994915]T[+79.966331]GY[+79.966331]VVTR (heavy)(+2)	26.7	31.7	818.311	286.174899	34.3
HADAEM[+15.994915]T[+79.966331]GY[+79.966331]VVTR (heavy)(+2)	26.7	31.7	818.311	209.103302	34.3
HADAEMT[+79.966331]GY[+79.966331]VVTR(+2)	38.71	43.71	805.309	988.43218	31.1
HADAEMT[+79.966331]GY[+79.966331]VVTR(+2)	38.71	43.71	805.309	774.354581	28.1
HADAEMT[+79.966331]GY[+79.966331]VVTR(+2)	38.71	43.71	805.309	209.103302	31.1
HADAEMT[+79.966331]GY[+79.966331]VVTR (heavy)(+2)	38.71	43.71	810.313	998.440449	31.1
HADAEMT[+79.966331]GY[+79.966331]VVTR (heavy)(+2)	38.71	43.71	810.313	784.36285	28.1
HADAEMT[+79.966331]GY[+79.966331]VVTR (heavy)(+2)	38.71	43.71	810.313	209.103302	31.1
HADAEM[+15.994915]T[+79.966331]GYVVTR(+2)	28.02	33.02	773.323	694.38825	28.1
HADAEM[+15.994915]T[+79.966331]GYVVTR(+2)	28.02	33.02	773.323	375.235044	29.1
HADAEM[+15.994915]T[+79.966331]GYVVTR(+2)	28.02	33.02	773.323	209.103302	30.1
HADAEM[+15.994915]T[+79.966331]GYVVTR (heavy)(+2)	28.02	33.02	778.327	704.396519	28.1
HADAEM[+15.994915]T[+79.966331]GYVVTR (heavy)(+2)	28.02	33.02	778.327	385.243313	29.1
HADAEM[+15.994915]T[+79.966331]GYVVTR (heavy)(+2)	28.02	33.02	778.327	209.103302	30.1
HADAEMT[+79.966331]GYVVTR(+2)	39.49	44.49	765.326	1108.545556	32.9
HADAEMT[+79.966331]GYVVTR(+2)	39.49	44.49	765.326	694.38825	29.9
HADAEMT[+79.966331]GYVVTR(+2)	39.49	44.49	765.326	209.103302	29.9
HADAEMT[+79.966331]GYVVTR (heavy)(+2)	39.49	44.49	770.33	1118.553825	32.9
HADAEMT[+79.966331]GYVVTR (heavy)(+2)	39.49	44.49	770.33	704.396519	29.9
HADAEMT[+79.966331]GYVVTR (heavy)(+2)	39.49	44.49	770.33	209.103302	29.9
HM[+15.994915]T[+79.966331]QEVVTQYYR(+3)	38.47	43.47	550.904	829.420279	20.2
HM[+15.994915]T[+79.966331]QEVVTQYYR(+3)	38.47	43.47	550.904	730.351865	20.2
HM[+15.994915]T[+79.966331]QEVVTQYYR(+3)	38.47	43.47	550.904	723.216768	21.2
HM[+15.994915]T[+79.966331]QEVVTQYYR (heavy)(+3)	38.47	43.47	554.24	839.428548	20.2
HM[+15.994915]T[+79.966331]QEVVTQYYR (heavy)(+3)	38.47	43.47	554.24	740.360134	20.2
HM[+15.994915]T[+79.966331]QEVVTQYYR (heavy)(+3)	38.47	43.47	554.24	723.216768	21.2
HMT[+79.966331]QEVVTQYYR(+3)	44.47	49.47	545.572	730.351865	20
HMT[+79.966331]QEVVTQYYR(+3)	44.47	49.47	545.572	501.245609	20
HMT[+79.966331]QEVVTQYYR(+3)	44.47	49.47	545.572	707.221853	20
HMT[+79.966331]QEVVTQYYR (heavy)(+3)	44.47	49.47	548.908	740.360134	20
HMT[+79.966331]QEVVTQYYR (heavy)(+3)	44.47	49.47	548.908	511.253878	20
HMT[+79.966331]QEVVTQYYR (heavy)(+3)	44.47	49.47	548.908	707.221853	20
HTDDEM[+15.994915]T[+79.966331]GYVATR(+2)	22.96	27.96	796.308	666.35695	34.8
HTDDEM[+15.994915]T[+79.966331]GYVATR(+2)	22.96	27.96	796.308	347.203744	30.8
HTDDEM[+15.994915]T[+79.966331]GYVATR(+2)	22.96	27.96	796.308	239.113866	29.8
HTDDEM[+15.994915]T[+79.966331]GYVATR (heavy)(+2)	22.96	27.96	801.312	676.365219	34.8
HTDDEM[+15.994915]T[+79.966331]GYVATR (heavy)(+2)	22.96	27.96	801.312	357.212013	30.8
HTDDEM[+15.994915]T[+79.966331]GYVATR (heavy)(+2)	22.96	27.96	801.312	239.113866	29.8
HTDDEMT[+79.966331]GYVATR(+3)	35.58	40.58	525.876	446.272158	20.3
HTDDEMT[+79.966331]GYVATR(+3)	35.58	40.58	525.876	347.203744	20.3
HTDDEMT[+79.966331]GYVATR(+3)	35.58	40.58	525.876	435.158342	20.3

HTDDEMT[+79.966331]GYVATR (heavy)(+3)	35.58	40.58	529.212	456.280427	20.3
HTDDEMT[+79.966331]GYVATR (heavy)(+3)	35.58	40.58	529.212	357.212013	20.3
HTDDEMT[+79.966331]GYVATR (heavy)(+3)	35.58	40.58	529.212	435.158342	20.3
HTDDEM[+15.994915]T[+79.966331]GY[+79.966331]VATR(+2)	21.23	26.23	836.291	746.323281	35
HTDDEM[+15.994915]T[+79.966331]GY[+79.966331]VATR(+2)	21.23	26.23	836.291	446.272158	27
HTDDEM[+15.994915]T[+79.966331]GY[+79.966331]VATR(+2)	21.23	26.23	836.291	239.113866	35
HTDDEM[+15.994915]T[+79.966331]GY[+79.966331]VATR (heavy)(+2)	21.23	26.23	841.295	756.33155	35
HTDDEM[+15.994915]T[+79.966331]GY[+79.966331]VATR (heavy)(+2)	21.23	26.23	841.295	456.280427	27
HTDDEM[+15.994915]T[+79.966331]GY[+79.966331]VATR (heavy)(+2)	21.23	26.23	841.295	239.113866	35
HTDDEMT[+79.966331]GY[+79.966331]VATR(+3)	33.12	38.12	552.531	446.272158	20.3
HTDDEMT[+79.966331]GY[+79.966331]VATR(+3)	33.12	38.12	552.531	347.203744	22.3
HTDDEMT[+79.966331]GY[+79.966331]VATR(+3)	33.12	38.12	552.531	598.210346	20.3
HTDDEMT[+79.966331]GY[+79.966331]VATR (heavy)(+3)	33.12	38.12	555.867	456.280427	20.3
HTDDEMT[+79.966331]GY[+79.966331]VATR (heavy)(+3)	33.12	38.12	555.867	357.212013	22.3
HTDDEMT[+79.966331]GY[+79.966331]VATR (heavy)(+3)	33.12	38.12	555.867	598.210346	20.3
HTDDEM[+15.994915]TGY[+79.966331]VATR(+2)	19.36	24.36	796.308	746.323281	33.8
HTDDEM[+15.994915]TGY[+79.966331]VATR(+2)	19.36	24.36	796.308	347.203744	34.8
HTDDEM[+15.994915]TGY[+79.966331]VATR(+2)	19.36	24.36	796.308	239.113866	29.8
HTDDEM[+15.994915]TGY[+79.966331]VATR (heavy)(+2)	19.36	24.36	801.312	756.33155	33.8
HTDDEM[+15.994915]TGY[+79.966331]VATR (heavy)(+2)	19.36	24.36	801.312	357.212013	34.8
HTDDEM[+15.994915]TGY[+79.966331]VATR (heavy)(+2)	19.36	24.36	801.312	239.113866	29.8
HTDDEMTGY[+79.966331]VATR(+3)	29.8	34.8	525.876	746.323281	19.3
HTDDEMTGY[+79.966331]VATR(+3)	29.8	34.8	525.876	598.210346	20.3
HTDDEMTGY[+79.966331]VATR(+3)	29.8	34.8	525.876	729.25083	19.3
HTDDEMTGY[+79.966331]VATR (heavy)(+3)	29.8	34.8	529.212	756.33155	19.3
HTDDEMTGY[+79.966331]VATR (heavy)(+3)	29.8	34.8	529.212	598.210346	20.3
HTDDEMTGY[+79.966331]VATR (heavy)(+3)	29.8	34.8	529.212	729.25083	19.3
IADLGLAS[+79.966331]FK(+2)	68.47	73.47	557.78	832.45633	22.6
IADLGLAS[+79.966331]FK(+2)	68.47	73.47	557.78	604.345323	23.6
IADLGLAS[+79.966331]FK(+2)	68.47	73.47	557.78	300.155397	22.6
IADLGLAS[+79.966331]FK (heavy)(+2)	68.47	73.47	561.787	840.470529	22.6
IADLGLAS[+79.966331]FK (heavy)(+2)	68.47	73.47	561.787	612.359522	23.6
IADLGLAS[+79.966331]FK (heavy)(+2)	68.47	73.47	561.787	300.155397	22.6
IADPEHDHTGFLT[+79.966331]EYVATR(+3)	61.32	66.32	751.338	821.415193	32.8
IADPEHDHTGFLT[+79.966331]EYVATR(+3)	61.32	66.32	751.338	609.335487	32.8
IADPEHDHTGFLT[+79.966331]EYVATR(+3)	61.32	66.32	751.338	347.203744	33.8
IADPEHDHTGFLT[+79.966331]EYVATR (heavy)(+3)	61.32	66.32	754.674	831.423462	32.8
IADPEHDHTGFLT[+79.966331]EYVATR (heavy)(+3)	61.32	66.32	754.674	619.343756	32.8
IADPEHDHTGFLT[+79.966331]EYVATR (heavy)(+3)	61.32	66.32	754.674	357.212013	33.8
IADPEHDHTGFLTEY[+79.966331]VATR(+3)	57.6	62.6	751.338	689.301818	30.8
IADPEHDHTGFLTEY[+79.966331]VATR(+3)	57.6	62.6	751.338	347.203744	33.8

IADPEHDHTGFLTEY[+79.966331]VATR(+3)	57.6	62.6	751.338	300.155397	28.8
IADPEHDHTGFLTEY[+79.966331]VATR (heavy)(+3)	57.6	62.6	754.674	699.310087	30.8
IADPEHDHTGFLTEY[+79.966331]VATR (heavy)(+3)	57.6	62.6	754.674	357.212013	33.8
IADPEHDHTGFLTEY[+79.966331]VATR (heavy)(+3)	57.6	62.6	754.674	300.155397	28.8
IADPEHDHT[+79.966331]GFLTEYVATR(+3)	57.6	62.6	751.338	839.425758	27.8
IADPEHDHT[+79.966331]GFLTEYVATR(+3)	57.6	62.6	751.338	609.335487	28.8
IADPEHDHT[+79.966331]GFLTEYVATR(+3)	57.6	62.6	751.338	300.155397	27.8
IADPEHDHT[+79.966331]GFLTEYVATR (heavy)(+3)	57.6	62.6	754.674	849.434027	27.8
IADPEHDHT[+79.966331]GFLTEYVATR (heavy)(+3)	57.6	62.6	754.674	619.343756	28.8
IADPEHDHT[+79.966331]GFLTEYVATR (heavy)(+3)	57.6	62.6	754.674	300.155397	27.8
IC[+57.021464]DFGAS[+79.966331]R(+2)	35.79	40.79	503.196	794.324998	21
IC[+57.021464]DFGAS[+79.966331]R(+2)	35.79	40.79	503.196	634.29435	20
IC[+57.021464]DFGAS[+79.966331]R(+2)	35.79	40.79	503.196	519.267407	23
IC[+57.021464]DFGAS[+79.966331]R (heavy)(+2)	35.79	40.79	508.2	804.333267	21
IC[+57.021464]DFGAS[+79.966331]R (heavy)(+2)	35.79	40.79	508.2	644.302619	20
IC[+57.021464]DFGAS[+79.966331]R (heavy)(+2)	35.79	40.79	508.2	529.275676	23
IDQGDLM[+15.994915]T[+79.966331]PQFTPYYVAPQVLEAQR(+3)	88.59	93.59	992.799	1011.558169	32
IDQGDLM[+15.994915]T[+79.966331]PQFTPYYVAPQVLEAQR(+3)	88.59	93.59	992.799	940.521056	36
IDQGDLM[+15.994915]T[+79.966331]PQFTPYYVAPQVLEAQR(+3)	88.59	93.59	992.799	616.3413	38
IDQGDLM[+15.994915]T[+79.966331]PQFTPYYVAPQVLEAQR (heavy)(+3)	88.59	93.59	996.135	1021.566438	32
IDQGDLM[+15.994915]T[+79.966331]PQFTPYYVAPQVLEAQR (heavy)(+3)	88.59	93.59	996.135	950.529325	36
IDQGDLM[+15.994915]T[+79.966331]PQFTPYYVAPQVLEAQR (heavy)(+3)	88.59	93.59	996.135	626.349569	38
IDQGDLMT[+79.966331]PQFTPYYVAPQVLEAQR(+3)	93.92	98.92	987.468	1011.558169	35.8
IDQGDLMT[+79.966331]PQFTPYYVAPQVLEAQR(+3)	93.92	98.92	987.468	940.521056	36.8
IDQGDLMT[+79.966331]PQFTPYYVAPQVLEAQR(+3)	93.92	98.92	987.468	616.3413	38.8
IDQGDLMT[+79.966331]PQFTPYYVAPQVLEAQR (heavy)(+3)	93.92	98.92	990.804	1021.566438	35.8
IDQGDLMT[+79.966331]PQFTPYYVAPQVLEAQR (heavy)(+3)	93.92	98.92	990.804	950.529325	36.8
IDQGDLMT[+79.966331]PQFTPYYVAPQVLEAQR (heavy)(+3)	93.92	98.92	990.804	626.349569	38.8
IGDFGLAT[+79.966331]VK(+2)	61.01	66.01	550.773	570.360973	22.4
IGDFGLAT[+79.966331]VK(+2)	61.01	66.01	550.773	400.255445	22.4
IGDFGLAT[+79.966331]VK(+2)	61.01	66.01	550.773	286.139747	21.4
IGDFGLAT[+79.966331]VK (heavy)(+2)	61.01	66.01	554.78	578.375172	22.4
IGDFGLAT[+79.966331]VK (heavy)(+2)	61.01	66.01	554.78	408.269644	22.4
IGDFGLAT[+79.966331]VK (heavy)(+2)	61.01	66.01	554.78	286.139747	21.4
IIDSEY[+79.966331]TAQEGAK(+2)	31.62	36.62	752.831	947.387004	22.5
IIDSEY[+79.966331]TAQEGAK(+2)	31.62	36.62	752.831	704.357344	24.5
IIDSEY[+79.966331]TAQEGAK(+2)	31.62	36.62	752.831	342.202347	26.5
IIDSEY[+79.966331]TAQEGAK (heavy)(+2)	31.62	36.62	756.839	955.401203	22.5
IIDSEY[+79.966331]TAQEGAK (heavy)(+2)	31.62	36.62	756.839	712.371543	24.5
IIDSEY[+79.966331]TAQEGAK (heavy)(+2)	31.62	36.62	756.839	342.202347	26.5
IYNGDYY[+79.966331]R(+2)	32.76	37.76	572.228	867.303274	18.1

IYNGDYY[+79.966331]R(+2)	32.76	37.76	572.228	418.148611	22.1
IYNGDYY[+79.966331]R(+2)	32.76	37.76	572.228	277.154669	18.1
IYNGDYY[+79.966331]R (heavy)(+2)	32.76	37.76	577.233	877.311543	18.1
IYNGDYY[+79.966331]R (heavy)(+2)	32.76	37.76	577.233	428.15688	22.1
IYNGDYY[+79.966331]R (heavy)(+2)	32.76	37.76	577.233	277.154669	18.1
IVDFGS[+79.966331]SC[+57.021464]QLGQR(+2)	54.14	59.14	773.839	848.404312	26.1
IVDFGS[+79.966331]SC[+57.021464]QLGQR(+2)	54.14	59.14	773.839	360.198993	27.1
IVDFGS[+79.966331]SC[+57.021464]QLGQR(+2)	54.14	59.14	773.839	328.186697	29.1
IVDFGS[+79.966331]SC[+57.021464]QLGQR (heavy)(+2)	54.14	59.14	778.843	858.412581	26.1
IVDFGS[+79.966331]SC[+57.021464]QLGQR (heavy)(+2)	54.14	59.14	778.843	370.207262	27.1
IVDFGS[+79.966331]SC[+57.021464]QLGQR (heavy)(+2)	54.14	59.14	778.843	328.186697	29.1
IY[+79.966331]QY[+79.966331]IQSR(+2)	36.23	41.23	615.751	390.209558	24.4
IY[+79.966331]QY[+79.966331]IQSR(+2)	36.23	41.23	615.751	262.15098	24.4
IY[+79.966331]QY[+79.966331]IQSR(+2)	36.23	41.23	615.751	357.121	23.4
IY[+79.966331]QY[+79.966331]IQSR (heavy)(+2)	36.23	41.23	620.755	400.217827	24.4
IY[+79.966331]QY[+79.966331]IQSR (heavy)(+2)	36.23	41.23	620.755	272.159249	24.4
IY[+79.966331]QY[+79.966331]IQSR (heavy)(+2)	36.23	41.23	620.755	357.121	23.4
IYQY[+79.966331]IQSR(+2)	34.89	39.89	575.768	746.323281	19.2
IYQY[+79.966331]IQSR(+2)	34.89	39.89	575.768	503.293622	21.2
IYQY[+79.966331]IQSR(+2)	34.89	39.89	575.768	390.209558	21.2
IYQY[+79.966331]IQSR (heavy)(+2)	34.89	39.89	580.772	756.33155	19.2
IYQY[+79.966331]IQSR (heavy)(+2)	34.89	39.89	580.772	513.301891	21.2
IYQY[+79.966331]IQSR (heavy)(+2)	34.89	39.89	580.772	400.217827	21.2
IYSGDY[+79.966331]YR(+2)	34.88	39.88	558.723	840.292375	18.7
IYSGDY[+79.966331]YR(+2)	34.88	39.88	558.723	338.18228	21.7
IYSGDY[+79.966331]YR(+2)	34.88	39.88	558.723	277.154669	15.7
IYSGDY[+79.966331]YR (heavy)(+2)	34.88	39.88	563.727	850.300644	18.7
IYSGDY[+79.966331]YR (heavy)(+2)	34.88	39.88	563.727	348.190549	21.7
IYSGDY[+79.966331]YR (heavy)(+2)	34.88	39.88	563.727	277.154669	15.7
IYSGDYY[+79.966331]R(+2)	33.95	38.95	558.723	840.292375	15.7
IYSGDYY[+79.966331]R(+2)	33.95	38.95	558.723	418.148611	24.7
IYSGDYY[+79.966331]R(+2)	33.95	38.95	558.723	277.154669	17.7
IYSGDYY[+79.966331]R (heavy)(+2)	33.95	38.95	563.727	850.300644	15.7
IYSGDYY[+79.966331]R (heavy)(+2)	33.95	38.95	563.727	428.15688	24.7
IYSGDYY[+79.966331]R (heavy)(+2)	33.95	38.95	563.727	277.154669	17.7
IY[+79.966331]SGDYYR(+2)	35.41	40.41	558.723	1003.355704	19.7
IY[+79.966331]SGDYYR(+2)	35.41	40.41	558.723	760.326044	20.7
IY[+79.966331]SGDYYR(+2)	35.41	40.41	558.723	357.121	20.7
IY[+79.966331]SGDYYR (heavy)(+2)	35.41	40.41	563.727	1013.363973	19.7
IY[+79.966331]SGDYYR (heavy)(+2)	35.41	40.41	563.727	770.334313	20.7
IY[+79.966331]SGDYYR (heavy)(+2)	35.41	40.41	563.727	357.121	20.7

LADFGS[+79.966331]C[+57.021464]LK(+2)	54.75	59.75	545.735	808.3658	19.3
LADFGS[+79.966331]C[+57.021464]LK(+2)	54.75	59.75	545.735	546.270443	22.3
LADFGS[+79.966331]C[+57.021464]LK(+2)	54.75	59.75	545.735	300.155397	19.3
LADFGS[+79.966331]C[+57.021464]LK (heavy)(+2)	54.75	59.75	549.742	816.379999	19.3
LADFGS[+79.966331]C[+57.021464]LK (heavy)(+2)	54.75	59.75	549.742	554.284642	22.3
LADFGS[+79.966331]C[+57.021464]LK (heavy)(+2)	54.75	59.75	549.742	300.155397	19.3
LAVVGS[+79.966331]PFWM[+15.994915]APEVLR(+2)	92.84	97.84	934.462	684.403901	32.9
LAVVGS[+79.966331]PFWM[+15.994915]APEVLR(+2)	92.84	97.84	934.462	613.366787	29.9
LAVVGS[+79.966331]PFWM[+15.994915]APEVLR(+2)	92.84	97.84	934.462	284.196868	36.9
LAVVGS[+79.966331]PFWM[+15.994915]APEVLR (heavy)(+2)	92.84	97.84	939.466	694.41217	32.9
LAVVGS[+79.966331]PFWM[+15.994915]APEVLR (heavy)(+2)	92.84	97.84	939.466	623.375056	29.9
LAVVGS[+79.966331]PFWM[+15.994915]APEVLR (heavy)(+2)	92.84	97.84	939.466	284.196868	36.9
LAVVGS[+79.966331]PFWMAPEVLR(+2)	101.64	106.64	926.464	1245.644876	36.7
LAVVGS[+79.966331]PFWMAPEVLR(+2)	101.64	106.64	926.464	815.444385	30.7
LAVVGS[+79.966331]PFWMAPEVLR(+2)	101.64	106.64	926.464	684.403901	26.7
LAVVGS[+79.966331]PFWMAPEVLR (heavy)(+2)	101.64	106.64	931.469	1255.653145	36.7
LAVVGS[+79.966331]PFWMAPEVLR (heavy)(+2)	101.64	106.64	931.469	825.452654	30.7
LAVVGS[+79.966331]PFWMAPEVLR (heavy)(+2)	101.64	106.64	931.469	694.41217	26.7
LC[+57.021464]DFGISGQLVDS[+79.966331]IAK(+2)	78.96	83.96	901.923	515.282388	35
LC[+57.021464]DFGISGQLVDS[+79.966331]IAK(+2)	78.96	83.96	901.923	274.121989	33
LC[+57.021464]DFGISGQLVDS[+79.966331]IAK(+2)	78.96	83.96	901.923	389.148932	36
LC[+57.021464]DFGISGQLVDS[+79.966331]IAK (heavy)(+2)	78.96	83.96	905.93	523.296587	35
LC[+57.021464]DFGISGQLVDS[+79.966331]IAK (heavy)(+2)	78.96	83.96	905.93	274.121989	33
LC[+57.021464]DFGISGQLVDS[+79.966331]IAK (heavy)(+2)	78.96	83.96	905.93	389.148932	36
LADFGS[+79.966331]C[+57.021464]LR(+2)	55.95	60.95	559.738	836.371948	19.7
LADFGS[+79.966331]C[+57.021464]LR(+2)	55.95	60.95	559.738	574.276591	23.7
LADFGS[+79.966331]C[+57.021464]LR(+2)	55.95	60.95	559.738	846.381451	19.7
LADFGS[+79.966331]C[+57.021464]LR (heavy)(+2)	55.95	60.95	564.742	846.380217	19.7
LADFGS[+79.966331]C[+57.021464]LR (heavy)(+2)	55.95	60.95	564.742	584.28486	23.7
LADFGS[+79.966331]C[+57.021464]LR (heavy)(+2)	55.95	60.95	564.742	846.381451	19.7
LC[+57.021464]DFGVSGQLIDS[+79.966331]M[+15.994915]ANSFVGTR(+3)	82.26	87.26	824.03	1182.520798	30.6
LC[+57.021464]DFGVSGQLIDS[+79.966331]M[+15.994915]ANSFVGTR(+3)	82.26	87.26	824.03	851.436992	31.6
LC[+57.021464]DFGVSGQLIDS[+79.966331]M[+15.994915]ANSFVGTR(+3)	82.26	87.26	824.03	666.35695	29.6
LC[+57.021464]DFGVSGQLIDS[+79.966331]M[+15.994915]ANSFVGTR(+3)	82.26	87.26	824.03	432.256508	32.6
LC[+57.021464]DFGVSGQLIDS[+79.966331]M[+15.994915]ANSFVGTR (heavy)(+3)	82.26	87.26	827.366	1192.529067	30.6
LC[+57.021464]DFGVSGQLIDS[+79.966331]M[+15.994915]ANSFVGTR (heavy)(+3)	82.26	87.26	827.366	861.445261	31.6
LC[+57.021464]DFGVSGQLIDS[+79.966331]M[+15.994915]ANSFVGTR (heavy)(+3)	82.26	87.26	827.366	676.365219	29.6
LC[+57.021464]DFGVSGQLIDS[+79.966331]M[+15.994915]ANSFVGTR (heavy)(+3)	82.26	87.26	827.366	442.264777	32.6
IADFGFSNEFT[+79.966331]VGNK(+2)	77.72	82.72	863.379	500.282722	31.8
IADFGFSNEFT[+79.966331]VGNK(+2)	77.72	82.72	863.379	318.177195	26.8
IADFGFSNEFT[+79.966331]VGNK(+2)	77.72	82.72	863.379	300.155397	32.8

IADFGFSNEFT[+79.966331]VGNK (heavy)(+2)	77.72	82.72	867.386	508.296921	31.8
IADFGFSNEFT[+79.966331]VGNK (heavy)(+2)	77.72	82.72	867.386	326.191394	26.8
IADFGFSNEFT[+79.966331]VGNK (heavy)(+2)	77.72	82.72	867.386	300.155397	32.8
LDTFC[+57.021464]GS[+79.966331]PPYAAPELFQGK(+3)	81.71	86.71	726.658	818.44068	26.9
LDTFC[+57.021464]GS[+79.966331]PPYAAPELFQGK(+3)	81.71	86.71	726.658	592.345323	27.9
LDTFC[+57.021464]GS[+79.966331]PPYAAPELFQGK(+3)	81.71	86.71	726.658	763.307951	22.9
LDTFC[+57.021464]GS[+79.966331]PPYAAPELFQGK (heavy)(+3)	81.71	86.71	729.33	826.454879	26.9
LDTFC[+57.021464]GS[+79.966331]PPYAAPELFQGK (heavy)(+3)	81.71	86.71	729.33	600.359522	27.9
LDTFC[+57.021464]GS[+79.966331]PPYAAPELFQGK (heavy)(+3)	81.71	86.71	729.33	763.307951	22.9
LDT[+79.966331]FC[+57.021464]GSPPYAAPELFQGK(+3)	81.71	86.71	726.658	960.514908	20.9
LDT[+79.966331]FC[+57.021464]GSPPYAAPELFQGK(+3)	81.71	86.71	726.658	332.192845	35.9
LDT[+79.966331]FC[+57.021464]GSPPYAAPELFQGK(+3)	81.71	86.71	726.658	409.723978	21.9
LDT[+79.966331]FC[+57.021464]GSPPYAAPELFQGK (heavy)(+3)	81.71	86.71	729.33	968.529107	20.9
LDT[+79.966331]FC[+57.021464]GSPPYAAPELFQGK (heavy)(+3)	81.71	86.71	729.33	340.207044	35.9
LDT[+79.966331]FC[+57.021464]GSPPYAAPELFQGK (heavy)(+3)	81.71	86.71	729.33	413.731077	21.9
LDT[+79.966331]FC[+57.021464]GS[+79.966331]PPYAAPELFQGK(+3)	93.04	98.04	753.314	818.44068	21.9
LDT[+79.966331]FC[+57.021464]GS[+79.966331]PPYAAPELFQGK(+3)	93.04	98.04	753.314	592.345323	27.9
LDT[+79.966331]FC[+57.021464]GS[+79.966331]PPYAAPELFQGK(+3)	93.04	98.04	753.314	843.274282	26.9
LDT[+79.966331]FC[+57.021464]GS[+79.966331]PPYAAPELFQGK (heavy)(+3)	93.04	98.04	755.985	826.454879	21.9
LDT[+79.966331]FC[+57.021464]GS[+79.966331]PPYAAPELFQGK (heavy)(+3)	93.04	98.04	755.985	600.359522	27.9
LDT[+79.966331]FC[+57.021464]GS[+79.966331]PPYAAPELFQGK (heavy)(+3)	93.04	98.04	755.985	843.274282	26.9
LM[+15.994915]TGDT[+79.966331]YTAHAGAK(+3)	25.69	30.69	511.553	655.352199	21.7
LM[+15.994915]TGDT[+79.966331]YTAHAGAK(+3)	25.69	30.69	511.553	587.778001	19.7
LM[+15.994915]TGDT[+79.966331]YTAHAGAK(+3)	25.69	30.69	511.553	451.229959	19.7
LM[+15.994915]TGDT[+79.966331]YTAHAGAK (heavy)(+3)	25.69	30.69	514.224	663.366398	21.7
LM[+15.994915]TGDT[+79.966331]YTAHAGAK (heavy)(+3)	25.69	30.69	514.224	591.785101	19.7
LM[+15.994915]TGDT[+79.966331]YTAHAGAK (heavy)(+3)	25.69	30.69	514.224	455.237058	19.7
LMTGDT[+79.966331]YTAHAGAK(+3)	24.08	29.08	506.221	655.352199	21.5
LMTGDT[+79.966331]YTAHAGAK(+3)	24.08	29.08	506.221	636.766449	18.5
LMTGDT[+79.966331]YTAHAGAK(+3)	24.08	29.08	506.221	245.131825	25.5
LMTGDT[+79.966331]YTAHAGAK (heavy)(+3)	24.08	29.08	508.892	663.366398	21.5
LMTGDT[+79.966331]YTAHAGAK (heavy)(+3)	24.08	29.08	508.892	640.773549	18.5
LMTGDT[+79.966331]YTAHAGAK (heavy)(+3)	24.08	29.08	508.892	245.131825	25.5
LM[+15.994915]TGDTY[+79.966331]TAHAGAK(+3)	19.95	24.95	511.553	898.381859	21.7
LM[+15.994915]TGDTY[+79.966331]TAHAGAK(+3)	19.95	24.95	511.553	655.352199	22.7
LM[+15.994915]TGDTY[+79.966331]TAHAGAK(+3)	19.95	24.95	511.553	346.208495	23.7
LM[+15.994915]TGDTY[+79.966331]TAHAGAK(+3)	19.95	24.95	511.553	636.766449	18.7
LM[+15.994915]TGDTY[+79.966331]TAHAGAK (heavy)(+3)	19.95	24.95	514.224	906.396058	21.7
LM[+15.994915]TGDTY[+79.966331]TAHAGAK (heavy)(+3)	19.95	24.95	514.224	663.366398	22.7
LM[+15.994915]TGDTY[+79.966331]TAHAGAK (heavy)(+3)	19.95	24.95	514.224	354.222694	23.7
LM[+15.994915]TGDTY[+79.966331]TAHAGAK (heavy)(+3)	19.95	24.95	514.224	640.773549	18.7

LMTGDTY[+79.966331]TAHAGAK(+3)	32.08	37.08	506.221	655.352199	21.5
LMTGDTY[+79.966331]TAHAGAK(+3)	32.08	37.08	506.221	636.766449	18.5
LMTGDTY[+79.966331]TAHAGAK(+3)	32.08	37.08	506.221	245.131825	25.5
LMTGDTY[+79.966331]TAHAGAK (heavy)(+3)	32.08	37.08	508.892	663.366398	21.5
LMTGDTY[+79.966331]TAHAGAK (heavy)(+3)	32.08	37.08	508.892	640.773549	18.5
LMTGDTY[+79.966331]TAHAGAK (heavy)(+3)	32.08	37.08	508.892	245.131825	25.5
LM[+15.994915]TGDTYT[+79.966331]AHAGAK(+3)	24.62	29.62	511.553	800.404963	21.7
LM[+15.994915]TGDTYT[+79.966331]AHAGAK(+3)	24.62	29.62	511.553	637.341634	20.7
LM[+15.994915]TGDTYT[+79.966331]AHAGAK(+3)	24.62	29.62	511.553	483.267407	21.7
LM[+15.994915]TGDTYT[+79.966331]AHAGAK (heavy)(+3)	24.62	29.62	514.224	808.419162	21.7
LM[+15.994915]TGDTYT[+79.966331]AHAGAK (heavy)(+3)	24.62	29.62	514.224	645.355833	20.7
LM[+15.994915]TGDTYT[+79.966331]AHAGAK (heavy)(+3)	24.62	29.62	514.224	491.281606	21.7
LMTGDTYT[+79.966331]AHAGAK(+3)	29.4	34.4	506.221	800.404963	22.5
LMTGDTYT[+79.966331]AHAGAK(+3)	29.4	34.4	506.221	637.341634	21.5
LMTGDTYT[+79.966331]AHAGAK(+3)	29.4	34.4	506.221	483.267407	21.5
LMTGDTYT[+79.966331]AHAGAK (heavy)(+3)	29.4	34.4	508.892	808.419162	22.5
LMTGDTYT[+79.966331]AHAGAK (heavy)(+3)	29.4	34.4	508.892	645.355833	21.5
LMTGDTYT[+79.966331]AHAGAK (heavy)(+3)	29.4	34.4	508.892	491.281606	21.5
LYTY[+79.966331]IQSR(+2)	38.62	43.62	562.262	847.37096	18.8
LYTY[+79.966331]IQSR(+2)	38.62	43.62	562.262	746.323281	19.8
LYTY[+79.966331]IQSR(+2)	38.62	43.62	562.262	503.293622	23.8
LYTY[+79.966331]IQSR (heavy)(+2)	38.62	43.62	567.266	857.379229	18.8
LYTY[+79.966331]IQSR (heavy)(+2)	38.62	43.62	567.266	756.33155	19.8
LYTY[+79.966331]IQSR (heavy)(+2)	38.62	43.62	567.266	513.301891	23.8
MMSLS[+79.966331]QSR(+2)	37.27	42.27	510.206	790.387598	21.2
MMSLS[+79.966331]QSR(+2)	37.27	42.27	510.206	659.347114	20.2
MMSLS[+79.966331]QSR(+2)	37.27	42.27	510.206	459.231021	25.2
MMSLS[+79.966331]QSR(+2)	37.27	42.27	510.206	262.15098	20.2
MMSLS[+79.966331]QSR (heavy)(+2)	37.27	42.27	515.21	800.395867	21.2
MMSLS[+79.966331]QSR (heavy)(+2)	37.27	42.27	515.21	669.355383	20.2
MMSLS[+79.966331]QSR (heavy)(+2)	37.27	42.27	515.21	469.23929	25.2
MMSLS[+79.966331]QSR (heavy)(+2)	37.27	42.27	515.21	272.159249	20.2
M[+15.994915]M[+15.994915]SLS[+79.966331]QSR(+2)	18.47	23.47	526.201	659.347114	21.7
M[+15.994915]M[+15.994915]SLS[+79.966331]QSR(+2)	18.47	23.47	526.201	572.315085	26.7
M[+15.994915]M[+15.994915]SLS[+79.966331]QSR(+2)	18.47	23.47	526.201	459.231021	25.7
M[+15.994915]M[+15.994915]SLS[+79.966331]QSR (heavy)(+2)	18.47	23.47	531.205	669.355383	21.7
M[+15.994915]M[+15.994915]SLS[+79.966331]QSR (heavy)(+2)	18.47	23.47	531.205	582.323354	26.7
M[+15.994915]M[+15.994915]SLS[+79.966331]QSR (heavy)(+2)	18.47	23.47	531.205	469.23929	25.7
MS[+79.966331]AAGTYAWMAPEVIR(+2)	84.43	89.43	917.406	1001.523698	29.4
MS[+79.966331]AAGTYAWMAPEVIR(+2)	84.43	89.43	917.406	684.403901	27.4
MS[+79.966331]AAGTYAWMAPEVIR(+2)	84.43	89.43	917.406	613.366787	28.4

MS[+79.966331]AAGTYAWMAPEVIR (heavy)(+2)	84.43	89.43	922.41	1011.531967	29.4
MS[+79.966331]AAGTYAWMAPEVIR (heavy)(+2)	84.43	89.43	922.41	694.41217	27.4
MS[+79.966331]AAGTYAWMAPEVIR (heavy)(+2)	84.43	89.43	922.41	623.375056	28.4
MS[+79.966331]AAGTYAWM[+15.994915]APEVIR(+2)	73.5	78.5	925.404	1017.518613	30.7
MS[+79.966331]AAGTYAWM[+15.994915]APEVIR(+2)	73.5	78.5	925.404	684.403901	30.7
MS[+79.966331]AAGTYAWM[+15.994915]APEVIR(+2)	73.5	78.5	925.404	613.366787	30.7
MS[+79.966331]AAGTYAWM[+15.994915]APEVIR (heavy)(+2)	73.5	78.5	930.408	1027.526882	30.7
MS[+79.966331]AAGTYAWM[+15.994915]APEVIR (heavy)(+2)	73.5	78.5	930.408	694.41217	30.7
MS[+79.966331]AAGTYAWM[+15.994915]APEVIR (heavy)(+2)	73.5	78.5	930.408	623.375056	30.7
M[+15.994915]S[+79.966331]AAGTYAWMAPEVIR(+2)	80.18	85.18	925.404	1001.523698	30.7
M[+15.994915]S[+79.966331]AAGTYAWMAPEVIR(+2)	80.18	85.18	925.404	684.403901	30.7
M[+15.994915]S[+79.966331]AAGTYAWMAPEVIR(+2)	80.18	85.18	925.404	613.366787	30.7
M[+15.994915]S[+79.966331]AAGTYAWMAPEVIR (heavy)(+2)	80.18	85.18	930.408	1011.531967	30.7
M[+15.994915]S[+79.966331]AAGTYAWMAPEVIR (heavy)(+2)	80.18	85.18	930.408	694.41217	30.7
M[+15.994915]S[+79.966331]AAGTYAWMAPEVIR (heavy)(+2)	80.18	85.18	930.408	623.375056	30.7
M[+15.994915]S[+79.966331]AAGTYAWM[+15.994915]APEVIR(+2)	69.63	74.63	933.401	1017.518613	30.9
M[+15.994915]S[+79.966331]AAGTYAWM[+15.994915]APEVIR(+2)	69.63	74.63	933.401	684.403901	30.9
M[+15.994915]S[+79.966331]AAGTYAWM[+15.994915]APEVIR(+2)	69.63	74.63	933.401	613.366787	30.9
M[+15.994915]S[+79.966331]AAGTYAWM[+15.994915]APEVIR (heavy)(+2)	69.63	74.63	938.405	1027.526882	30.9
M[+15.994915]S[+79.966331]AAGTYAWM[+15.994915]APEVIR (heavy)(+2)	69.63	74.63	938.405	694.41217	30.9
M[+15.994915]S[+79.966331]AAGTYAWM[+15.994915]APEVIR (heavy)(+2)	69.63	74.63	938.405	623.375056	30.9
MSAAGT[+79.966331]YAWMAPEVIR(+2)	81.64	86.64	917.406	1318.661254	30.4
MSAAGT[+79.966331]YAWMAPEVIR(+2)	81.64	86.64	917.406	1001.523698	28.4
MSAAGT[+79.966331]YAWMAPEVIR(+2)	81.64	86.64	917.406	684.403901	26.4
MSAAGT[+79.966331]YAWMAPEVIR(+2)	81.64	86.64	917.406	613.366787	29.4
MSAAGT[+79.966331]YAWMAPEVIR (heavy)(+2)	81.64	86.64	922.41	1328.669523	30.4
MSAAGT[+79.966331]YAWMAPEVIR (heavy)(+2)	81.64	86.64	922.41	1011.531967	28.4
MSAAGT[+79.966331]YAWMAPEVIR (heavy)(+2)	81.64	86.64	922.41	694.41217	26.4
MSAAGT[+79.966331]YAWMAPEVIR (heavy)(+2)	81.64	86.64	922.41	623.375056	29.4
M[+15.994915]SAAGT[+79.966331]YAWMAPEVIR(+2)	80.18	85.18	925.404	1318.661254	30.7
M[+15.994915]SAAGT[+79.966331]YAWMAPEVIR(+2)	80.18	85.18	925.404	1001.523698	30.7
M[+15.994915]SAAGT[+79.966331]YAWMAPEVIR(+2)	80.18	85.18	925.404	684.403901	30.7
M[+15.994915]SAAGT[+79.966331]YAWMAPEVIR(+2)	80.18	85.18	925.404	613.366787	30.7
M[+15.994915]SAAGT[+79.966331]YAWMAPEVIR (heavy)(+2)	80.18	85.18	930.408	1328.669523	30.7
M[+15.994915]SAAGT[+79.966331]YAWMAPEVIR (heavy)(+2)	80.18	85.18	930.408	1011.531967	30.7
M[+15.994915]SAAGT[+79.966331]YAWMAPEVIR (heavy)(+2)	80.18	85.18	930.408	694.41217	30.7
M[+15.994915]SAAGT[+79.966331]YAWMAPEVIR (heavy)(+2)	80.18	85.18	930.408	623.375056	30.7
M[+15.994915]SAAGT[+79.966331]YAWM[+15.994915]APEVIR(+2)	67.9	72.9	933.401	1334.656169	30.9
M[+15.994915]SAAGT[+79.966331]YAWM[+15.994915]APEVIR(+2)	67.9	72.9	933.401	1017.518613	30.9
M[+15.994915]SAAGT[+79.966331]YAWM[+15.994915]APEVIR(+2)	67.9	72.9	933.401	684.403901	30.9
M[+15.994915]SAAGT[+79.966331]YAWM[+15.994915]APEVIR(+2)	67.9	72.9	933.401	613.366787	30.9

M[+15.994915]SAAGT[+79.966331]YAWM[+15.994915]APEVIR (heavy)(+2)	67.9	72.9	938.405	1344.664438	30.9
M[+15.994915]SAAGT[+79.966331]YAWM[+15.994915]APEVIR (heavy)(+2)	67.9	72.9	938.405	1027.526882	30.9
M[+15.994915]SAAGT[+79.966331]YAWM[+15.994915]APEVIR (heavy)(+2)	67.9	72.9	938.405	694.41217	30.9
M[+15.994915]SAAGT[+79.966331]YAWM[+15.994915]APEVIR (heavy)(+2)	67.9	72.9	938.405	623.375056	30.9
MSAAGT[+79.966331]YAWM[+15.994915]APEVIR(+2)	71.49	76.49	925.404	1334.656169	30.7
MSAAGT[+79.966331]YAWM[+15.994915]APEVIR(+2)	71.49	76.49	925.404	1017.518613	30.7
MSAAGT[+79.966331]YAWM[+15.994915]APEVIR(+2)	71.49	76.49	925.404	684.403901	30.7
MSAAGT[+79.966331]YAWM[+15.994915]APEVIR(+2)	71.49	76.49	925.404	613.366787	30.7
MSAAGT[+79.966331]YAWM[+15.994915]APEVIR (heavy)(+2)	71.49	76.49	930.408	1344.664438	30.7
MSAAGT[+79.966331]YAWM[+15.994915]APEVIR (heavy)(+2)	71.49	76.49	930.408	1027.526882	30.7
MSAAGT[+79.966331]YAWM[+15.994915]APEVIR (heavy)(+2)	71.49	76.49	930.408	694.41217	30.7
MSAAGT[+79.966331]YAWM[+15.994915]APEVIR (heavy)(+2)	71.49	76.49	930.408	623.375056	30.7
M[+15.994915]S[+79.966331]TAGTYAWM[+15.994915]APEVIK(+2)	68.97	73.97	934.403	1060.549579	31.9
M[+15.994915]S[+79.966331]TAGTYAWM[+15.994915]APEVIK(+2)	68.97	73.97	934.403	656.397753	30.9
M[+15.994915]S[+79.966331]TAGTYAWM[+15.994915]APEVIK(+2)	68.97	73.97	934.403	585.360639	32.9
M[+15.994915]S[+79.966331]TAGTYAWM[+15.994915]APEVIK (heavy)(+2)	68.97	73.97	938.411	1068.563778	31.9
M[+15.994915]S[+79.966331]TAGTYAWM[+15.994915]APEVIK (heavy)(+2)	68.97	73.97	938.411	664.411952	30.9
M[+15.994915]S[+79.966331]TAGTYAWM[+15.994915]APEVIK (heavy)(+2)	68.97	73.97	938.411	593.374838	32.9
MS[+79.966331]TAGTYAWMAPEVIK(+2)	81.77	86.77	918.409	1044.554664	28.5
MS[+79.966331]TAGTYAWMAPEVIK(+2)	81.77	86.77	918.409	973.51755	30.5
MS[+79.966331]TAGTYAWMAPEVIK(+2)	81.77	86.77	918.409	656.397753	29.5
MS[+79.966331]TAGTYAWMAPEVIK(+2)	81.77	86.77	918.409	585.360639	33.5
MS[+79.966331]TAGTYAWMAPEVIK (heavy)(+2)	81.77	86.77	922.416	1052.568863	28.5
MS[+79.966331]TAGTYAWMAPEVIK (heavy)(+2)	81.77	86.77	922.416	981.531749	30.5
MS[+79.966331]TAGTYAWMAPEVIK (heavy)(+2)	81.77	86.77	922.416	664.411952	29.5
MS[+79.966331]TAGTYAWMAPEVIK (heavy)(+2)	81.77	86.77	922.416	593.374838	33.5
NDS[+79.966331]NYVVK(+2)	27.82	32.82	509.715	691.377351	18.2
NDS[+79.966331]NYVVK(+2)	27.82	32.82	509.715	345.249632	19.2
NDS[+79.966331]NYVVK(+2)	27.82	32.82	509.715	246.181218	20.2
NDS[+79.966331]NYVVK (heavy)(+2)	27.82	32.82	513.722	699.39155	18.2
NDS[+79.966331]NYVVK (heavy)(+2)	27.82	32.82	513.722	353.263831	19.2
NDS[+79.966331]NYVVK (heavy)(+2)	27.82	32.82	513.722	254.195417	20.2
NDSNY[+79.966331]VVK(+2)	20.88	25.88	509.715	789.354247	17.2
NDSNY[+79.966331]VVK(+2)	20.88	25.88	509.715	702.322219	19.2
NDSNY[+79.966331]VVK(+2)	20.88	25.88	509.715	246.181218	20.2
NDSNY[+79.966331]VVK (heavy)(+2)	20.88	25.88	513.722	797.368446	17.2
NDSNY[+79.966331]VVK (heavy)(+2)	20.88	25.88	513.722	710.336418	19.2
NDSNY[+79.966331]VVK (heavy)(+2)	20.88	25.88	513.722	254.195417	20.2
NDS[+79.966331]NY[+79.966331]VVK(+2)	30.36	35.36	549.698	771.343682	21.4
NDS[+79.966331]NY[+79.966331]VVK(+2)	30.36	35.36	549.698	702.322219	20.4
NDS[+79.966331]NY[+79.966331]VVK(+2)	30.36	35.36	549.698	246.181218	22.4

NDS[+79.966331]NY[+79.966331]VVK(+2)	30.36	35.36	549.698	147.112804	22.4
NDS[+79.966331]NY[+79.966331]VVK (heavy)(+2)	30.36	35.36	553.705	779.357881	21.4
NDS[+79.966331]NY[+79.966331]VVK (heavy)(+2)	30.36	35.36	553.705	710.336418	20.4
NDS[+79.966331]NY[+79.966331]VVK (heavy)(+2)	30.36	35.36	553.705	254.195417	22.4
NDS[+79.966331]NY[+79.966331]VVK (heavy)(+2)	30.36	35.36	553.705	155.127003	22.4
NIYSADY[+79.966331]YK(+2)	42.62	47.62	608.749	668.232735	19.2
NIYSADY[+79.966331]YK(+2)	42.62	47.62	608.749	553.205792	29.2
NIYSADY[+79.966331]YK(+2)	42.62	47.62	608.749	310.176132	29.2
NIYSADY[+79.966331]YK (heavy)(+2)	42.62	47.62	612.756	676.246934	19.2
NIYSADY[+79.966331]YK (heavy)(+2)	42.62	47.62	612.756	561.219991	29.2
NIYSADY[+79.966331]YK (heavy)(+2)	42.62	47.62	612.756	318.190331	29.2
NLYAGDY[+79.966331]YR(+2)	43.95	48.95	607.747	987.360789	17.1
NLYAGDY[+79.966331]YR(+2)	43.95	48.95	607.747	824.297461	19.1
NLYAGDY[+79.966331]YR(+2)	43.95	48.95	607.747	753.260347	17.1
NLYAGDY[+79.966331]YR (heavy)(+2)	43.95	48.95	612.751	997.369058	17.1
NLYAGDY[+79.966331]YR (heavy)(+2)	43.95	48.95	612.751	834.30573	19.1
NLYAGDY[+79.966331]YR (heavy)(+2)	43.95	48.95	612.751	763.268616	17.1
NLYAGDYY[+79.966331]R(+2)	43.42	48.42	607.747	987.360789	17.1
NLYAGDYY[+79.966331]R(+2)	43.42	48.42	607.747	824.297461	19.1
NLYAGDYY[+79.966331]R(+2)	43.42	48.42	607.747	753.260347	21.1
NLYAGDYY[+79.966331]R(+2)	43.42	48.42	607.747	418.148611	20.1
NLYAGDYY[+79.966331]R (heavy)(+2)	43.42	48.42	612.751	997.369058	17.1
NLYAGDYY[+79.966331]R (heavy)(+2)	43.42	48.42	612.751	834.30573	19.1
NLYAGDYY[+79.966331]R (heavy)(+2)	43.42	48.42	612.751	763.268616	21.1
NLYAGDYY[+79.966331]R (heavy)(+2)	43.42	48.42	612.751	428.15688	20.1
NLY[+79.966331]AGDYYR(+2)	45.16	50.16	607.747	987.360789	17.1
NLY[+79.966331]AGDYYR(+2)	45.16	50.16	607.747	744.33113	19.1
NLY[+79.966331]AGDYYR(+2)	45.16	50.16	607.747	228.134267	17.1
NLY[+79.966331]AGDYYR (heavy)(+2)	45.16	50.16	612.751	997.369058	17.1
NLY[+79.966331]AGDYYR (heavy)(+2)	45.16	50.16	612.751	754.339399	19.1
NLY[+79.966331]AGDYYR (heavy)(+2)	45.16	50.16	612.751	228.134267	17.1
NLYSGDY[+79.966331]YR(+2)	43.43	48.43	615.744	1003.355704	19.4
NLYSGDY[+79.966331]YR(+2)	43.43	48.43	615.744	840.292375	19.4
NLYSGDY[+79.966331]YR(+2)	43.43	48.43	615.744	228.134267	18.4
NLYSGDY[+79.966331]YR(+2)	43.43	48.43	615.744	391.197596	18.4
NLYSGDY[+79.966331]YR (heavy)(+2)	43.43	48.43	620.749	1013.363973	19.4
NLYSGDY[+79.966331]YR (heavy)(+2)	43.43	48.43	620.749	850.300644	19.4
NLYSGDY[+79.966331]YR (heavy)(+2)	43.43	48.43	620.749	228.134267	18.4
NLYSGDY[+79.966331]YR (heavy)(+2)	43.43	48.43	620.749	391.197596	18.4
NLYSGDYY[+79.966331]R(+2)	42.5	47.5	615.744	1003.355704	17.4
NLYSGDYY[+79.966331]R(+2)	42.5	47.5	615.744	840.292375	20.4

NLYSGDYY[+79.966331]R(+2)	42.5	47.5	615.744	418.148611	18.4
NLYSGDYY[+79.966331]R(+2)	42.5	47.5	615.744	228.134267	17.4
NLYSGDYY[+79.966331]R (heavy)(+2)	42.5	47.5	620.749	1013.363973	17.4
NLYSGDYY[+79.966331]R (heavy)(+2)	42.5	47.5	620.749	850.300644	20.4
NLYSGDYY[+79.966331]R (heavy)(+2)	42.5	47.5	620.749	428.15688	18.4
NLYSGDYY[+79.966331]R (heavy)(+2)	42.5	47.5	620.749	228.134267	17.4
NLY[+79.966331]SGDYYR(+2)	41.16	46.16	615.744	1003.355704	19.4
NLY[+79.966331]SGDYYR(+2)	41.16	46.16	615.744	760.326044	21.4
NLY[+79.966331]SGDYYR(+2)	41.16	46.16	615.744	501.245609	23.4
NLY[+79.966331]SGDYYR(+2)	41.16	46.16	615.744	228.134267	21.4
NLY[+79.966331]SGDYYR (heavy)(+2)	41.16	46.16	620.749	1013.363973	19.4
NLY[+79.966331]SGDYYR (heavy)(+2)	41.16	46.16	620.749	770.334313	21.4
NLY[+79.966331]SGDYYR (heavy)(+2)	41.16	46.16	620.749	511.253878	23.4
NLY[+79.966331]SGDYYR (heavy)(+2)	41.16	46.16	620.749	228.134267	21.4
NT[+79.966331]FVGTPFWM[+15.994915]APEVIK(+2)	88.72	93.72	966.951	1233.633643	28.9
NT[+79.966331]FVGTPFWM[+15.994915]APEVIK(+2)	88.72	93.72	966.951	345.155731	35.9
NT[+79.966331]FVGTPFWM[+15.994915]APEVIK(+2)	88.72	93.72	966.951	602.293287	31.9
NT[+79.966331]FVGTPFWM[+15.994915]APEVIK (heavy)(+2)	88.72	93.72	970.959	1241.647842	28.9
NT[+79.966331]FVGTPFWM[+15.994915]APEVIK (heavy)(+2)	88.72	93.72	970.959	345.155731	35.9
NT[+79.966331]FVGTPFWM[+15.994915]APEVIK (heavy)(+2)	88.72	93.72	970.959	602.293287	31.9
NT[+79.966331]FVGTPFWMAPEVIK(+2)	97.79	102.79	958.954	1217.638728	29.7
NT[+79.966331]FVGTPFWMAPEVIK(+2)	97.79	102.79	958.954	585.360639	34.7
NT[+79.966331]FVGTPFWMAPEVIK(+2)	97.79	102.79	958.954	345.155731	39.7
NT[+79.966331]FVGTPFWMAPEVIK (heavy)(+2)	97.79	102.79	962.961	1225.652927	29.7
NT[+79.966331]FVGTPFWMAPEVIK (heavy)(+2)	97.79	102.79	962.961	593.374838	34.7
NT[+79.966331]FVGTPFWMAPEVIK (heavy)(+2)	97.79	102.79	962.961	345.155731	39.7
PPYT[+79.966331]DY[+79.966331]VSTR(+2)	49.07	54.07	679.756	903.360789	30.3
PPYT[+79.966331]DY[+79.966331]VSTR(+2)	49.07	54.07	679.756	820.323675	26.3
PPYT[+79.966331]DY[+79.966331]VSTR(+2)	49.07	54.07	679.756	705.296732	30.3
PPYT[+79.966331]DY[+79.966331]VSTR(+2)	49.07	54.07	679.756	363.198659	29.3
PPYT[+79.966331]DY[+79.966331]VSTR (heavy)(+2)	49.07	54.07	684.761	913.369058	30.3
PPYT[+79.966331]DY[+79.966331]VSTR (heavy)(+2)	49.07	54.07	684.761	830.331944	26.3
PPYT[+79.966331]DY[+79.966331]VSTR (heavy)(+2)	49.07	54.07	684.761	715.305001	30.3
PPYT[+79.966331]DY[+79.966331]VSTR (heavy)(+2)	49.07	54.07	684.761	373.206928	29.3
PPYT[+79.966331]DYVSTR(+2)	48.11	53.11	639.773	823.394458	26.1
PPYT[+79.966331]DYVSTR(+2)	48.11	53.11	639.773	740.357344	22.1
PPYT[+79.966331]DYVSTR(+2)	48.11	53.11	639.773	625.330401	22.1
PPYT[+79.966331]DYVSTR(+2)	48.11	53.11	639.773	363.198659	22.1
PPYT[+79.966331]DYVSTR (heavy)(+2)	48.11	53.11	644.777	833.402727	26.1
PPYT[+79.966331]DYVSTR (heavy)(+2)	48.11	53.11	644.777	750.365613	22.1
PPYT[+79.966331]DYVSTR (heavy)(+2)	48.11	53.11	644.777	635.33867	22.1

PPYT[+79.966331]DYVSTR (heavy)(+2)	48.11	53.11	644.777	373.206928	22.1
PPYTDY[+79.966331]VSTR(+2)	39.85	44.85	639.773	1084.434682	25.1
PPYTDY[+79.966331]VSTR(+2)	39.85	44.85	639.773	921.371354	29.1
PPYTDY[+79.966331]VSTR(+2)	39.85	44.85	639.773	820.323675	28.1
PPYTDY[+79.966331]VSTR(+2)	39.85	44.85	639.773	705.296732	25.1
PPYTDY[+79.966331]VSTR (heavy)(+2)	39.85	44.85	644.777	1094.442951	25.1
PPYTDY[+79.966331]VSTR (heavy)(+2)	39.85	44.85	644.777	931.379623	29.1
PPYTDY[+79.966331]VSTR (heavy)(+2)	39.85	44.85	644.777	830.331944	28.1
PPYTDY[+79.966331]VSTR (heavy)(+2)	39.85	44.85	644.777	715.305001	25.1
PGEEDNAAISEVGT[+79.966331]IR(+2)	50.57	55.57	869.388	1025.502702	28
PGEEDNAAISEVGT[+79.966331]IR(+2)	50.57	55.57	869.388	954.465589	31
PGEEDNAAISEVGT[+79.966331]IR(+2)	50.57	55.57	869.388	841.381525	28
PGEEDNAAISEVGT[+79.966331]IR(+2)	50.57	55.57	869.388	526.238489	27
PGEEDNAAISEVGT[+79.966331]IR (heavy)(+2)	50.57	55.57	874.392	1035.510971	28
PGEEDNAAISEVGT[+79.966331]IR (heavy)(+2)	50.57	55.57	874.392	964.473858	31
PGEEDNAAISEVGT[+79.966331]IR (heavy)(+2)	50.57	55.57	874.392	851.389794	28
PGEEDNAAISEVGT[+79.966331]IR (heavy)(+2)	50.57	55.57	874.392	536.246758	27
QADEEM[+15.994915]T[+79.966331]GYVATR(+2)	30.97	35.97	783.81	749.394064	28.4
QADEEM[+15.994915]T[+79.966331]GYVATR(+2)	30.97	35.97	783.81	666.35695	30.4
QADEEM[+15.994915]T[+79.966331]GYVATR(+2)	30.97	35.97	783.81	446.272158	25.4
QADEEM[+15.994915]T[+79.966331]GYVATR(+2)	30.97	35.97	783.81	347.203744	25.4
QADEEM[+15.994915]T[+79.966331]GYVATR (heavy)(+2)	30.97	35.97	788.814	759.402333	28.4
QADEEM[+15.994915]T[+79.966331]GYVATR (heavy)(+2)	30.97	35.97	788.814	676.365219	30.4
QADEEM[+15.994915]T[+79.966331]GYVATR (heavy)(+2)	30.97	35.97	788.814	456.280427	25.4
QADEEM[+15.994915]T[+79.966331]GYVATR (heavy)(+2)	30.97	35.97	788.814	357.212013	25.4
QADEEMT[+79.966331]GYVATR(+2)	44.83	49.83	775.813	666.35695	28.2
QADEEMT[+79.966331]GYVATR(+2)	44.83	49.83	775.813	446.272158	26.2
QADEEMT[+79.966331]GYVATR(+2)	44.83	49.83	775.813	347.203744	27.2
QADEEMT[+79.966331]GYVATR (heavy)(+2)	44.83	49.83	780.817	676.365219	28.2
QADEEMT[+79.966331]GYVATR (heavy)(+2)	44.83	49.83	780.817	456.280427	26.2
QADEEMT[+79.966331]GYVATR (heavy)(+2)	44.83	49.83	780.817	357.212013	27.2
QADEEM[+15.994915]TGY[+79.966331]VATR(+2)	28.16	33.16	783.81	847.37096	28.4
QADEEM[+15.994915]TGY[+79.966331]VATR(+2)	28.16	33.16	783.81	746.323281	29.4
QADEEM[+15.994915]TGY[+79.966331]VATR(+2)	28.16	33.16	783.81	446.272158	26.4
QADEEM[+15.994915]TGY[+79.966331]VATR (heavy)(+2)	28.16	33.16	788.814	857.379229	28.4
QADEEM[+15.994915]TGY[+79.966331]VATR (heavy)(+2)	28.16	33.16	788.814	756.33155	29.4
QADEEM[+15.994915]TGY[+79.966331]VATR (heavy)(+2)	28.16	33.16	788.814	456.280427	26.4
QADEEMTGY[+79.966331]VATR(+2)	39.76	44.76	775.813	847.37096	27.2
QADEEMTGY[+79.966331]VATR(+2)	39.76	44.76	775.813	746.323281	26.2
QADEEMTGY[+79.966331]VATR(+2)	39.76	44.76	775.813	347.203744	27.2
QADEEMTGY[+79.966331]VATR (heavy)(+2)	39.76	44.76	780.817	857.379229	27.2

QADEEMTGY[+79.966331]VATR (heavy)(+2)	39.76	44.76	780.817	756.33155	26.2
QADEEMTGY[+79.966331]VATR (heavy)(+2)	39.76	44.76	780.817	357.212013	27.2
QADEEM[+15.994915]T[+79.966331]GY[+79.966331]VATR(+2)	28.04	33.04	823.793	829.360395	29.6
QADEEM[+15.994915]T[+79.966331]GY[+79.966331]VATR(+2)	28.04	33.04	823.793	746.323281	30.6
QADEEM[+15.994915]T[+79.966331]GY[+79.966331]VATR(+2)	28.04	33.04	823.793	347.203744	28.6
QADEEM[+15.994915]T[+79.966331]GY[+79.966331]VATR (heavy)(+2)	28.04	33.04	828.798	839.368664	29.6
QADEEM[+15.994915]T[+79.966331]GY[+79.966331]VATR (heavy)(+2)	28.04	33.04	828.798	756.33155	30.6
QADEEM[+15.994915]T[+79.966331]GY[+79.966331]VATR (heavy)(+2)	28.04	33.04	828.798	357.212013	28.6
QADEEMT[+79.966331]GY[+79.966331]VATR(+2)	45.23	50.23	815.796	829.360395	30.4
QADEEMT[+79.966331]GY[+79.966331]VATR(+2)	45.23	50.23	815.796	746.323281	27.4
QADEEMT[+79.966331]GY[+79.966331]VATR(+2)	45.23	50.23	815.796	446.272158	25.4
QADEEMT[+79.966331]GY[+79.966331]VATR (heavy)(+2)	45.23	50.23	820.8	839.368664	30.4
QADEEMT[+79.966331]GY[+79.966331]VATR (heavy)(+2)	45.23	50.23	820.8	756.33155	27.4
QADEEMT[+79.966331]GY[+79.966331]VATR (heavy)(+2)	45.23	50.23	820.8	456.280427	25.4
QADSEMT[+79.966331]GYVVTR(+2)	50.66	55.66	768.823	474.303458	26
QADSEMT[+79.966331]GYVVTR(+2)	50.66	55.66	768.823	375.235044	26
QADSEMT[+79.966331]GYVVTR(+2)	50.66	55.66	768.823	276.16663	28
QADSEMT[+79.966331]GYVVTR (heavy)(+2)	50.66	55.66	773.827	484.311727	26
QADSEMT[+79.966331]GYVVTR (heavy)(+2)	50.66	55.66	773.827	385.243313	26
QADSEMT[+79.966331]GYVVTR (heavy)(+2)	50.66	55.66	773.827	286.174899	28
QADSEMTGY[+79.966331]VVTR(+2)	41.36	46.36	768.823	1006.442745	26
QADSEMTGY[+79.966331]VVTR(+2)	41.36	46.36	768.823	875.40226	24
QADSEMTGY[+79.966331]VVTR(+2)	41.36	46.36	768.823	774.354581	24
QADSEMTGY[+79.966331]VVTR (heavy)(+2)	41.36	46.36	773.827	1016.451014	26
QADSEMTGY[+79.966331]VVTR (heavy)(+2)	41.36	46.36	773.827	885.410529	24
QADSEMTGY[+79.966331]VVTR (heavy)(+2)	41.36	46.36	773.827	784.36285	24
QADSEMT[+79.966331]GY[+79.966331]VVTR(+2)	47.37	52.37	808.806	857.391695	31.2
QADSEMT[+79.966331]GY[+79.966331]VVTR(+2)	47.37	52.37	808.806	774.354581	28.2
QADSEMT[+79.966331]GY[+79.966331]VVTR(+2)	47.37	52.37	808.806	474.303458	25.2
QADSEMT[+79.966331]GY[+79.966331]VVTR (heavy)(+2)	47.37	52.37	813.81	867.399964	31.2
QADSEMT[+79.966331]GY[+79.966331]VVTR (heavy)(+2)	47.37	52.37	813.81	784.36285	28.2
QADSEMT[+79.966331]GY[+79.966331]VVTR (heavy)(+2)	47.37	52.37	813.81	484.311727	25.2
QALT[+79.966331]LQDWAAQR(+2)	65.71	70.71	740.85	874.41659	24.1
QALT[+79.966331]LQDWAAQR(+2)	65.71	70.71	740.85	746.358013	25.1
QALT[+79.966331]LQDWAAQR(+2)	65.71	70.71	740.85	631.33107	28.1
QALT[+79.966331]LQDWAAQR (heavy)(+2)	65.71	70.71	745.854	884.424859	24.1
QALT[+79.966331]LQDWAAQR (heavy)(+2)	65.71	70.71	745.854	756.366282	25.1
QALT[+79.966331]LQDWAAQR (heavy)(+2)	65.71	70.71	745.854	641.339339	28.1
C[+57.021464]TIS[+79.966331]YR(+2)	35.39	40.39	440.175	621.335486	20.1
C[+57.021464]TIS[+79.966331]YR(+2)	35.39	40.39	440.175	520.287808	19.1
C[+57.021464]TIS[+79.966331]YR(+2)	35.39	40.39	440.175	407.203744	20.1

C[+57.021464]TIS[+79.966331]YR(+2)	35.39	40.39	440.175	338.18228	19.1
C[+57.021464]TIS[+79.966331]YR (heavy)(+2)	35.39	40.39	445.179	631.343755	20.1
C[+57.021464]TIS[+79.966331]YR (heavy)(+2)	35.39	40.39	445.179	530.296077	19.1
C[+57.021464]TIS[+79.966331]YR (heavy)(+2)	35.39	40.39	445.179	417.212013	20.1
C[+57.021464]TIS[+79.966331]YR (heavy)(+2)	35.39	40.39	445.179	348.190549	19.1
C[+57.021464]TISY[+79.966331]R(+2)	26.32	31.32	440.175	618.264704	17.1
C[+57.021464]TISY[+79.966331]R(+2)	26.32	31.32	440.175	505.18064	18.1
C[+57.021464]TISY[+79.966331]R(+2)	26.32	31.32	440.175	418.148611	15.1
C[+57.021464]TISY[+79.966331]R(+2)	26.32	31.32	440.175	262.085603	16.1
C[+57.021464]TISY[+79.966331]R (heavy)(+2)	26.32	31.32	445.179	628.272973	17.1
C[+57.021464]TISY[+79.966331]R (heavy)(+2)	26.32	31.32	445.179	515.188909	18.1
C[+57.021464]TISY[+79.966331]R (heavy)(+2)	26.32	31.32	445.179	428.15688	15.1
C[+57.021464]TISY[+79.966331]R (heavy)(+2)	26.32	31.32	445.179	262.085603	16.1
C[+57.021464]TIS[+79.966331]Y[+79.966331]R(+2)	30.46	35.46	480.158	600.254139	20.3
C[+57.021464]TIS[+79.966331]Y[+79.966331]R(+2)	30.46	35.46	480.158	487.170075	21.3
C[+57.021464]TIS[+79.966331]Y[+79.966331]R(+2)	30.46	35.46	480.158	262.085603	18.3
C[+57.021464]TIS[+79.966331]Y[+79.966331]R (heavy)(+2)	30.46	35.46	485.162	610.262408	20.3
C[+57.021464]TIS[+79.966331]Y[+79.966331]R (heavy)(+2)	30.46	35.46	485.162	497.178344	21.3
C[+57.021464]TIS[+79.966331]Y[+79.966331]R (heavy)(+2)	30.46	35.46	485.162	262.085603	18.3
QEDGGVY[+79.966331]SSSGLK(+2)	28.55	33.55	703.795	1149.482361	25
QEDGGVY[+79.966331]SSSGLK(+2)	28.55	33.55	703.795	821.344076	23
QEDGGVY[+79.966331]SSSGLK(+2)	28.55	33.55	703.795	578.314417	23
QEDGGVY[+79.966331]SSSGLK (heavy)(+2)	28.55	33.55	707.802	1157.49656	25
QEDGGVY[+79.966331]SSSGLK (heavy)(+2)	28.55	33.55	707.802	829.358275	23
QEDGGVY[+79.966331]SSSGLK (heavy)(+2)	28.55	33.55	707.802	586.328616	23
QET[+79.966331]VEC[+57.021464]LK(+2)	30.07	35.07	543.73	731.375637	22.2
QET[+79.966331]VEC[+57.021464]LK(+2)	30.07	35.07	543.73	549.270109	19.2
QET[+79.966331]VEC[+57.021464]LK(+2)	30.07	35.07	543.73	420.227516	21.2
QET[+79.966331]VEC[+57.021464]LK (heavy)(+2)	30.07	35.07	547.737	739.389836	22.2
QET[+79.966331]VEC[+57.021464]LK (heavy)(+2)	30.07	35.07	547.737	557.284308	19.2
QET[+79.966331]VEC[+57.021464]LK (heavy)(+2)	30.07	35.07	547.737	428.241715	21.2
QS[+79.966331]GVVVEEPPPSK(+2)	35.75	40.75	716.839	882.456724	25.4
QS[+79.966331]GVVVEEPPPSK(+2)	35.75	40.75	716.839	525.303124	25.4
QS[+79.966331]GVVVEEPPPSK(+2)	35.75	40.75	716.839	428.25036	28.4
QS[+79.966331]GVVVEEPPPSK (heavy)(+2)	35.75	40.75	720.846	890.470923	25.4
QS[+79.966331]GVVVEEPPPSK (heavy)(+2)	35.75	40.75	720.846	533.317323	25.4
QS[+79.966331]GVVVEEPPPSK (heavy)(+2)	35.75	40.75	720.846	436.264559	28.4
NSS[+79.966331]PAPPQPAPGK(+2)	23.2	28.2	664.305	791.441014	22.8
NSS[+79.966331]PAPPQPAPGK(+2)	23.2	28.2	664.305	301.187031	24.8
NSS[+79.966331]PAPPQPAPGK(+2)	23.2	28.2	664.305	396.224145	26.8
NSS[+79.966331]PAPPQPAPGK (heavy)(+2)	23.2	28.2	668.312	799.455213	22.8

NSS[+79.966331]PAPPQPAPGK (heavy)(+2)	23.2	28.2	668.312	309.20123	24.8
NSS[+79.966331]PAPPQPAPGK (heavy)(+2)	23.2	28.2	668.312	400.231245	26.8
S[+79.966331]LVGTPYWM[+15.994915]APELISR(+2)	86.32	91.32	958.454	785.451579	36.7
S[+79.966331]LVGTPYWM[+15.994915]APELISR(+2)	86.32	91.32	958.454	714.414465	39.7
S[+79.966331]LVGTPYWM[+15.994915]APELISR(+2)	86.32	91.32	958.454	282.181217	37.7
S[+79.966331]LVGTPYWM[+15.994915]APELISR (heavy)(+2)	86.32	91.32	963.458	795.459848	36.7
S[+79.966331]LVGTPYWM[+15.994915]APELISR (heavy)(+2)	86.32	91.32	963.458	724.422734	39.7
S[+79.966331]LVGTPYWM[+15.994915]APELISR (heavy)(+2)	86.32	91.32	963.458	282.181217	37.7
S[+79.966331]LVGTPYWMAPELISR(+2)	95.39	100.39	950.457	785.451579	31.4
S[+79.966331]LVGTPYWMAPELISR(+2)	95.39	100.39	950.457	714.414465	33.4
S[+79.966331]LVGTPYWMAPELISR(+2)	95.39	100.39	950.457	380.158113	30.4
S[+79.966331]LVGTPYWMAPELISR (heavy)(+2)	95.39	100.39	955.461	795.459848	31.4
S[+79.966331]LVGTPYWMAPELISR (heavy)(+2)	95.39	100.39	955.461	724.422734	33.4
S[+79.966331]LVGTPYWMAPELISR (heavy)(+2)	95.39	100.39	955.461	380.158113	30.4
S[+79.966331]LVGTPYWM[+15.994915]APEVISR(+2)	80.72	85.72	951.446	1300.668449	40.4
S[+79.966331]LVGTPYWM[+15.994915]APEVISR(+2)	80.72	85.72	951.446	700.398815	36.4
S[+79.966331]LVGTPYWM[+15.994915]APEVISR(+2)	80.72	85.72	951.446	282.181217	40.4
S[+79.966331]LVGTPYWM[+15.994915]APEVISR (heavy)(+2)	80.72	85.72	956.451	1310.676718	40.4
S[+79.966331]LVGTPYWM[+15.994915]APEVISR (heavy)(+2)	80.72	85.72	956.451	710.407084	36.4
S[+79.966331]LVGTPYWM[+15.994915]APEVISR (heavy)(+2)	80.72	85.72	956.451	282.181217	40.4
S[+79.966331]LVGTPYWMAPEVISR(+2)	89.78	94.78	943.449	902.476414	32.2
S[+79.966331]LVGTPYWMAPEVISR(+2)	89.78	94.78	943.449	700.398815	31.2
S[+79.966331]LVGTPYWMAPEVISR(+2)	89.78	94.78	943.449	282.181217	35.2
S[+79.966331]LVGTPYWMAPEVISR (heavy)(+2)	89.78	94.78	948.453	912.484683	32.2
S[+79.966331]LVGTPYWMAPEVISR (heavy)(+2)	89.78	94.78	948.453	710.407084	31.2
S[+79.966331]LVGTPYWMAPEVISR (heavy)(+2)	89.78	94.78	948.453	282.181217	35.2
S[+79.966331]VVGTPAYLAPEVLR(+2)	76.39	81.39	826.426	1128.641171	25.7
S[+79.966331]VVGTPAYLAPEVLR(+2)	76.39	81.39	826.426	684.403901	26.7
S[+79.966331]VVGTPAYLAPEVLR(+2)	76.39	81.39	826.426	613.366787	24.7
S[+79.966331]VVGTPAYLAPEVLR(+2)	76.39	81.39	826.426	268.165567	31.7
S[+79.966331]VVGTPAYLAPEVLR (heavy)(+2)	76.39	81.39	831.43	1138.64944	25.7
S[+79.966331]VVGTPAYLAPEVLR (heavy)(+2)	76.39	81.39	831.43	694.41217	26.7
S[+79.966331]VVGTPAYLAPEVLR (heavy)(+2)	76.39	81.39	831.43	623.375056	24.7
S[+79.966331]VVGTPAYLAPEVLR (heavy)(+2)	76.39	81.39	831.43	268.165567	31.7
GEEVY[+79.966331]VK(+2)	24.47	29.47	452.196	717.321884	14.5
GEEVY[+79.966331]VK(+2)	24.47	29.47	452.196	588.279291	15.5
GEEVY[+79.966331]VK(+2)	24.47	29.47	452.196	489.210877	18.5
GEEVY[+79.966331]VK(+2)	24.47	29.47	452.196	187.071333	15.5
GEEVY[+79.966331]VK (heavy)(+2)	24.47	29.47	456.203	725.336083	14.5
GEEVY[+79.966331]VK (heavy)(+2)	24.47	29.47	456.203	596.29349	15.5
GEEVY[+79.966331]VK (heavy)(+2)	24.47	29.47	456.203	497.225076	18.5

GEEVY[+79.966331]VK (heavy)(+2)	24.47	29.47	456.203	187.071333	15.5
SDPSGHLT[+79.966331]GMVGTALYVSPEVQGSTK(+3)	69.24	74.24	900.085	1031.536765	33.5
SDPSGHLT[+79.966331]GMVGTALYVSPEVQGSTK(+3)	69.24	74.24	900.085	932.468351	32.5
SDPSGHLT[+79.966331]GMVGTALYVSPEVQGSTK(+3)	69.24	74.24	900.085	845.436323	32.5
SDPSGHLT[+79.966331]GMVGTALYVSPEVQGSTK(+3)	69.24	74.24	900.085	1063.391437	36.5
SDPSGHLT[+79.966331]GMVGTALYVSPEVQGSTK (heavy)(+3)	69.24	74.24	902.757	1039.550964	33.5
SDPSGHLT[+79.966331]GMVGTALYVSPEVQGSTK (heavy)(+3)	69.24	74.24	902.757	940.48255	32.5
SDPSGHLT[+79.966331]GMVGTALYVSPEVQGSTK (heavy)(+3)	69.24	74.24	902.757	853.450522	32.5
SDPSGHLT[+79.966331]GMVGTALYVSPEVQGSTK (heavy)(+3)	69.24	74.24	902.757	1063.391437	36.5
SDPSGHLT[+79.966331]GM[+15.994915]VGTALYVSPEVQGSTK(+3)	57.65	62.65	905.417	1031.536765	30.7
SDPSGHLT[+79.966331]GM[+15.994915]VGTALYVSPEVQGSTK(+3)	57.65	62.65	905.417	932.468351	28.7
SDPSGHLT[+79.966331]GM[+15.994915]VGTALYVSPEVQGSTK(+3)	57.65	62.65	905.417	845.436323	33.7
SDPSGHLT[+79.966331]GM[+15.994915]VGTALYVSPEVQGSTK (heavy)(+3)	57.65	62.65	908.088	1039.550964	30.7
SDPSGHLT[+79.966331]GM[+15.994915]VGTALYVSPEVQGSTK (heavy)(+3)	57.65	62.65	908.088	940.48255	28.7
SDPSGHLT[+79.966331]GM[+15.994915]VGTALYVSPEVQGSTK (heavy)(+3)	57.65	62.65	908.088	853.450522	33.7
SDPSGHLTGMVGT[+79.966331]ALYVSPEVQGSTK(+3)	65.09	70.09	900.085	1031.536765	32.5
SDPSGHLTGMVGT[+79.966331]ALYVSPEVQGSTK(+3)	65.09	70.09	900.085	932.468351	29.5
SDPSGHLTGMVGT[+79.966331]ALYVSPEVQGSTK(+3)	65.09	70.09	900.085	845.436323	29.5
SDPSGHLTGMVGT[+79.966331]ALYVSPEVQGSTK (heavy)(+3)	65.09	70.09	902.757	1039.550964	32.5
SDPSGHLTGMVGT[+79.966331]ALYVSPEVQGSTK (heavy)(+3)	65.09	70.09	902.757	940.48255	29.5
SDPSGHLTGMVGT[+79.966331]ALYVSPEVQGSTK (heavy)(+3)	65.09	70.09	902.757	853.450522	29.5
SDPSGHLTGM[+15.994915]VGT[+79.966331]ALYVSPEVQGSTK(+3)	57.65	62.65	905.417	1194.600094	28.7
SDPSGHLTGM[+15.994915]VGT[+79.966331]ALYVSPEVQGSTK(+3)	57.65	62.65	905.417	932.468351	28.7
SDPSGHLTGM[+15.994915]VGT[+79.966331]ALYVSPEVQGSTK(+3)	57.65	62.65	905.417	845.436323	28.7
SDPSGHLTGM[+15.994915]VGT[+79.966331]ALYVSPEVQGSTK (heavy)(+3)	57.65	62.65	908.088	1202.614293	28.7
SDPSGHLTGM[+15.994915]VGT[+79.966331]ALYVSPEVQGSTK (heavy)(+3)	57.65	62.65	908.088	940.48255	28.7
SDPSGHLTGM[+15.994915]VGT[+79.966331]ALYVSPEVQGSTK (heavy)(+3)	57.65	62.65	908.088	853.450522	28.7
SEIGHSPPPAY[+79.966331]TPMSGNQFVYR(+3)	55.9	60.9	839.041	1198.567354	30.2
SEIGHSPPPAY[+79.966331]TPMSGNQFVYR(+3)	55.9	60.9	839.041	970.474105	33.2
SEIGHSPPPAY[+79.966331]TPMSGNQFVYR(+3)	55.9	60.9	839.041	883.442077	30.2
SEIGHSPPPAY[+79.966331]TPMSGNQFVYR (heavy)(+3)	55.9	60.9	842.378	1208.575623	30.2
SEIGHSPPPAY[+79.966331]TPMSGNQFVYR (heavy)(+3)	55.9	60.9	842.378	980.482374	33.2
SEIGHSPPPAY[+79.966331]TPMSGNQFVYR (heavy)(+3)	55.9	60.9	842.378	893.450346	30.2
SFGS[+79.966331]PNR(+2)	20.59	25.59	422.671	610.234466	14.6
SFGS[+79.966331]PNR(+2)	20.59	25.59	422.671	512.25757	19.6
SFGS[+79.966331]PNR(+2)	20.59	25.59	422.671	235.107718	14.6
SFGS[+79.966331]PNR (heavy)(+2)	20.59	25.59	427.675	620.242735	14.6
SFGS[+79.966331]PNR (heavy)(+2)	20.59	25.59	427.675	522.265839	19.6
SFGS[+79.966331]PNR (heavy)(+2)	20.59	25.59	427.675	235.107718	14.6
AYT[+79.966331]HQVVTR(+2)	21.17	26.17	577.771	822.458061	23.2
AYT[+79.966331]HQVVTR(+2)	21.17	26.17	577.771	739.420948	23.2

AYT[+79.966331]HQVVTR(+2)	21.17	26.17	577.771	602.362036	26.2
AYT[+79.966331]HQVVTR(+2)	21.17	26.17	577.771	474.303458	25.2
AYT[+79.966331]HQVVTR (heavy)(+2)	21.17	26.17	582.775	832.46633	23.2
AYT[+79.966331]HQVVTR (heavy)(+2)	21.17	26.17	582.775	749.429217	23.2
AYT[+79.966331]HQVVTR (heavy)(+2)	21.17	26.17	582.775	612.370305	26.2
AYT[+79.966331]HQVVTR (heavy)(+2)	21.17	26.17	582.775	484.311727	25.2
C[+57.021464]LTSNLLQS[+79.966331]R(+2)	51.97	56.97	636.294	900.489755	25
C[+57.021464]LTSNLLQS[+79.966331]R(+2)	51.97	56.97	636.294	485.283057	25
C[+57.021464]LTSNLLQS[+79.966331]R(+2)	51.97	56.97	636.294	274.121989	22
C[+57.021464]LTSNLLQS[+79.966331]R (heavy)(+2)	51.97	56.97	641.298	910.498024	25
C[+57.021464]LTSNLLQS[+79.966331]R (heavy)(+2)	51.97	56.97	641.298	495.291326	25
C[+57.021464]LTSNLLQS[+79.966331]R (heavy)(+2)	51.97	56.97	641.298	274.121989	22
DYLSSS[+79.966331]FLC[+57.021464]SDDDR(+2)	74.27	79.27	880.329	1096.436399	30.3
DYLSSS[+79.966331]FLC[+57.021464]SDDDR(+2)	74.27	79.27	880.329	767.262458	27.3
DYLSSS[+79.966331]FLC[+57.021464]SDDDR(+2)	74.27	79.27	880.329	290.145895	38.3
DYLSSS[+79.966331]FLC[+57.021464]SDDDR(+2)	74.27	79.27	880.329	279.097548	35.3
DYLSSS[+79.966331]FLC[+57.021464]SDDDR (heavy)(+2)	74.27	79.27	885.333	1106.444668	30.3
DYLSSS[+79.966331]FLC[+57.021464]SDDDR (heavy)(+2)	74.27	79.27	885.333	777.270727	27.3
DYLSSS[+79.966331]FLC[+57.021464]SDDDR (heavy)(+2)	74.27	79.27	885.333	300.154164	38.3
DYLSSS[+79.966331]FLC[+57.021464]SDDDR (heavy)(+2)	74.27	79.27	885.333	279.097548	35.3
DYLSSS[+79.966331]FLC[+57.021464]S[+79.966331]DDDR(+2)	84.7	89.7	920.312	749.251893	30.5
DYLSSS[+79.966331]FLC[+57.021464]S[+79.966331]DDDR(+2)	84.7	89.7	920.312	290.145895	30.5
DYLSSS[+79.966331]FLC[+57.021464]S[+79.966331]DDDR(+2)	84.7	89.7	920.312	279.097548	30.5
DYLSSS[+79.966331]FLC[+57.021464]S[+79.966331]DDDR (heavy)(+2)	84.7	89.7	925.316	759.260162	30.5
DYLSSS[+79.966331]FLC[+57.021464]S[+79.966331]DDDR (heavy)(+2)	84.7	89.7	925.316	300.154164	30.5
DYLSSS[+79.966331]FLC[+57.021464]S[+79.966331]DDDR (heavy)(+2)	84.7	89.7	925.316	279.097548	30.5
GVENPAVQES[+79.966331]NQK(+2)	25.35	30.35	740.327	1080.47213	22.1
GVENPAVQES[+79.966331]NQK(+2)	25.35	30.35	740.327	982.495234	27.1
GVENPAVQES[+79.966331]NQK(+2)	25.35	30.35	740.327	587.278365	28.1
GVENPAVQES[+79.966331]NQK(+2)	25.35	30.35	740.327	540.739703	26.1
GVENPAVQES[+79.966331]NQK (heavy)(+2)	25.35	30.35	744.334	1088.486329	22.1
GVENPAVQES[+79.966331]NQK (heavy)(+2)	25.35	30.35	744.334	990.509433	27.1
GVENPAVQES[+79.966331]NQK (heavy)(+2)	25.35	30.35	744.334	595.292564	28.1
GVENPAVQES[+79.966331]NQK (heavy)(+2)	25.35	30.35	744.334	544.746803	26.1
S[+79.966331]FNSHINASNNSEPSR(+3)	29.03	34.03	614.258	575.278366	22.6
S[+79.966331]FNSHINASNNSEPSR(+3)	29.03	34.03	614.258	488.246337	23.6
S[+79.966331]FNSHINASNNSEPSR(+3)	29.03	34.03	614.258	359.203744	25.6
S[+79.966331]FNSHINASNNSEPSR(+3)	29.03	34.03	614.258	331.140081	26.6
S[+79.966331]FNSHINASNNSEPSR (heavy)(+3)	29.03	34.03	617.594	585.286635	22.6
S[+79.966331]FNSHINASNNSEPSR (heavy)(+3)	29.03	34.03	617.594	498.254606	23.6
S[+79.966331]FNSHINASNNSEPSR (heavy)(+3)	29.03	34.03	617.594	369.212013	25.6

S[+79.966331]FNSHINASNNSEPSR (heavy)(+3)	29.03	34.03	617.594	331.140081	26.6
S[+79.966331]FNSHINASNNS[+79.966331]EPSR(+3)	33.71	38.71	640.913	671.310728	24.6
S[+79.966331]FNSHINASNNS[+79.966331]EPSR(+3)	33.71	38.71	640.913	557.267801	23.6
S[+79.966331]FNSHINASNNS[+79.966331]EPSR(+3)	33.71	38.71	640.913	359.203744	29.6
S[+79.966331]FNSHINASNNS[+79.966331]EPSR(+3)	33.71	38.71	640.913	331.140081	28.6
S[+79.966331]FNSHINASNNS[+79.966331]EPSR (heavy)(+3)	33.71	38.71	644.25	681.318997	24.6
S[+79.966331]FNSHINASNNS[+79.966331]EPSR (heavy)(+3)	33.71	38.71	644.25	567.27607	23.6
S[+79.966331]FNSHINASNNS[+79.966331]EPSR (heavy)(+3)	33.71	38.71	644.25	369.212013	29.6
S[+79.966331]FNSHINASNNS[+79.966331]EPSR (heavy)(+3)	33.71	38.71	644.25	331.140081	28.6
SFNSHINASNNS[+79.966331]EPSR(+3)	27.16	32.16	614.258	671.310728	24.6
SFNSHINASNNS[+79.966331]EPSR(+3)	27.16	32.16	614.258	557.267801	21.6
SFNSHINASNNS[+79.966331]EPSR(+3)	27.16	32.16	614.258	359.203744	24.6
SFNSHINASNNS[+79.966331]EPSR (heavy)(+3)	27.16	32.16	617.594	681.318997	24.6
SFNSHINASNNS[+79.966331]EPSR (heavy)(+3)	27.16	32.16	617.594	567.27607	21.6
SFNSHINASNNS[+79.966331]EPSR (heavy)(+3)	27.16	32.16	617.594	369.212013	24.6
ILGT[+79.966331]PDYLAPELLLGR(+2)	100.17	105.17	910.981	1144.672471	31.2
ILGT[+79.966331]PDYLAPELLLGR(+2)	100.17	105.17	910.981	868.525078	28.2
ILGT[+79.966331]PDYLAPELLLGR(+2)	100.17	105.17	910.981	797.487965	30.2
ILGT[+79.966331]PDYLAPELLLGR(+2)	100.17	105.17	910.981	367.233981	34.2
ILGT[+79.966331]PDYLAPELLLGR (heavy)(+2)	100.17	105.17	915.985	1154.68074	31.2
ILGT[+79.966331]PDYLAPELLLGR (heavy)(+2)	100.17	105.17	915.985	878.533347	28.2
ILGT[+79.966331]PDYLAPELLLGR (heavy)(+2)	100.17	105.17	915.985	807.496234	30.2
ILGT[+79.966331]PDYLAPELLLGR (heavy)(+2)	100.17	105.17	915.985	367.233981	34.2
SGEPLST[+79.966331]WC[+57.021464]GSPPYAAPEVFEGK(+3)	82.26	87.26	849.369	805.409046	25.6
SGEPLST[+79.966331]WC[+57.021464]GSPPYAAPEVFEGK(+3)	82.26	87.26	849.369	204.134267	36.6
SGEPLST[+79.966331]WC[+57.021464]GSPPYAAPEVFEGK(+3)	82.26	87.26	849.369	484.240189	26.6
SGEPLST[+79.966331]WC[+57.021464]GSPPYAAPEVFEGK (heavy)(+3)	82.26	87.26	852.041	813.423245	25.6
SGEPLST[+79.966331]WC[+57.021464]GSPPYAAPEVFEGK (heavy)(+3)	82.26	87.26	852.041	212.148466	36.6
SGEPLST[+79.966331]WC[+57.021464]GSPPYAAPEVFEGK (heavy)(+3)	82.26	87.26	852.041	484.240189	26.6
STMVGT[+79.966331]PYWMAPEVVTR(+2)	83.54	88.54	1002.951	902.476414	34
STMVGT[+79.966331]PYWMAPEVVTR(+2)	83.54	88.54	1002.951	771.435929	33
STMVGT[+79.966331]PYWMAPEVVTR(+2)	83.54	88.54	1002.951	700.398815	33
STMVGT[+79.966331]PYWMAPEVVTR (heavy)(+2)	83.54	88.54	1007.955	912.484683	34
STMVGT[+79.966331]PYWMAPEVVTR (heavy)(+2)	83.54	88.54	1007.955	781.444198	33
STMVGT[+79.966331]PYWMAPEVVTR (heavy)(+2)	83.54	88.54	1007.955	710.407084	33
SVVGTPAY[+79.966331]LAPEVLLNQGYNR(+3)	86.37	91.37	781.057	977.516305	24
SVVGTPAY[+79.966331]LAPEVLLNQGYNR(+3)	86.37	91.37	781.057	864.432241	29
SVVGTPAY[+79.966331]LAPEVLLNQGYNR(+3)	86.37	91.37	781.057	751.348177	24
SVVGTPAY[+79.966331]LAPEVLLNQGYNR (heavy)(+3)	86.37	91.37	784.393	987.524574	24
SVVGTPAY[+79.966331]LAPEVLLNQGYNR (heavy)(+3)	86.37	91.37	784.393	874.44051	29
SVVGTPAY[+79.966331]LAPEVLLNQGYNR (heavy)(+3)	86.37	91.37	784.393	761.356446	24

S[+79.966331]VVGTPAYLAPEVLLNQGYNR(+3)	90.64	95.64	781.057	864.432241	28
S[+79.966331]VVGTPAYLAPEVLLNQGYNR(+3)	90.64	95.64	781.057	751.348177	29
S[+79.966331]VVGTPAYLAPEVLLNQGYNR(+3)	90.64	95.64	781.057	509.246672	23
S[+79.966331]VVGTPAYLAPEVLLNQGYNR(+3)	90.64	95.64	781.057	325.187031	35
S[+79.966331]VVGTPAYLAPEVLLNQGYNR (heavy)(+3)	90.64	95.64	784.393	874.44051	28
S[+79.966331]VVGTPAYLAPEVLLNQGYNR (heavy)(+3)	90.64	95.64	784.393	761.356446	29
S[+79.966331]VVGTPAYLAPEVLLNQGYNR (heavy)(+3)	90.64	95.64	784.393	519.254941	23
S[+79.966331]VVGTPAYLAPEVLLNQGYNR (heavy)(+3)	90.64	95.64	784.393	325.187031	35
T[+79.966331]IC[+57.021464]GTPNYLSPEVLNK(+2)	69.24	74.24	943.441	699.403566	30.2
T[+79.966331]IC[+57.021464]GTPNYLSPEVLNK(+2)	69.24	74.24	943.441	374.239795	31.2
T[+79.966331]IC[+57.021464]GTPNYLSPEVLNK(+2)	69.24	74.24	943.441	357.159102	35.2
T[+79.966331]IC[+57.021464]GTPNYLSPEVLNK (heavy)(+2)	69.24	74.24	947.449	707.417765	30.2
T[+79.966331]IC[+57.021464]GTPNYLSPEVLNK (heavy)(+2)	69.24	74.24	947.449	382.253994	31.2
T[+79.966331]IC[+57.021464]GTPNYLSPEVLNK (heavy)(+2)	69.24	74.24	947.449	357.159102	35.2
ILGETS[+79.966331]LM[+15.994915]R(+2)	43.55	48.55	558.261	889.34851	18.7
ILGETS[+79.966331]LM[+15.994915]R(+2)	43.55	48.55	558.261	791.371614	21.7
ILGETS[+79.966331]LM[+15.994915]R(+2)	43.55	48.55	558.261	227.175404	19.7
ILGETS[+79.966331]LM[+15.994915]R (heavy)(+2)	43.55	48.55	563.266	899.356779	18.7
ILGETS[+79.966331]LM[+15.994915]R (heavy)(+2)	43.55	48.55	563.266	801.379883	21.7
ILGETS[+79.966331]LM[+15.994915]R (heavy)(+2)	43.55	48.55	563.266	227.175404	19.7
ILGETS[+79.966331]LMR(+2)	56.08	61.08	550.264	775.376699	22.4
ILGETS[+79.966331]LMR(+2)	56.08	61.08	550.264	488.264964	20.4
ILGETS[+79.966331]LMR(+2)	56.08	61.08	550.264	227.175404	18.4
ILGETS[+79.966331]LMR (heavy)(+2)	56.08	61.08	555.268	785.384968	22.4
ILGETS[+79.966331]LMR (heavy)(+2)	56.08	61.08	555.268	498.273233	20.4
ILGETS[+79.966331]LMR (heavy)(+2)	56.08	61.08	555.268	227.175404	18.4
IADFGLS[+79.966331]K(+2)	51.66	56.66	465.72	719.372266	19.9
IADFGLS[+79.966331]K(+2)	51.66	56.66	465.72	648.335152	18.9
IADFGLS[+79.966331]K(+2)	51.66	56.66	465.72	533.308209	20.9
IADFGLS[+79.966331]K (heavy)(+2)	51.66	56.66	469.727	727.386465	19.9
IADFGLS[+79.966331]K (heavy)(+2)	51.66	56.66	469.727	656.349351	18.9
IADFGLS[+79.966331]K (heavy)(+2)	51.66	56.66	469.727	541.322408	20.9
T[+79.966331]VC[+57.021464]GTPGYC[+57.021464]APEILR(+2)	63.91	68.91	887.388	1175.587755	26.5
T[+79.966331]VC[+57.021464]GTPGYC[+57.021464]APEILR(+2)	63.91	68.91	887.388	343.143452	33.5
T[+79.966331]VC[+57.021464]GTPGYC[+57.021464]APEILR(+2)	63.91	68.91	887.388	501.212594	28.5
T[+79.966331]VC[+57.021464]GTPGYC[+57.021464]APEILR (heavy)(+2)	63.91	68.91	892.392	1185.596024	26.5
T[+79.966331]VC[+57.021464]GTPGYC[+57.021464]APEILR (heavy)(+2)	63.91	68.91	892.392	343.143452	33.5
T[+79.966331]VC[+57.021464]GTPGYC[+57.021464]APEILR (heavy)(+2)	63.91	68.91	892.392	501.212594	28.5
T[+79.966331]YVGTNAYMAPER(+2)	48.83	53.83	776.828	1109.504419	24.2
T[+79.966331]YVGTNAYMAPER(+2)	48.83	53.83	776.828	401.214309	22.2
T[+79.966331]YVGTNAYMAPER(+2)	48.83	53.83	776.828	247.107718	30.2

T[+79.966331]YVGTNAYMAPER (heavy)(+2)	48.83	53.83	781.832	1119.512688	24.2
T[+79.966331]YVGTNAYMAPER (heavy)(+2)	48.83	53.83	781.832	411.222578	22.2
T[+79.966331]YVGTNAYMAPER (heavy)(+2)	48.83	53.83	781.832	247.107718	30.2
T[+79.966331]YVGTNAYM[+15.994915]APER(+2)	40.97	45.97	784.826	1061.501049	30.4
T[+79.966331]YVGTNAYM[+15.994915]APER(+2)	40.97	45.97	784.826	401.214309	26.4
T[+79.966331]YVGTNAYM[+15.994915]APER(+2)	40.97	45.97	784.826	247.107718	30.4
T[+79.966331]YVGTNAYM[+15.994915]APER (heavy)(+2)	40.97	45.97	789.83	1071.509318	30.4
T[+79.966331]YVGTNAYM[+15.994915]APER (heavy)(+2)	40.97	45.97	789.83	411.222578	26.4
T[+79.966331]YVGTNAYM[+15.994915]APER (heavy)(+2)	40.97	45.97	789.83	247.107718	30.4
TAC[+57.021464]TNFMMTPY[+79.966331]VVTR(+2)	70.3	75.3	936.398	1177.514529	30
TAC[+57.021464]TNFMMTPY[+79.966331]VVTR(+2)	70.3	75.3	936.398	915.43356	28
TAC[+57.021464]TNFMMTPY[+79.966331]VVTR(+2)	70.3	75.3	936.398	814.385882	29
TAC[+57.021464]TNFMMTPY[+79.966331]VVTR (heavy)(+2)	70.3	75.3	941.402	1187.522798	30
TAC[+57.021464]TNFMMTPY[+79.966331]VVTR (heavy)(+2)	70.3	75.3	941.402	925.441829	28
TAC[+57.021464]TNFMMTPY[+79.966331]VVTR (heavy)(+2)	70.3	75.3	941.402	824.394151	29
TAC[+57.021464]TNFM[+15.994915]MTPY[+79.966331]VVTR(+2)	60.99	65.99	944.395	1046.474045	31.2
TAC[+57.021464]TNFM[+15.994915]MTPY[+79.966331]VVTR(+2)	60.99	65.99	944.395	915.43356	31.2
TAC[+57.021464]TNFM[+15.994915]MTPY[+79.966331]VVTR(+2)	60.99	65.99	944.395	814.385882	31.2
TAC[+57.021464]TNFM[+15.994915]MTPY[+79.966331]VVTR (heavy)(+2)	60.99	65.99	949.399	1056.482314	31.2
TAC[+57.021464]TNFM[+15.994915]MTPY[+79.966331]VVTR (heavy)(+2)	60.99	65.99	949.399	925.441829	31.2
TAC[+57.021464]TNFM[+15.994915]MTPY[+79.966331]VVTR (heavy)(+2)	60.99	65.99	949.399	824.394151	31.2
TAC[+57.021464]TNFM[+15.994915]M[+15.994915]TPY[+79.966331]VVTR(+2)	49.92	54.92	952.393	1209.504359	30.5
TAC[+57.021464]TNFM[+15.994915]M[+15.994915]TPY[+79.966331]VVTR(+2)	49.92	54.92	952.393	915.43356	31.5
TAC[+57.021464]TNFM[+15.994915]M[+15.994915]TPY[+79.966331]VVTR(+2)	49.92	54.92	952.393	814.385882	34.5
TAC[+57.021464]TNFM[+15.994915]M[+15.994915]TPY[+79.966331]VVTR (heavy)(+2)	49.92	54.92	957.397	1219.512628	30.5
TAC[+57.021464]TNFM[+15.994915]M[+15.994915]TPY[+79.966331]VVTR (heavy)(+2)	49.92	54.92	957.397	925.441829	31.5
TAC[+57.021464]TNFM[+15.994915]M[+15.994915]TPY[+79.966331]VVTR (heavy)(+2)	49.92	54.92	957.397	824.394151	34.5
TAC[+57.021464]TNFMMT[+79.966331]PY[+79.966331]VVTR(+3)	77.68	82.68	651.256	1028.46348	25
TAC[+57.021464]TNFMMT[+79.966331]PY[+79.966331]VVTR(+3)	77.68	82.68	651.256	814.385882	22
TAC[+57.021464]TNFMMT[+79.966331]PY[+79.966331]VVTR(+3)	77.68	82.68	651.256	407.696579	24
TAC[+57.021464]TNFMMT[+79.966331]PY[+79.966331]VVTR (heavy)(+3)	77.68	82.68	654.592	1038.471749	25
TAC[+57.021464]TNFMMT[+79.966331]PY[+79.966331]VVTR (heavy)(+3)	77.68	82.68	654.592	824.394151	22
TAC[+57.021464]TNFMMT[+79.966331]PY[+79.966331]VVTR (heavy)(+3)	77.68	82.68	654.592	412.700713	24
TAC[+57.021464]TNFM[+15.994915]M[+15.994915]T[+79.966331]PY[+79.966331]VVTR(+3)	55.17	60.17	661.919	897.422995	27.4
TAC[+57.021464]TNFM[+15.994915]M[+15.994915]T[+79.966331]PY[+79.966331]VVTR(+3)	55.17	60.17	661.919	814.385882	27.4
TAC[+57.021464]TNFM[+15.994915]M[+15.994915]T[+79.966331]PY[+79.966331]VVTR(+3)	55.17	60.17	661.919	474.303458	27.4
TAC[+57.021464]TNFM[+15.994915]M[+15.994915]T[+79.966331]PY[+79.966331]VVTR (heavy)(+3)	55.17	60.17	665.255	907.431264	27.4
TAC[+57.021464]TNFM[+15.994915]M[+15.994915]T[+79.966331]PY[+79.966331]VVTR (heavy)(+3)	55.17	60.17	665.255	824.394151	27.4
TAC[+57.021464]TNFM[+15.994915]M[+15.994915]T[+79.966331]PY[+79.966331]VVTR (heavy)(+3)	55.17	60.17	665.255	484.311727	27.4
TAGTSFM[+15.994915]M[+15.994915]TPY[+79.966331]VVTR(+2)	49.51	54.51	887.383	915.43356	29.5

TAGTSFM[+15.994915]M[+15.994915]TPY[+79.966331]VVTR(+2)	49.51	54.51	887.383	814.385882	29.5
TAGTSFM[+15.994915]M[+15.994915]TPY[+79.966331]VVTR(+2)	49.51	54.51	887.383	375.235044	29.5
TAGTSFM[+15.994915]M[+15.994915]TPY[+79.966331]VVTR (heavy)(+2)	49.51	54.51	892.387	925.441829	29.5
TAGTSFM[+15.994915]M[+15.994915]TPY[+79.966331]VVTR (heavy)(+2)	49.51	54.51	892.387	824.394151	29.5
TAGTSFM[+15.994915]M[+15.994915]TPY[+79.966331]VVTR (heavy)(+2)	49.51	54.51	892.387	385.243313	29.5
TAGTSFMMT[+79.966331]PYVVTR(+2)	72.79	77.79	871.388	948.497149	33
TAGTSFMMT[+79.966331]PYVVTR(+2)	72.79	77.79	871.388	817.456664	26
TAGTSFMMT[+79.966331]PYVVTR(+2)	72.79	77.79	871.388	734.419551	31
TAGTSFMMT[+79.966331]PYVVTR(+2)	72.79	77.79	871.388	375.235044	29
TAGTSFMMT[+79.966331]PYVVTR (heavy)(+2)	72.79	77.79	876.392	958.505418	33
TAGTSFMMT[+79.966331]PYVVTR (heavy)(+2)	72.79	77.79	876.392	827.464933	26
TAGTSFMMT[+79.966331]PYVVTR (heavy)(+2)	72.79	77.79	876.392	744.42782	31
TAGTSFMMT[+79.966331]PYVVTR (heavy)(+2)	72.79	77.79	876.392	385.243313	29
TAGTSFM[+15.994915]M[+15.994915]T[+79.966331]PYVVTR(+2)	52.44	57.44	887.383	817.456664	29.5
TAGTSFM[+15.994915]M[+15.994915]T[+79.966331]PYVVTR(+2)	52.44	57.44	887.383	734.419551	29.5
TAGTSFM[+15.994915]M[+15.994915]T[+79.966331]PYVVTR(+2)	52.44	57.44	887.383	375.235044	29.5
TAGTSFM[+15.994915]M[+15.994915]T[+79.966331]PYVVTR(+2)	52.44	57.44	887.383	276.16663	29.5
TAGTSFM[+15.994915]M[+15.994915]T[+79.966331]PYVVTR (heavy)(+2)	52.44	57.44	892.387	827.464933	29.5
TAGTSFM[+15.994915]M[+15.994915]T[+79.966331]PYVVTR (heavy)(+2)	52.44	57.44	892.387	744.42782	29.5
TAGTSFM[+15.994915]M[+15.994915]T[+79.966331]PYVVTR (heavy)(+2)	52.44	57.44	892.387	385.243313	29.5
TAGTSFM[+15.994915]M[+15.994915]T[+79.966331]PYVVTR (heavy)(+2)	52.44	57.44	892.387	286.174899	29.5
TAGTSFMMT[+79.966331]PY[+79.966331]VVTR(+2)	79.49	84.49	911.371	1028.46348	34.2
TAGTSFMMT[+79.966331]PY[+79.966331]VVTR(+2)	79.49	84.49	911.371	897.422995	27.2
TAGTSFMMT[+79.966331]PY[+79.966331]VVTR(+2)	79.49	84.49	911.371	814.385882	32.2
TAGTSFMMT[+79.966331]PY[+79.966331]VVTR(+2)	79.49	84.49	911.371	375.235044	33.2
TAGTSFMMT[+79.966331]PY[+79.966331]VVTR (heavy)(+2)	79.49	84.49	916.375	1038.471749	34.2
TAGTSFMMT[+79.966331]PY[+79.966331]VVTR (heavy)(+2)	79.49	84.49	916.375	907.431264	27.2
TAGTSFMMT[+79.966331]PY[+79.966331]VVTR (heavy)(+2)	79.49	84.49	916.375	824.394151	32.2
TAGTSFMMT[+79.966331]PY[+79.966331]VVTR (heavy)(+2)	79.49	84.49	916.375	385.243313	33.2
TAGTSFMMTPY[+79.966331]VVTR(+2)	70.38	75.38	871.388	1046.474045	29
TAGTSFMMTPY[+79.966331]VVTR(+2)	70.38	75.38	871.388	915.43356	26
TAGTSFMMTPY[+79.966331]VVTR(+2)	70.38	75.38	871.388	814.385882	27
TAGTSFMMTPY[+79.966331]VVTR(+2)	70.38	75.38	871.388	474.303458	33
TAGTSFMMTPY[+79.966331]VVTR (heavy)(+2)	70.38	75.38	876.392	1056.482314	29
TAGTSFMMTPY[+79.966331]VVTR (heavy)(+2)	70.38	75.38	876.392	925.441829	26
TAGTSFMMTPY[+79.966331]VVTR (heavy)(+2)	70.38	75.38	876.392	824.394151	27
TAGTSFMMTPY[+79.966331]VVTR (heavy)(+2)	70.38	75.38	876.392	484.311727	33
TFC[+57.021464]GT[+79.966331]PDYIAPEIIAYQPYGK(+3)	87.59	92.59	828.711	826.40938	25.8
TFC[+57.021464]GT[+79.966331]PDYIAPEIIAYQPYGK(+3)	87.59	92.59	828.711	464.25036	34.8
TFC[+57.021464]GT[+79.966331]PDYIAPEIIAYQPYGK(+3)	87.59	92.59	828.711	204.134267	42.8
TFC[+57.021464]GT[+79.966331]PDYIAPEIIAYQPYGK(+3)	87.59	92.59	828.711	249.123368	33.8

TFC[+57.021464]GT[+79.966331]PDYIAPEIIAYQPYGK (heavy)(+3)	87.59	92.59	831.382	834.423579	25.8
TFC[+57.021464]GT[+79.966331]PDYIAPEIIAYQPYGK (heavy)(+3)	87.59	92.59	831.382	472.264559	34.8
TFC[+57.021464]GT[+79.966331]PDYIAPEIIAYQPYGK (heavy)(+3)	87.59	92.59	831.382	212.148466	42.8
TFC[+57.021464]GT[+79.966331]PDYIAPEIIAYQPYGK (heavy)(+3)	87.59	92.59	831.382	249.123368	33.8
T[+79.966331]FC[+57.021464]GTPDYIAPEIIAYQPYGK(+3)	89.59	94.59	828.711	826.40938	29.8
T[+79.966331]FC[+57.021464]GTPDYIAPEIIAYQPYGK(+3)	89.59	94.59	828.711	755.372266	30.8
T[+79.966331]FC[+57.021464]GTPDYIAPEIIAYQPYGK(+3)	89.59	94.59	828.711	464.25036	34.8
T[+79.966331]FC[+57.021464]GTPDYIAPEIIAYQPYGK (heavy)(+3)	89.59	94.59	831.382	834.423579	29.8
T[+79.966331]FC[+57.021464]GTPDYIAPEIIAYQPYGK (heavy)(+3)	89.59	94.59	831.382	763.386465	30.8
T[+79.966331]FC[+57.021464]GTPDYIAPEIIAYQPYGK (heavy)(+3)	89.59	94.59	831.382	472.264559	34.8
ILNHDT[+79.966331]SFAK(+2)	30.36	35.36	613.284	452.25036	24.3
ILNHDT[+79.966331]SFAK(+2)	30.36	35.36	613.284	556.742246	21.3
ILNHDT[+79.966331]SFAK(+2)	30.36	35.36	613.284	500.200214	23.3
ILNHDT[+79.966331]SFAK(+2)	30.36	35.36	613.284	451.211766	25.3
ILNHDT[+79.966331]SFAK (heavy)(+2)	30.36	35.36	617.291	460.264559	24.3
ILNHDT[+79.966331]SFAK (heavy)(+2)	30.36	35.36	617.291	560.749345	21.3
ILNHDT[+79.966331]SFAK (heavy)(+2)	30.36	35.36	617.291	504.207313	23.3
ILNHDT[+79.966331]SFAK (heavy)(+2)	30.36	35.36	617.291	455.218865	25.3
ILNHDTS[+79.966331]FAK(+2)	31.95	36.95	613.284	507.753798	20.3
ILNHDTS[+79.966331]FAK(+2)	31.95	36.95	613.284	451.211766	24.3
ILNHDTS[+79.966331]FAK(+2)	31.95	36.95	613.284	227.175404	24.3
ILNHDTS[+79.966331]FAK (heavy)(+2)	31.95	36.95	617.291	511.760897	20.3
ILNHDTS[+79.966331]FAK (heavy)(+2)	31.95	36.95	617.291	455.218865	24.3
ILNHDTS[+79.966331]FAK (heavy)(+2)	31.95	36.95	617.291	227.175404	24.3
ILNHDT[+79.966331]S[+79.966331]FAK(+2)	38.39	43.39	653.267	981.382587	18.8
ILNHDT[+79.966331]S[+79.966331]FAK(+2)	38.39	43.39	653.267	365.218332	18.8
ILNHDT[+79.966331]S[+79.966331]FAK(+2)	38.39	43.39	653.267	434.173468	18.8
ILNHDT[+79.966331]S[+79.966331]FAK (heavy)(+2)	38.39	43.39	657.274	989.396786	18.8
ILNHDT[+79.966331]S[+79.966331]FAK (heavy)(+2)	38.39	43.39	657.274	373.23253	18.8
ILNHDT[+79.966331]S[+79.966331]FAK (heavy)(+2)	38.39	43.39	657.274	438.180567	18.8
T[+79.966331]FVGTPYYMSPEQMNR(+2)	72.46	77.46	1000.917	992.428811	33.9
T[+79.966331]FVGTPYYMSPEQMNR(+2)	72.46	77.46	1000.917	774.356298	30.9
T[+79.966331]FVGTPYYMSPEQMNR(+2)	72.46	77.46	1000.917	330.181217	29.9
T[+79.966331]FVGTPYYMSPEQMNR (heavy)(+2)	72.46	77.46	1005.921	1002.43708	33.9
T[+79.966331]FVGTPYYMSPEQMNR (heavy)(+2)	72.46	77.46	1005.921	784.364567	30.9
T[+79.966331]FVGTPYYMSPEQMNR (heavy)(+2)	72.46	77.46	1005.921	330.181217	29.9
TFVGT[+79.966331]PYYMSPEQMNR(+2)	69.26	74.26	1000.917	992.428811	33.9
TFVGT[+79.966331]PYYMSPEQMNR(+2)	69.26	74.26	1000.917	861.388327	31.9
TFVGT[+79.966331]PYYMSPEQMNR(+2)	69.26	74.26	1000.917	774.356298	34.9
TFVGT[+79.966331]PYYMSPEQMNR (heavy)(+2)	69.26	74.26	1005.921	1002.43708	33.9
TFVGT[+79.966331]PYYMSPEQMNR (heavy)(+2)	69.26	74.26	1005.921	871.396596	31.9

TFVGT[+79.966331]PYYMSPEQMNR (heavy)(+2)	69.26	74.26	1005.921	784.364567	34.9
T[+79.966331]LC[+57.021464]GTPNYIAPEVLSK(+2)	69.5	74.5	921.939	743.429781	27.6
T[+79.966331]LC[+57.021464]GTPNYIAPEVLSK(+2)	69.5	74.5	921.939	672.392667	26.6
T[+79.966331]LC[+57.021464]GTPNYIAPEVLSK(+2)	69.5	74.5	921.939	357.159102	34.6
T[+79.966331]LC[+57.021464]GTPNYIAPEVLSK (heavy)(+2)	69.5	74.5	925.946	751.44398	27.6
T[+79.966331]LC[+57.021464]GTPNYIAPEVLSK (heavy)(+2)	69.5	74.5	925.946	680.406866	26.6
T[+79.966331]LC[+57.021464]GTPNYIAPEVLSK (heavy)(+2)	69.5	74.5	925.946	357.159102	34.6
TLC[+57.021464]GT[+79.966331]PNYIAPEVLSK(+2)	67.09	72.09	921.939	743.429781	30.6
TLC[+57.021464]GT[+79.966331]PNYIAPEVLSK(+2)	67.09	72.09	921.939	672.392667	29.6
TLC[+57.021464]GT[+79.966331]PNYIAPEVLSK(+2)	67.09	72.09	921.939	347.228896	33.6
TLC[+57.021464]GT[+79.966331]PNYIAPEVLSK (heavy)(+2)	67.09	72.09	925.946	751.44398	30.6
TLC[+57.021464]GT[+79.966331]PNYIAPEVLSK (heavy)(+2)	67.09	72.09	925.946	680.406866	29.6
TLC[+57.021464]GT[+79.966331]PNYIAPEVLSK (heavy)(+2)	67.09	72.09	925.946	355.243095	33.6
T[+79.966331]LC[+57.021464]GT[+79.966331]PNYIAPEVLSK(+2)	79.39	84.39	961.922	743.429781	28.8
T[+79.966331]LC[+57.021464]GT[+79.966331]PNYIAPEVLSK(+2)	79.39	84.39	961.922	672.392667	29.8
T[+79.966331]LC[+57.021464]GT[+79.966331]PNYIAPEVLSK(+2)	79.39	84.39	961.922	347.228896	32.8
T[+79.966331]LC[+57.021464]GT[+79.966331]PNYIAPEVLSK (heavy)(+2)	79.39	84.39	965.929	751.44398	28.8
T[+79.966331]LC[+57.021464]GT[+79.966331]PNYIAPEVLSK (heavy)(+2)	79.39	84.39	965.929	680.406866	29.8
T[+79.966331]LC[+57.021464]GT[+79.966331]PNYIAPEVLSK (heavy)(+2)	79.39	84.39	965.929	355.243095	32.8
TNT[+79.966331]FC[+57.021464]GTPDYIAPEILLGQK(+2)	91.02	96.02	1159.542	558.360973	46.7
TNT[+79.966331]FC[+57.021464]GTPDYIAPEILLGQK(+2)	91.02	96.02	1159.542	332.192845	45.7
TNT[+79.966331]FC[+57.021464]GTPDYIAPEILLGQK(+2)	91.02	96.02	1159.542	299.134995	46.7
TNT[+79.966331]FC[+57.021464]GTPDYIAPEILLGQK (heavy)(+2)	91.02	96.02	1163.549	566.375172	46.7
TNT[+79.966331]FC[+57.021464]GTPDYIAPEILLGQK (heavy)(+2)	91.02	96.02	1163.549	340.207044	45.7
TNT[+79.966331]FC[+57.021464]GTPDYIAPEILLGQK (heavy)(+2)	91.02	96.02	1163.549	299.134995	46.7
TQTSMS[+79.966331]LGTTR(+2)	35.05	40.05	631.775	935.461492	25.9
TQTSMS[+79.966331]LGTTR(+2)	35.05	40.05	631.775	747.381785	24.9
TQTSMS[+79.966331]LGTTR(+2)	35.05	40.05	631.775	230.113532	22.9
TQTSMS[+79.966331]LGTTR (heavy)(+2)	35.05	40.05	636.78	945.469761	25.9
TQTSMS[+79.966331]LGTTR (heavy)(+2)	35.05	40.05	636.78	757.390054	24.9
TQTSMS[+79.966331]LGTTR (heavy)(+2)	35.05	40.05	636.78	230.113532	22.9
TQTS[+79.966331]MSLGTTR(+2)	34.65	39.65	631.775	935.461492	25.9
TQTS[+79.966331]MSLGTTR(+2)	34.65	39.65	631.775	434.235772	24.9
TQTS[+79.966331]MSLGTTR(+2)	34.65	39.65	631.775	230.113532	24.9
TQTS[+79.966331]MSLGTTR (heavy)(+2)	34.65	39.65	636.78	945.469761	25.9
TQTS[+79.966331]MSLGTTR (heavy)(+2)	34.65	39.65	636.78	444.244041	24.9
TQTS[+79.966331]MSLGTTR (heavy)(+2)	34.65	39.65	636.78	230.113532	24.9
TSTFC[+57.021464]GT[+79.966331]PEFLAPEVLTETSYTR(+3)	92.96	97.96	896.403	970.484001	30.3
TSTFC[+57.021464]GT[+79.966331]PEFLAPEVLTETSYTR(+3)	92.96	97.96	896.403	857.399937	36.3
TSTFC[+57.021464]GT[+79.966331]PEFLAPEVLTETSYTR(+3)	92.96	97.96	896.403	290.134661	36.3
TSTFC[+57.021464]GT[+79.966331]PEFLAPEVLTETSYTR(+3)	92.96	97.96	896.403	437.203075	36.3

TSTFC[+57.021464]GT[+79.966331]PEFLAPEVLTETSYTR (heavy)(+3)	92.96	97.96	899.739	980.49227	30.3
TSTFC[+57.021464]GT[+79.966331]PEFLAPEVLTETSYTR (heavy)(+3)	92.96	97.96	899.739	867.408206	36.3
TSTFC[+57.021464]GT[+79.966331]PEFLAPEVLTETSYTR (heavy)(+3)	92.96	97.96	899.739	290.134661	36.3
TSTFC[+57.021464]GT[+79.966331]PEFLAPEVLTETSYTR (heavy)(+3)	92.96	97.96	899.739	437.203075	36.3
TST[+79.966331]FC[+57.021464]GTPEFLAPEVLTETSYTR(+3)	92.96	97.96	896.403	970.484001	36.3
TST[+79.966331]FC[+57.021464]GTPEFLAPEVLTETSYTR(+3)	92.96	97.96	896.403	857.399937	33.3
TST[+79.966331]FC[+57.021464]GTPEFLAPEVLTETSYTR(+3)	92.96	97.96	896.403	272.124096	36.3
TST[+79.966331]FC[+57.021464]GTPEFLAPEVLTETSYTR(+3)	92.96	97.96	896.403	419.19251	34.3
TST[+79.966331]FC[+57.021464]GTPEFLAPEVLTETSYTR (heavy)(+3)	92.96	97.96	899.739	980.49227	36.3
TST[+79.966331]FC[+57.021464]GTPEFLAPEVLTETSYTR (heavy)(+3)	92.96	97.96	899.739	867.408206	33.3
TST[+79.966331]FC[+57.021464]GTPEFLAPEVLTETSYTR (heavy)(+3)	92.96	97.96	899.739	272.124096	36.3
TST[+79.966331]FC[+57.021464]GTPEFLAPEVLTETSYTR (heavy)(+3)	92.96	97.96	899.739	419.19251	34.3
TSTFC[+57.021464]GT[+79.966331]PEFLAPEVLTQEAYTR(+3)	93.5	98.5	900.075	981.499986	32.5
TSTFC[+57.021464]GT[+79.966331]PEFLAPEVLTQEAYTR(+3)	93.5	98.5	900.075	868.415922	28.5
TSTFC[+57.021464]GT[+79.966331]PEFLAPEVLTQEAYTR(+3)	93.5	98.5	900.075	439.229959	33.5
TSTFC[+57.021464]GT[+79.966331]PEFLAPEVLTQEAYTR(+3)	93.5	98.5	900.075	290.134661	36.5
TSTFC[+57.021464]GT[+79.966331]PEFLAPEVLTQEAYTR (heavy)(+3)	93.5	98.5	903.411	991.508255	32.5
TSTFC[+57.021464]GT[+79.966331]PEFLAPEVLTQEAYTR (heavy)(+3)	93.5	98.5	903.411	878.424191	28.5
TSTFC[+57.021464]GT[+79.966331]PEFLAPEVLTQEAYTR (heavy)(+3)	93.5	98.5	903.411	449.238228	33.5
TSTFC[+57.021464]GT[+79.966331]PEFLAPEVLTQEAYTR (heavy)(+3)	93.5	98.5	903.411	290.134661	36.5
TST[+79.966331]FC[+57.021464]GTPEFLAPEVLTQEAYTR(+3)	93.5	98.5	900.075	1080.5684	33.5
TST[+79.966331]FC[+57.021464]GTPEFLAPEVLTQEAYTR(+3)	93.5	98.5	900.075	981.499986	33.5
TST[+79.966331]FC[+57.021464]GTPEFLAPEVLTQEAYTR(+3)	93.5	98.5	900.075	370.100992	38.5
TST[+79.966331]FC[+57.021464]GTPEFLAPEVLTQEAYTR (heavy)(+3)	93.5	98.5	903.411	1090.576669	33.5
TST[+79.966331]FC[+57.021464]GTPEFLAPEVLTQEAYTR (heavy)(+3)	93.5	98.5	903.411	991.508255	33.5
TST[+79.966331]FC[+57.021464]GTPEFLAPEVLTQEAYTR (heavy)(+3)	93.5	98.5	903.411	370.100992	38.5
T[+79.966331]TQMSAAGTYAWMAPEVIK(+2)	83.54	88.54	1068.48	656.397753	32
T[+79.966331]TQMSAAGTYAWMAPEVIK(+2)	83.54	88.54	1068.48	585.360639	32
T[+79.966331]TQMSAAGTYAWMAPEVIK(+2)	83.54	88.54	1068.48	260.196868	35
T[+79.966331]TQMSAAGTYAWMAPEVIK (heavy)(+2)	83.54	88.54	1072.487	664.411952	32
T[+79.966331]TQMSAAGTYAWMAPEVIK (heavy)(+2)	83.54	88.54	1072.487	593.374838	32
T[+79.966331]TQMSAAGTYAWMAPEVIK (heavy)(+2)	83.54	88.54	1072.487	268.211067	35
TVC[+57.021464]STY[+79.966331]LQSR(+2)	30.66	35.66	647.778	934.402988	21.3
TVC[+57.021464]STY[+79.966331]LQSR(+2)	30.66	35.66	647.778	746.323281	24.3
TVC[+57.021464]STY[+79.966331]LQSR(+2)	30.66	35.66	647.778	547.720456	22.3
TVC[+57.021464]STY[+79.966331]LQSR (heavy)(+2)	30.66	35.66	652.782	944.411257	21.3
TVC[+57.021464]STY[+79.966331]LQSR (heavy)(+2)	30.66	35.66	652.782	756.33155	24.3
TVC[+57.021464]STY[+79.966331]LQSR (heavy)(+2)	30.66	35.66	652.782	552.724591	22.3
TWT[+79.966331]LC[+57.021464]GTPEYLAPEIILSK(+2)	100.48	105.48	1136.552	799.492381	36
TWT[+79.966331]LC[+57.021464]GTPEYLAPEIILSK(+2)	100.48	105.48	1136.552	234.144832	34
TWT[+79.966331]LC[+57.021464]GTPEYLAPEIILSK(+2)	100.48	105.48	1136.552	288.134267	38

TWT[+79.966331]LC[+57.021464]GTPEYLAPEIILSK (heavy)(+2)	100.48	105.48	1140.559	807.50658	36
TWT[+79.966331]LC[+57.021464]GTPEYLAPEIILSK (heavy)(+2)	100.48	105.48	1140.559	242.159031	34
TWT[+79.966331]LC[+57.021464]GTPEYLAPEIILSK (heavy)(+2)	100.48	105.48	1140.559	288.134267	38
TWTLC[+57.021464]GT[+79.966331]PEYLAPEIILSK(+2)	100.48	105.48	1136.552	870.529495	30
TWTLC[+57.021464]GT[+79.966331]PEYLAPEIILSK(+2)	100.48	105.48	1136.552	460.31296	34
TWTLC[+57.021464]GT[+79.966331]PEYLAPEIILSK(+2)	100.48	105.48	1136.552	347.228896	41
TWTLC[+57.021464]GT[+79.966331]PEYLAPEIILSK(+2)	100.48	105.48	1136.552	719.318122	34
TWTLC[+57.021464]GT[+79.966331]PEYLAPEIILSK (heavy)(+2)	100.48	105.48	1140.559	878.543694	30
TWTLC[+57.021464]GT[+79.966331]PEYLAPEIILSK (heavy)(+2)	100.48	105.48	1140.559	468.327159	34
TWTLC[+57.021464]GT[+79.966331]PEYLAPEIILSK (heavy)(+2)	100.48	105.48	1140.559	355.243095	41
TWTLC[+57.021464]GT[+79.966331]PEYLAPEIILSK (heavy)(+2)	100.48	105.48	1140.559	719.318122	34
VADPDHDHTGFLTEY[+79.966331]VATR(+3)	49.76	54.76	741.995	919.392089	25.5
VADPDHDHTGFLTEY[+79.966331]VATR(+3)	49.76	54.76	741.995	689.301818	30.5
VADPDHDHTGFLTEY[+79.966331]VATR(+3)	49.76	54.76	741.995	347.203744	32.5
VADPDHDHTGFLTEY[+79.966331]VATR (heavy)(+3)	49.76	54.76	745.331	929.400358	25.5
VADPDHDHTGFLTEY[+79.966331]VATR (heavy)(+3)	49.76	54.76	745.331	699.310087	30.5
VADPDHDHTGFLTEY[+79.966331]VATR (heavy)(+3)	49.76	54.76	745.331	357.212013	32.5
VADPDHDHTGFLTEYVAT[+79.966331]R(+3)	59.46	64.46	741.995	821.415193	26.5
VADPDHDHTGFLTEYVAT[+79.966331]R(+3)	59.46	64.46	741.995	591.324922	29.5
VADPDHDHTGFLTEYVAT[+79.966331]R(+3)	59.46	64.46	741.995	427.170075	27.5
VADPDHDHTGFLTEYVAT[+79.966331]R(+3)	59.46	64.46	741.995	921.396348	27.5
VADPDHDHTGFLTEYVAT[+79.966331]R (heavy)(+3)	59.46	64.46	745.331	831.423462	26.5
VADPDHDHTGFLTEYVAT[+79.966331]R (heavy)(+3)	59.46	64.46	745.331	601.333191	29.5
VADPDHDHTGFLTEYVAT[+79.966331]R (heavy)(+3)	59.46	64.46	745.331	437.178344	27.5
VADPDHDHTGFLTEYVAT[+79.966331]R (heavy)(+3)	59.46	64.46	745.331	926.400482	27.5
VADPDHDHTGFLT[+79.966331]EYVATR(+3)	59.48	64.48	741.995	821.415193	27.5
VADPDHDHTGFLT[+79.966331]EYVATR(+3)	59.48	64.48	741.995	609.335487	31.5
VADPDHDHTGFLT[+79.966331]EYVATR(+3)	59.48	64.48	741.995	446.272158	29.5
VADPDHDHTGFLT[+79.966331]EYVATR (heavy)(+3)	59.48	64.48	745.331	831.423462	27.5
VADPDHDHTGFLT[+79.966331]EYVATR (heavy)(+3)	59.48	64.48	745.331	619.343756	31.5
VADPDHDHTGFLT[+79.966331]EYVATR (heavy)(+3)	59.48	64.48	745.331	456.280427	29.5
VDNEDIY[+79.966331]ESR(+2)	28.14	33.14	660.261	747.307297	23.7
VDNEDIY[+79.966331]ESR(+2)	28.14	33.14	660.261	634.223233	20.7
VDNEDIY[+79.966331]ESR(+2)	28.14	33.14	660.261	262.15098	23.7
VDNEDIY[+79.966331]ESR (heavy)(+2)	28.14	33.14	665.265	757.315566	23.7
VDNEDIY[+79.966331]ESR (heavy)(+2)	28.14	33.14	665.265	644.231502	20.7
VDNEDIY[+79.966331]ESR (heavy)(+2)	28.14	33.14	665.265	272.159249	23.7
TYT[+79.966331]HEVVTLWYR(+3)	69	74	549.923	738.393336	20.2
TYT[+79.966331]HEVVTLWYR(+3)	69	74	549.923	524.261593	20.2
TYT[+79.966331]HEVVTLWYR(+3)	69	74	549.923	265.118283	22.2
TYT[+79.966331]HEVVTLWYR(+3)	69	74	549.923	712.233798	22.2

TYT[+79.966331]HEVVTLWYR (heavy)(+3)	69	74	553.259	748.401605	20.2
TYT[+79.966331]HEVVTLWYR (heavy)(+3)	69	74	553.259	534.269862	20.2
TYT[+79.966331]HEVVTLWYR (heavy)(+3)	69	74	553.259	265.118283	22.2
TYT[+79.966331]HEVVTLWYR (heavy)(+3)	69	74	553.259	712.233798	22.2
VIEDDPEAVY[+79.966331]TTTGGK(+2)	44.04	49.04	887.892	807.328426	29.5
VIEDDPEAVY[+79.966331]TTTGGK(+2)	44.04	49.04	887.892	564.298767	25.5
VIEDDPEAVY[+79.966331]TTTGGK(+2)	44.04	49.04	887.892	342.202347	30.5
VIEDDPEAVY[+79.966331]TTTGGK (heavy)(+2)	44.04	49.04	891.899	815.342625	29.5
VIEDDPEAVY[+79.966331]TTTGGK (heavy)(+2)	44.04	49.04	891.899	572.312966	25.5
VIEDDPEAVY[+79.966331]TTTGGK (heavy)(+2)	44.04	49.04	891.899	342.202347	30.5
VIEDNEY[+79.966331]TAR(+2)	24.66	29.66	645.274	590.233404	23.3
VIEDNEY[+79.966331]TAR(+2)	24.66	29.66	645.274	347.203744	24.3
VIEDNEY[+79.966331]TAR(+2)	24.66	29.66	645.274	213.159754	22.3
VIEDNEY[+79.966331]TAR (heavy)(+2)	24.66	29.66	650.278	600.241673	23.3
VIEDNEY[+79.966331]TAR (heavy)(+2)	24.66	29.66	650.278	357.212013	24.3
VIEDNEY[+79.966331]TAR (heavy)(+2)	24.66	29.66	650.278	213.159754	22.3
VLEDDPEAAY[+79.966331]TTR(+2)	39.76	44.76	780.334	988.413553	27.3
VLEDDPEAAY[+79.966331]TTR(+2)	39.76	44.76	780.334	377.214309	24.3
VLEDDPEAAY[+79.966331]TTR(+2)	39.76	44.76	780.334	213.159754	26.3
VLEDDPEAAY[+79.966331]TTR (heavy)(+2)	39.76	44.76	785.339	998.421822	27.3
VLEDDPEAAY[+79.966331]TTR (heavy)(+2)	39.76	44.76	785.339	387.222578	24.3
VLEDDPEAAY[+79.966331]TTR (heavy)(+2)	39.76	44.76	785.339	213.159754	26.3
VLEDDPEAAY[+79.966331]TTTGGK(+2)	39.78	44.78	873.877	1175.498011	26.1
VLEDDPEAAY[+79.966331]TTTGGK(+2)	39.78	44.78	873.877	878.36554	29.1
VLEDDPEAAY[+79.966331]TTTGGK(+2)	39.78	44.78	873.877	564.298767	26.1
VLEDDPEAAY[+79.966331]TTTGGK (heavy)(+2)	39.78	44.78	877.884	1183.51221	26.1
VLEDDPEAAY[+79.966331]TTTGGK (heavy)(+2)	39.78	44.78	877.884	886.379739	29.1
VLEDDPEAAY[+79.966331]TTTGGK (heavy)(+2)	39.78	44.78	877.884	572.312966	26.1
HPGHYAVYNLS[+79.966331]PR(+3)	37.53	42.53	530.911	568.320171	20.5
HPGHYAVYNLS[+79.966331]PR(+3)	37.53	42.53	530.911	272.171716	20.5
HPGHYAVYNLS[+79.966331]PR(+3)	37.53	42.53	530.911	429.199327	23.5
HPGHYAVYNLS[+79.966331]PR (heavy)(+3)	37.53	42.53	534.248	578.32844	20.5
HPGHYAVYNLS[+79.966331]PR (heavy)(+3)	37.53	42.53	534.248	282.179985	20.5
HPGHYAVYNLS[+79.966331]PR (heavy)(+3)	37.53	42.53	534.248	429.199327	23.5
DESEVSDEGGS[+79.966331]PISSEGQEPR(+3)	41.75	46.75	757.635	889.401	28.1
DESEVSDEGGS[+79.966331]PISSEGQEPR(+3)	41.75	46.75	757.635	802.368972	27.1
DESEVSDEGGS[+79.966331]PISSEGQEPR(+3)	41.75	46.75	757.635	272.171716	38.1
DESEVSDEGGS[+79.966331]PISSEGQEPR (heavy)(+3)	41.75	46.75	760.972	899.409269	28.1
DESEVSDEGGS[+79.966331]PISSEGQEPR (heavy)(+3)	41.75	46.75	760.972	812.377241	27.1
DESEVSDEGGS[+79.966331]PISSEGQEPR (heavy)(+3)	41.75	46.75	760.972	282.179985	38.1
DESEVS[+79.966331]DEGGSPISSEGQEPR(+3)	44.14	49.14	757.635	889.401	24.1

DESEVS[+79.966331]DEGGSPISSEGQEPR(+3)	44.14	49.14	757.635	802.368972	32.1
DESEVS[+79.966331]DEGGSPISSEGQEPR(+3)	44.14	49.14	757.635	272.171716	36.1
DESEVS[+79.966331]DEGGSPISSEGQEPR (heavy)(+3)	44.14	49.14	760.972	899.409269	24.1
DESEVS[+79.966331]DEGGSPISSEGQEPR (heavy)(+3)	44.14	49.14	760.972	812.377241	32.1
DESEVS[+79.966331]DEGGSPISSEGQEPR (heavy)(+3)	44.14	49.14	760.972	282.179985	36.1
VS[+79.966331]ENDFEDLLS[+79.966331]NQGFSSR(+2)	95.27	100.27	1102.432	1090.563983	40
VS[+79.966331]ENDFEDLLS[+79.966331]NQGFSSR(+2)	95.27	100.27	1102.432	977.479919	35
VS[+79.966331]ENDFEDLLS[+79.966331]NQGFSSR(+2)	95.27	100.27	1102.432	864.395855	34
VS[+79.966331]ENDFEDLLS[+79.966331]NQGFSSR (heavy)(+2)	95.27	100.27	1107.436	1100.572252	40
VS[+79.966331]ENDFEDLLS[+79.966331]NQGFSSR (heavy)(+2)	95.27	100.27	1107.436	987.488188	35
VS[+79.966331]ENDFEDLLS[+79.966331]NQGFSSR (heavy)(+2)	95.27	100.27	1107.436	874.404124	34
VS[+79.966331]ENDFEDLLSNQGFSSR(+2)	86.47	91.47	1062.449	882.40642	40.8
VS[+79.966331]ENDFEDLLSNQGFSSR(+2)	86.47	91.47	1062.449	349.183009	37.8
VS[+79.966331]ENDFEDLLSNQGFSSR(+2)	86.47	91.47	1062.449	674.278031	37.8
VS[+79.966331]ENDFEDLLSNQGFSSR (heavy)(+2)	86.47	91.47	1067.453	892.414689	40.8
VS[+79.966331]ENDFEDLLSNQGFSSR (heavy)(+2)	86.47	91.47	1067.453	359.191278	37.8
VS[+79.966331]ENDFEDLLSNQGFSSR (heavy)(+2)	86.47	91.47	1067.453	674.278031	37.8
VVDFGSATFDHEHHSTIVST[+79.966331]R(+3)	50.44	55.44	808.032	1063.487941	30
VVDFGSATFDHEHHSTIVST[+79.966331]R(+3)	50.44	55.44	808.032	932.440262	30
VVDFGSATFDHEHHSTIVST[+79.966331]R(+3)	50.44	55.44	808.032	314.171047	33
VVDFGSATFDHEHHSTIVST[+79.966331]R (heavy)(+3)	50.44	55.44	811.368	1068.492075	30
VVDFGSATFDHEHHSTIVST[+79.966331]R (heavy)(+3)	50.44	55.44	811.368	937.444397	30
VVDFGSATFDHEHHSTIVST[+79.966331]R (heavy)(+3)	50.44	55.44	811.368	314.171047	33
VYT[+79.966331]HEVVTLWYR(+3)	70.71	75.71	549.263	738.393336	20.2
VYT[+79.966331]HEVVTLWYR(+3)	70.71	75.71	549.263	524.261593	20.2
VYT[+79.966331]HEVVTLWYR(+3)	70.71	75.71	549.263	338.18228	22.2
VYT[+79.966331]HEVVTLWYR (heavy)(+3)	70.71	75.71	552.6	748.401605	20.2
VYT[+79.966331]HEVVTLWYR (heavy)(+3)	70.71	75.71	552.6	534.269862	20.2
VYT[+79.966331]HEVVTLWYR (heavy)(+3)	70.71	75.71	552.6	348.190549	22.2
SPEVLLGS[+79.966331]AR(+2)	46.48	51.48	554.773	598.367121	22.5
SPEVLLGS[+79.966331]AR(+2)	46.48	51.48	554.773	372.198993	21.5
SPEVLLGS[+79.966331]AR(+2)	46.48	51.48	554.773	314.134661	23.5
SPEVLLGS[+79.966331]AR (heavy)(+2)	46.48	51.48	559.777	608.37539	22.5
SPEVLLGS[+79.966331]AR (heavy)(+2)	46.48	51.48	559.777	382.207262	21.5
SPEVLLGS[+79.966331]AR (heavy)(+2)	46.48	51.48	559.777	314.134661	23.5
VYT[+79.966331]YIQSR(+2)	44.08	49.08	555.254	749.394064	22.6
VYT[+79.966331]YIQSR(+2)	44.08	49.08	555.254	666.35695	22.6
VYT[+79.966331]YIQSR(+2)	44.08	49.08	555.254	503.293622	23.6
VYT[+79.966331]YIQSR (heavy)(+2)	44.08	49.08	560.259	759.402333	22.6
VYT[+79.966331]YIQSR (heavy)(+2)	44.08	49.08	560.259	676.365219	22.6
VYT[+79.966331]YIQSR (heavy)(+2)	44.08	49.08	560.259	513.301891	23.6

VYTY[+79.966331]IQSR(+2)	32.21	37.21	555.254	847.37096	19.6
VYTY[+79.966331]IQSR(+2)	32.21	37.21	555.254	746.323281	21.6
VYTY[+79.966331]IQSR(+2)	32.21	37.21	555.254	503.293622	23.6
VYTY[+79.966331]IQSR(+2)	32.21	37.21	555.254	263.139019	18.6
VYTY[+79.966331]IQSR (heavy)(+2)	32.21	37.21	560.259	857.379229	19.6
VYTY[+79.966331]IQSR (heavy)(+2)	32.21	37.21	560.259	756.33155	21.6
VYTY[+79.966331]IQSR (heavy)(+2)	32.21	37.21	560.259	513.301891	23.6
VYTY[+79.966331]IQSR (heavy)(+2)	32.21	37.21	560.259	263.139019	18.6
VYT[+79.966331]Y[+79.966331]IQSR(+2)	45.82	50.82	595.238	829.360395	23.8
VYT[+79.966331]Y[+79.966331]IQSR(+2)	45.82	50.82	595.238	746.323281	21.8
VYT[+79.966331]Y[+79.966331]IQSR(+2)	45.82	50.82	595.238	503.293622	22.8
VYT[+79.966331]Y[+79.966331]IQSR (heavy)(+2)	45.82	50.82	600.242	839.368664	23.8
VYT[+79.966331]Y[+79.966331]IQSR (heavy)(+2)	45.82	50.82	600.242	756.33155	21.8
VYT[+79.966331]Y[+79.966331]IQSR (heavy)(+2)	45.82	50.82	600.242	513.301891	22.8
LIEDNEY[+79.966331]TAR(+2)	30.27	35.27	652.281	590.233404	23.5
LIEDNEY[+79.966331]TAR(+2)	30.27	35.27	652.281	347.203744	26.5
LIEDNEY[+79.966331]TAR(+2)	30.27	35.27	652.281	227.175404	21.5
LIEDNEY[+79.966331]TAR (heavy)(+2)	30.27	35.27	657.286	600.241673	23.5
LIEDNEY[+79.966331]TAR (heavy)(+2)	30.27	35.27	657.286	357.212013	26.5
LIEDNEY[+79.966331]TAR (heavy)(+2)	30.27	35.27	657.286	227.175404	21.5
WTAPEAALY[+79.966331]GR(+2)	55.17	60.17	657.797	956.423724	20.6
WTAPEAALY[+79.966331]GR(+2)	55.17	60.17	657.797	730.328367	26.6
WTAPEAALY[+79.966331]GR(+2)	55.17	60.17	657.797	659.291253	23.6
WTAPEAALY[+79.966331]GR(+2)	55.17	60.17	657.797	475.170075	25.6
WTAPEAALY[+79.966331]GR (heavy)(+2)	55.17	60.17	662.801	966.431993	20.6
WTAPEAALY[+79.966331]GR (heavy)(+2)	55.17	60.17	662.801	740.336636	26.6
WTAPEAALY[+79.966331]GR (heavy)(+2)	55.17	60.17	662.801	669.299522	23.6
WTAPEAALY[+79.966331]GR (heavy)(+2)	55.17	60.17	662.801	485.178344	25.6
VLEDDPEAT[+79.966331]Y[+79.966331]TTSGGK(+2)	42.32	47.32	921.857	1271.459257	27.6
VLEDDPEAT[+79.966331]Y[+79.966331]TTSGGK(+2)	42.32	47.32	921.857	1173.482361	32.6
VLEDDPEAT[+79.966331]Y[+79.966331]TTSGGK(+2)	42.32	47.32	921.857	449.235438	29.6
VLEDDPEAT[+79.966331]Y[+79.966331]TTSGGK (heavy)(+2)	42.32	47.32	925.864	1279.473456	27.6
VLEDDPEAT[+79.966331]Y[+79.966331]TTSGGK (heavy)(+2)	42.32	47.32	925.864	1181.49656	32.6
VLEDDPEAT[+79.966331]Y[+79.966331]TTSGGK (heavy)(+2)	42.32	47.32	925.864	457.249637	29.6
WTAPEAISY[+79.966331]R(+2)	53.7	58.7	637.284	915.397175	20
WTAPEAISY[+79.966331]R(+2)	53.7	58.7	637.284	689.301818	25
WTAPEAISY[+79.966331]R(+2)	53.7	58.7	637.284	505.18064	23
WTAPEAISY[+79.966331]R(+2)	53.7	58.7	637.284	418.148611	24
WTAPEAISY[+79.966331]R (heavy)(+2)	53.7	58.7	642.288	925.405444	20
WTAPEAISY[+79.966331]R (heavy)(+2)	53.7	58.7	642.288	699.310087	25
WTAPEAISY[+79.966331]R (heavy)(+2)	53.7	58.7	642.288	515.188909	23

WTAPEAISY[+79.966331]R (heavy)(+2)	53.7	58.7	642.288	428.15688	24
WTAPEAIS[+79.966331]YR(+2)	56.9	61.9	637.284	888.457392	25
WTAPEAIS[+79.966331]YR(+2)	56.9	61.9	637.284	817.420279	25
WTAPEAIS[+79.966331]YR(+2)	56.9	61.9	637.284	591.324922	26
WTAPEAIS[+79.966331]YR(+2)	56.9	61.9	637.284	338.18228	26
WTAPEAIS[+79.966331]YR (heavy)(+2)	56.9	61.9	642.288	898.465661	25
WTAPEAIS[+79.966331]YR (heavy)(+2)	56.9	61.9	642.288	827.428548	25
WTAPEAIS[+79.966331]YR (heavy)(+2)	56.9	61.9	642.288	601.333191	26
WTAPEAIS[+79.966331]YR (heavy)(+2)	56.9	61.9	642.288	348.190549	26
WTAPEAIS[+79.966331]Y[+79.966331]R(+2)	60.78	65.78	677.267	897.38661	25.2
WTAPEAIS[+79.966331]Y[+79.966331]R(+2)	60.78	65.78	677.267	671.291253	27.2
WTAPEAIS[+79.966331]Y[+79.966331]R(+2)	60.78	65.78	677.267	600.254139	27.2
WTAPEAIS[+79.966331]Y[+79.966331]R(+2)	60.78	65.78	677.267	336.149264	24.2
WTAPEAIS[+79.966331]Y[+79.966331]R (heavy)(+2)	60.78	65.78	682.271	907.394879	25.2
WTAPEAIS[+79.966331]Y[+79.966331]R (heavy)(+2)	60.78	65.78	682.271	681.299522	27.2
WTAPEAIS[+79.966331]Y[+79.966331]R (heavy)(+2)	60.78	65.78	682.271	610.262408	27.2
WTAPEAIS[+79.966331]Y[+79.966331]R (heavy)(+2)	60.78	65.78	682.271	341.153399	24.2
ADENY[+79.966331]YK(+2)	18.33	23.33	491.681	796.291313	15.7
ADENY[+79.966331]YK(+2)	18.33	23.33	491.681	667.248719	18.7
ADENY[+79.966331]YK(+2)	18.33	23.33	491.681	553.205792	18.7
ADENY[+79.966331]YK(+2)	18.33	23.33	491.681	310.176132	18.7
ADENY[+79.966331]YK (heavy)(+2)	18.33	23.33	495.688	804.305512	15.7
ADENY[+79.966331]YK (heavy)(+2)	18.33	23.33	495.688	675.262918	18.7
ADENY[+79.966331]YK (heavy)(+2)	18.33	23.33	495.688	561.219991	18.7
ADENY[+79.966331]YK (heavy)(+2)	18.33	23.33	495.688	318.190331	18.7
ADENYY[+79.966331]K(+2)	18.33	23.33	491.681	796.291313	16.7
ADENYY[+79.966331]K(+2)	18.33	23.33	491.681	667.248719	17.7
ADENYY[+79.966331]K(+2)	18.33	23.33	491.681	553.205792	18.7
ADENYY[+79.966331]K(+2)	18.33	23.33	491.681	390.142463	21.7
ADENYY[+79.966331]K (heavy)(+2)	18.33	23.33	495.688	804.305512	16.7
ADENYY[+79.966331]K (heavy)(+2)	18.33	23.33	495.688	675.262918	17.7
ADENYY[+79.966331]K (heavy)(+2)	18.33	23.33	495.688	561.219991	18.7
ADENYY[+79.966331]K (heavy)(+2)	18.33	23.33	495.688	398.156662	21.7
WYAPEC[+57.021464]INY[+79.966331]YK(+2)	64.15	69.15	793.822	667.248719	26.7
WYAPEC[+57.021464]INY[+79.966331]YK(+2)	64.15	69.15	793.822	553.205792	30.7
WYAPEC[+57.021464]INY[+79.966331]YK(+2)	64.15	69.15	793.822	310.176132	26.7
WYAPEC[+57.021464]INY[+79.966331]YK(+2)	64.15	69.15	793.822	350.149918	26.7
WYAPEC[+57.021464]INY[+79.966331]YK (heavy)(+2)	64.15	69.15	797.83	675.262918	26.7
WYAPEC[+57.021464]INY[+79.966331]YK (heavy)(+2)	64.15	69.15	797.83	561.219991	30.7
WYAPEC[+57.021464]INY[+79.966331]YK (heavy)(+2)	64.15	69.15	797.83	318.190331	26.7
WYAPEC[+57.021464]INY[+79.966331]YK (heavy)(+2)	64.15	69.15	797.83	350.149918	26.7

Y[+79.966331]TC[+57.021464]QIK(+2)	18.45	23.45	446.685	649.333772	18.3
Y[+79.966331]TC[+57.021464]QIK(+2)	18.45	23.45	446.685	548.286094	15.3
Y[+79.966331]TC[+57.021464]QIK(+2)	18.45	23.45	446.685	260.196868	15.3
Y[+79.966331]TC[+57.021464]QIK (heavy)(+2)	18.45	23.45	450.692	657.347971	18.3
Y[+79.966331]TC[+57.021464]QIK (heavy)(+2)	18.45	23.45	450.692	556.300293	15.3
Y[+79.966331]TC[+57.021464]QIK (heavy)(+2)	18.45	23.45	450.692	268.211067	15.3
VAGS[+79.966331]QPITVAWYK(+2)	67.05	72.05	750.368	977.545479	26.4
VAGS[+79.966331]QPITVAWYK(+2)	67.05	72.05	750.368	767.408652	25.4
VAGS[+79.966331]QPITVAWYK(+2)	67.05	72.05	750.368	425.214308	26.4
VAGS[+79.966331]QPITVAWYK (heavy)(+2)	67.05	72.05	754.375	985.559678	26.4
VAGS[+79.966331]QPITVAWYK (heavy)(+2)	67.05	72.05	754.375	775.422851	25.4
VAGS[+79.966331]QPITVAWYK (heavy)(+2)	67.05	72.05	754.375	425.214308	26.4
VAGSQPIT[+79.966331]VAWYK(+2)	65.45	70.45	750.368	959.534914	28.4
VAGSQPIT[+79.966331]VAWYK(+2)	65.45	70.45	750.368	567.292559	23.4
VAGSQPIT[+79.966331]VAWYK(+2)	65.45	70.45	750.368	496.255445	25.4
VAGSQPIT[+79.966331]VAWYK (heavy)(+2)	65.45	70.45	754.375	967.549113	28.4
VAGSQPIT[+79.966331]VAWYK (heavy)(+2)	65.45	70.45	754.375	575.306758	23.4
VAGSQPIT[+79.966331]VAWYK (heavy)(+2)	65.45	70.45	754.375	504.269644	25.4
VAGS[+79.966331]QPIT[+79.966331]VAWYK(+2)	79.05	84.05	790.351	959.534914	29.6
VAGS[+79.966331]QPIT[+79.966331]VAWYK(+2)	79.05	84.05	790.351	567.292559	26.6
VAGS[+79.966331]QPIT[+79.966331]VAWYK(+2)	79.05	84.05	790.351	496.255445	25.6
VAGS[+79.966331]QPIT[+79.966331]VAWYK(+2)	79.05	84.05	790.351	310.176132	27.6
VAGS[+79.966331]QPIT[+79.966331]VAWYK (heavy)(+2)	79.05	84.05	794.358	967.549113	29.6
VAGS[+79.966331]QPIT[+79.966331]VAWYK (heavy)(+2)	79.05	84.05	794.358	575.306758	26.6
VAGS[+79.966331]QPIT[+79.966331]VAWYK (heavy)(+2)	79.05	84.05	794.358	504.269644	25.6
VAGS[+79.966331]QPIT[+79.966331]VAWYK (heavy)(+2)	79.05	84.05	794.358	318.190331	27.6
YM[+15.994915]EDSTY[+79.966331]YK(+2)	26.79	31.79	648.23	985.355035	22.4
YM[+15.994915]EDSTY[+79.966331]YK(+2)	26.79	31.79	648.23	741.285499	24.4
YM[+15.994915]EDSTY[+79.966331]YK(+2)	26.79	31.79	648.23	311.106004	21.4
YM[+15.994915]EDSTY[+79.966331]YK (heavy)(+2)	26.79	31.79	652.237	993.369234	22.4
YM[+15.994915]EDSTY[+79.966331]YK (heavy)(+2)	26.79	31.79	652.237	749.299698	24.4
YM[+15.994915]EDSTY[+79.966331]YK (heavy)(+2)	26.79	31.79	652.237	311.106004	21.4
YMEDSTY[+79.966331]YK(+2)	34.51	39.51	640.233	985.355035	18.1
YMEDSTY[+79.966331]YK(+2)	34.51	39.51	640.233	856.312442	19.1
YMEDSTY[+79.966331]YK(+2)	34.51	39.51	640.233	295.111089	22.1
YMEDSTY[+79.966331]YK (heavy)(+2)	34.51	39.51	644.24	993.369234	18.1
YMEDSTY[+79.966331]YK (heavy)(+2)	34.51	39.51	644.24	864.326641	19.1
YMEDSTY[+79.966331]YK (heavy)(+2)	34.51	39.51	644.24	295.111089	22.1
YM[+15.994915]EDSTYY[+79.966331]K(+2)	27.87	32.87	648.23	985.355035	21.4
YM[+15.994915]EDSTYY[+79.966331]K(+2)	27.87	32.87	648.23	390.142463	26.4
YM[+15.994915]EDSTYY[+79.966331]K(+2)	27.87	32.87	648.23	311.106004	21.4

YM[+15.994915]EDSTYY[+79.966331]K (heavy)(+2)	27.87	32.87	652.237	993.369234	21.4
YM[+15.994915]EDSTYY[+79.966331]K (heavy)(+2)	27.87	32.87	652.237	398.156662	26.4
YM[+15.994915]EDSTYY[+79.966331]K (heavy)(+2)	27.87	32.87	652.237	311.106004	21.4
YMEDSTYY[+79.966331]K(+2)	33.71	38.71	640.233	985.355035	18.1
YMEDSTYY[+79.966331]K(+2)	33.71	38.71	640.233	390.142463	26.1
YMEDSTYY[+79.966331]K(+2)	33.71	38.71	640.233	295.111089	20.1
YMEDSTYY[+79.966331]K (heavy)(+2)	33.71	38.71	644.24	993.369234	18.1
YMEDSTYY[+79.966331]K (heavy)(+2)	33.71	38.71	644.24	398.156662	26.1
YMEDSTYY[+79.966331]K (heavy)(+2)	33.71	38.71	644.24	295.111089	20.1
Y[+79.966331]MEDSTYYK(+2)	36.51	41.51	640.233	905.388704	21.1
Y[+79.966331]MEDSTYYK(+2)	36.51	41.51	640.233	776.346111	21.1
Y[+79.966331]MEDSTYYK(+2)	36.51	41.51	640.233	375.07742	19.1
Y[+79.966331]MEDSTYYK (heavy)(+2)	36.51	41.51	644.24	913.402903	21.1
Y[+79.966331]MEDSTYYK (heavy)(+2)	36.51	41.51	644.24	784.36031	21.1
Y[+79.966331]MEDSTYYK (heavy)(+2)	36.51	41.51	644.24	375.07742	19.1
Y[+79.966331]M[+15.994915]EDSTYYK(+2)	30.93	35.93	648.23	905.388704	23.4
Y[+79.966331]M[+15.994915]EDSTYYK(+2)	30.93	35.93	648.23	310.176132	22.4
Y[+79.966331]M[+15.994915]EDSTYYK(+2)	30.93	35.93	648.23	327.07405	24.4
Y[+79.966331]M[+15.994915]EDSTYYK (heavy)(+2)	30.93	35.93	652.237	913.402903	23.4
Y[+79.966331]M[+15.994915]EDSTYYK (heavy)(+2)	30.93	35.93	652.237	318.190331	22.4
Y[+79.966331]M[+15.994915]EDSTYYK (heavy)(+2)	30.93	35.93	652.237	327.07405	24.4
YIEDEDY[+79.966331]YK(+2)	39.05	44.05	659.249	1041.344864	20.7
YIEDEDY[+79.966331]YK(+2)	39.05	44.05	659.249	912.302271	22.7
YIEDEDY[+79.966331]YK(+2)	39.05	44.05	659.249	277.154669	22.7
YIEDEDY[+79.966331]YK (heavy)(+2)	39.05	44.05	663.256	1049.359063	20.7
YIEDEDY[+79.966331]YK (heavy)(+2)	39.05	44.05	663.256	920.31647	22.7
YIEDEDY[+79.966331]YK (heavy)(+2)	39.05	44.05	663.256	277.154669	22.7
YIEDEDYY[+79.966331]K(+2)	37.85	42.85	659.249	1041.344864	21.7
YIEDEDYY[+79.966331]K(+2)	37.85	42.85	659.249	390.142463	26.7
YIEDEDYY[+79.966331]K(+2)	37.85	42.85	659.249	277.154669	20.7
YIEDEDYY[+79.966331]K (heavy)(+2)	37.85	42.85	663.256	1049.359063	21.7
YIEDEDYY[+79.966331]K (heavy)(+2)	37.85	42.85	663.256	398.156662	26.7
YIEDEDYY[+79.966331]K (heavy)(+2)	37.85	42.85	663.256	277.154669	20.7
YIEDEDY[+79.966331]Y[+79.966331]K(+2)	39.48	44.48	699.232	1121.311195	20.9
YIEDEDY[+79.966331]Y[+79.966331]K(+2)	39.48	44.48	699.232	992.268602	21.9
YIEDEDY[+79.966331]Y[+79.966331]K(+2)	39.48	44.48	699.232	277.154669	24.9
YIEDEDY[+79.966331]Y[+79.966331]K (heavy)(+2)	39.48	44.48	703.24	1129.325394	20.9
YIEDEDY[+79.966331]Y[+79.966331]K (heavy)(+2)	39.48	44.48	703.24	1000.282801	21.9
YIEDEDY[+79.966331]Y[+79.966331]K (heavy)(+2)	39.48	44.48	703.24	277.154669	24.9
YVLDDEY[+79.966331]TSSVGSK(+2)	46.17	51.17	821.847	665.346445	29.6
YVLDDEY[+79.966331]TSSVGSK(+2)	46.17	51.17	821.847	291.166296	28.6

YVLDDEY[+79.966331]TSSVGSK(+2)	46.17	51.17	821.847	263.139019	29.6
YVLDDEY[+79.966331]TSSVGSK (heavy)(+2)	46.17	51.17	825.854	673.360644	29.6
YVLDDEY[+79.966331]TSSVGSK (heavy)(+2)	46.17	51.17	825.854	299.180495	28.6
YVLDDEY[+79.966331]TSSVGSK (heavy)(+2)	46.17	51.17	825.854	263.139019	29.6
YVLDDEY[+79.966331]VSSFGAK(+2)	65.08	70.08	836.86	695.372266	27
YVLDDEY[+79.966331]VSSFGAK(+2)	65.08	70.08	836.86	596.303852	31
YVLDDEY[+79.966331]VSSFGAK(+2)	65.08	70.08	836.86	263.139019	29
YVLDDEY[+79.966331]VSSFGAK (heavy)(+2)	65.08	70.08	840.867	703.386465	27
YVLDDEY[+79.966331]VSSFGAK (heavy)(+2)	65.08	70.08	840.867	604.318051	31
YVLDDEY[+79.966331]VSSFGAK (heavy)(+2)	65.08	70.08	840.867	263.139019	29
YVLDDQY[+79.966331]TSSSGAK(+2)	38.44	43.44	807.34	880.344805	28.1
YVLDDQY[+79.966331]TSSSGAK(+2)	38.44	43.44	807.34	637.315145	26.1
YVLDDQY[+79.966331]TSSSGAK(+2)	38.44	43.44	807.34	263.139019	30.1
YVLDDQY[+79.966331]TSSSGAK (heavy)(+2)	38.44	43.44	811.347	888.359004	28.1
YVLDDQY[+79.966331]TSSSGAK (heavy)(+2)	38.44	43.44	811.347	645.329344	26.1
YVLDDQY[+79.966331]TSSSGAK (heavy)(+2)	38.44	43.44	811.347	263.139019	30.1
YVLDDQY[+79.966331]VSSVGTK(+2)	53.37	58.37	827.374	920.41249	27.7
YVLDDQY[+79.966331]VSSVGTK(+2)	53.37	58.37	827.374	578.314417	27.7
YVLDDQY[+79.966331]VSSVGTK(+2)	53.37	58.37	827.374	263.139019	30.7
YVLDDQY[+79.966331]VSSVGTK (heavy)(+2)	53.37	58.37	831.381	928.426689	27.7
YVLDDQY[+79.966331]VSSVGTK (heavy)(+2)	53.37	58.37	831.381	586.328616	27.7
YVLDDQY[+79.966331]VSSVGTK (heavy)(+2)	53.37	58.37	831.381	263.139019	30.7
GAGSSEPVTGLDAK(+2)	29.16	34.16	644.822	1016.525866	22.2
GAGSSEPVTGLDAK(+2)	29.16	34.16	644.822	800.451245	22.2
GAGSSEPVTGLDAK(+2)	29.16	34.16	644.822	604.330067	22.2
VEATFGVDESNAK(+2)	36.25	41.25	683.827	966.452701	23.4
VEATFGVDESNAK(+2)	36.25	41.25	683.827	819.384287	23.4
VEATFGVDESNAK(+2)	36.25	41.25	683.827	229.118283	23.4
YILAGVENSK(+2)	39.52	44.52	547.298	817.441408	19.3
YILAGVENSK(+2)	39.52	44.52	547.298	633.32023	19.3
YILAGVENSK(+2)	39.52	44.52	547.298	277.154669	19.3
LGGNEQVTR(+2)	17.28	22.28	487.256	860.42207	17.5
LGGNEQVTR(+2)	17.28	22.28	487.256	503.293622	17.5
LGGNEQVTR(+2)	17.28	22.28	487.256	171.112804	17.5
TPVISGGPYEYR(+2)	45.58	50.58	669.838	1041.499986	23
TPVISGGPYEYR(+2)	45.58	50.58	669.838	928.415922	23
TPVISGGPYEYR(+2)	45.58	50.58	669.838	298.176132	23
TPVITGAPYEYR(+2)	48.14	53.14	683.853	956.447222	23.4
TPVITGAPYEYR(+2)	48.14	53.14	683.853	855.399543	23.4
TPVITGAPYEYR(+2)	48.14	53.14	683.853	298.176132	23.4
DGLDAASYYAPVR(+2)	56.56	61.56	699.338	926.473043	23.9

DGLDAASYYAPVR(+2)	56.56	61.56	699.338	855.435929	23.9
DGLDAASYYAPVR(+2)	56.56	61.56	699.338	605.340572	23.9
ADVTPADFSEWSK(+2)	59.77	64.77	726.835	1066.484001	24.7
ADVTPADFSEWSK(+2)	59.77	64.77	726.835	286.139747	24.7
ADVTPADFSEWSK(+2)	59.77	64.77	726.835	387.187425	24.7
GTFIIDPGGVIR(+2)	66.75	71.75	622.853	826.478128	21.6
GTFIIDPGGVIR(+2)	66.75	71.75	622.853	598.367121	21.6
GTFIIDPGGVIR(+2)	66.75	71.75	622.853	288.203016	21.6
GTFIIDPAAVIR(+2)	73.96	78.96	636.869	854.509428	22
GTFIIDPAAVIR(+2)	73.96	78.96	636.869	741.425364	22
GTFIIDPAAVIR(+2)	73.96	78.96	636.869	626.398421	22
LFLQFGAQGSPFLK(+2)	81.53	86.53	776.929	1051.557107	26.2
LFLQFGAQGSPFLK(+2)	81.53	86.53	776.929	904.488693	26.2
LFLQFGAQGSPFLK(+2)	81.53	86.53	776.929	504.318046	26.2
